# Supplementary material for: Endothelial SOCS3 maintains homeostasis and promotes survival in endotoxemic mice
Source: JCI Insight. 2021 Jul 22;6(14):e147280. doi: 10.1172/jci.insight.147280 (PMC8410050; doi:10.1172/jci.insight.147280)

Supplemental methods

Unbiased clustering and principal component analysis of mouse gene expression data

Graphs from mouse data for unsupervised clustering, principal component analysis and cross-correlation heatmaps with missing data were generated in R V4.0.2 following the workflow described below. The complete datasets (“All data.csv” and “Log2fold_data.csv” are provided on Supplemental Tables 6 and 7, respectively.

```
library(missMDA)
library(VIM)
library(FactoMineR)
library(factoextra)
library (pheatmap)

#load the cormat file to get the correlation matrix function
source("http://www.sthda.com/upload/rquery_cormat.r")

alldata <- read.csv ("All data.csv",stringsAsFactor=FALSE)
rownames (alldata) <- alldata[,1]
numdata <- alldata[,c(11,12,18:38,40:166)]
group <- alldata[, 5]

nb <- estim_ncpPCA(numdata,method.cv = "Kfold", verbose = FALSE)

res.comp <- imputePCA(numdata, ncp = nb$ncp)
res.comp$completeObs

imp <- cbind.data.frame(res.comp$completeObs,group)
res.pca <- PCA(imp, quali.sup = 151, ncp = nb$ncp, graph=FALSE)

pdf("plots.pdf")
plot(res.pca, hab=151, lab="quali") # colors by group
fviz_pca_ind(res.pca, habillage=151, legend.title = "Groups", palette="lancet", repel = TRUE,
geom = "point") # colors by group

# 30 most important variables colored by contribution
fviz_pca_var(res.pca, repel = TRUE, select.var = list(cos2 = 30), col.var = "contrib",
gradient.cols = c("#00AFBB", "#E7B800", "#FC4E07"))

contribs <- res.pca$var$contrib[,1:2]
contribs <- contribs[order(contribs[,1],decreasing=TRUE),]

rquery.cormat(numdata, graphType="heatmap")
corrmatrix<-rquery.cormat(numdata, type="flatten", graph=FALSE)
write.table (corrmatrix$r, file = "corrmatrix.txt", append = FALSE, quote = FALSE, sep = "\t",
eol = "\n", na = "NA", dec = ".", row.names = TRUE, col.names = TRUE)

LPS_numdata <- imp[grep("LPS", imp$group), ]
LPS_numdata <- LPS_numdata[,1:148]

rquery.cormat(LPS_numdata, graphType="heatmap")
LPS_corrmatrix<-rquery.cormat(LPS_numdata, type="flatten", graph=FALSE)
write.table (LPS_corrmatrix$r, file = "LPS_corrmatrix.txt", append = FALSE, quote = FALSE, sep =
"\t", eol = "\n", na = "NA", dec = ".", row.names = TRUE, col.names = TRUE)

logdata <- read.csv ("Log2fold_data.csv")
numlogdata <- logdata [,6:132]
rownames(numlogdata) <- logdata [,1]
head (numlogdata)

numdata_scale = scale(numlogdata)
pheatmap(numdata_scale,main = "pheatmap default", na_col="gray")

dev.off()
```

Gel Electrophoresis and Immunoblotting

Confluent HUVEC were lysed in Laemmli buffer containing the following protease and phosphatase inhibitors: cOmplete protease inhibitor mixture, PhosSTOP phosphatase inhibitor mixture, 0.1 M NaF, 0.1 mM phenyl arsine oxide, 10 mM pyrophosphate and 0.1 mM pervanadate. After boiling, a total of 15 µl of cell lysate per lane was loaded on standard SDS-PAGE gels and transferred to nitrocellulose membranes. Immunoblots were performed by blocking the membranes with 5% nonfat dry milk or 5% BSA in PBS/Tween and incubating overnight at 4°C with respective primary antibodies. Secondary HRP-conjugated anti-mouse, anti-rabbit, or anti-goat antibodies were incubated for 1 h at room temperature. Membranes were developed via chemiluminescence detected with either a LAS-3000 (Fujifilm) or a Chemidoc MP (Bio-Rad) imaging systems.

RNA isolation and RT-qPCR

RNA isolation from whole organs: Approximately 30-50mg of a target organ was removed and placed into an RNAse free 2ml tube containing zirconia/silicone beads and 1ml Trizol reagent. Tissue was homogenized using a Mini-Bead beater-16 (BioSpec) for 1 minute. Homogenate was transferred to a new RNAse free tube and the RNA isolation from Trizol was continued as per manufacturer's instructions.

RNA isolation from HUVEC: Confluent monolayers grown in multi-well plates were lysed with Trizol reagent and total RNA was isolated following manufacturer's instructions.

RT-qPCR: 400 ng of total RNA were used to prepare cDNA using Primescript RT Master Mix at 42°C following manufacturer's instructions. cDNA was diluted 10-fold in nuclease-free water, and then, 2µL of cDNA were used per PCR reaction. qPCR was performed in a StepOnePlus (Applied Biosystems) instrument using SYBR green-based iTaq supermix and 2 pmol primers (Thermo Fisher). Fold induction was calculated using the ΔΔCt method using GAPDH as housekeeping gene (HUVEC) or HPRT (mouse RNA).

Lung histological scoring

5 µm sections from FFPE lungs were processed for standard H&E and scanned with a Hammamatsu C10730-12 Nanozoomer at 40x. Scans identified only by mouse ID number were scored by a single investigator (A.J.) that remained masked to experimental groups (both treatment and genotype) until all slides were scored. Inflammatory infiltration, intra alveolar and interstitial exudate accumulation, tissue necrosis, septal and alveolar wall thickening, microhemorrhage and thrombosis were all scored in a scale of 1-4 (1 being the least and 4 being the most abnormal); in four different areas identified first with lower and then higher magnification. These magnifications were variably selected by the operator as needed to make the observation based in the dynamic view provided by the software.

Immunohistochemistry

5 µm FFPE sections from lung, liver of kidney were processed for colorimetric histochemistry. Briefly, slides were deparaffinated and rehydrated in sequential steps in xylene and ethanol solutions (100%/ 95%/70%). Endogenous peroxidase activity was blocked with 0.5% H_2O_2/MeOH for 10 minutes at room temperature (RT) and antigen retrieval (EZ-Retriever System, Biogenex) was done for 15 minutes at 98 C in 10 mM citrate buffer, pH 6 (for F4/80) or

in 1mM EDTA pH 8 (for MPO). Samples were blocked with FBS for 1 hour at room temperature. Primary antibodies were incubated at a dilution of 1:200 (F4/80) or 1:500 (MPO) in PBS overnight at 4 C. Biotinylated anti-rabbit secondary antibodies 1:500 in PBS were incubated for 1 hour at RT. Then, samples were incubated with avidin/biotin peroxidase (Elite ABC-HRP, Vector) in the dark for 30 minutes at RT and signal was detected with 3,3'-diaminobenzidine (Immpact DAB, Vector) and counterstained with hematoxylin prior to dehydration and mounting with VectaMount. Whole slides were scanned with a Hammamatsu C10730-12 Nanozoomer at 40x.

Flow cytometry

Mice were euthanized with an overdose of pentobarbital. The chest cavity was opened, and 100 µl of blood were collected from a nicked aorta into EDTA-containing vials. Lungs were excised, washed several times with PBS, and minced finely with scissors. Then, the minced lungs were transferred to a 50ml conical tube containing 6 ml of pre-warmed collagenase type I/dispase II/DNAse I mix (CDD) and incubated at 37 ºC with gentle agitation for 1 hour. Single cell suspensions were obtained after the suspensions were passaged multiple times through 20-gauge cannulas and filtered through a 70 µm mesh. Cells were then spun at 400 g for 8 minutes at 4 ºC and resuspended in 3 ml of cold PBS containing 0.1% BSA.

In parallel, bone marrow cells were collected by flushing the right femur with 10ml PBS onto a 70 µm cell strainer and then centrifuged at 6000 rpm for 5 min 4ºC. Red blood cells from blood and bone marrow cells were lysed using RBC lysis buffer according to manufacturer's instructions. Live and dead cells from lungs, blood and bone marrow were distinguished using Pacific blue Annexin V Apoptosis Detection kit with 7-AAD. Lung single cell suspensions (300 µl / sample) were then stained with ly6G-APC-Cy7 to label neutrophils. Cells were then subjected to flow cytometry analysis using a BD FACS symphony. 30,000 events were acquired for each sample and the data was analyzed using FlowJo v10.7.1. Single cells were gated using FSC-H vs FSC-A, dead cells were excluded based on 7AAD-Annexin-V and only live cells were gated for further analysis. Results are expressed as percentage of total cells.

Isolation of RNA from spleen CD45+ cells

Control, SOCS3iEKO or heterozygous mice were treated with tamoxifen to induce Cre activation as described in the main text. Two weeks after, mice were sacrificed to collect spleens. Organs were then washed in PBS. One third of each spleen was transferred into a tube with Trizol to have RNA of total organ isolated later following the Trizol protocol. The remaining portion of each spleen was pressed with a plunger from a 10 cc syringe. Released cells and tissue remains were collected and pushed through 70 µm cell strainers to obtain single cells isolates. Cells were centrifuged at 400g for 8 min at 4 ºC. The cell pellet was then resuspended in 1.5 ml ice-cold PBS+0.1%BSA. The cell suspension was incubated with anti-CD45 conjugated Dynabeads M280 streptavidin beads (3 µg ab,300 µg beads/sample) for 10 minutes at RT under gentle rotation. CD45+ cells were separated using a magnetic separator (Dynal MPC-S) and washed six times with PBS+0.1%BSA. Cells were placed on magnet for 3 minutes, the supernatant was removed and cells were lysed in Trizol reagent. RNA was extracted following a modified protocol. Briefly, 0.1 ml chloroform was added to 0.5 ml of Trizol per sample. After a 2 min incubation, tubes were centrifuged for 15 min at 12,000 g at 4 ºC. The

aqueous solution was then recovered, mixed 1:1 with 70% EtOH, and applied to a silica column (RNeasy Plus Micro kit, Qiagen). Then, the columns were washed, and the RNA was eluted following the RNeasy Plus Micro standard protocol. Efficient gene deletion was confirmed by tail snip genotyping as described in the main text.

ELISA

HUVEC cells were cultured in six-well plates at a density of 7.2×10^5 cells/well. 14 hours before treatment, the media was reduced to 800 μL/well. Cells were then treated with IL6+R or vehicle control (PBS). Culture media was collected after 6 hours and centrifuged at 18,000 g for 10 min at 4 ˚C. Then, supernatant was aliquoted and stored at -80˚C until analysis. The levels of IFNα, IFNβ and IFNγ in culture media were measured using ELISA kits according to the manufacturer's instructions.

Gene knockdown

HUVEC were transfected by plating cells onto multi-well plates containing individual small interference RNA (siRNA) against SOCS3 or a non-targeting control pool complexed with lipofectamine RNAiMAX transfection reagent in suspension and seeded at 10^5 cells/cm^2. Knockdown efficiency was determined by Western blot analysis.

Supplemental Figure 1. Enrichment of CD45 cells from spleen. Spleens from control, heterozygous or SOCS3iEKO mice (corresponding to those shown in Figure 1C) were collected 14 days after tamoxifen treatment. CD45 cells were isolated using magnetic beads prior to RNA extraction and RT-qPCR. Enrichment was confirmed by a 5-10-fold increase in CD45 expression and >10-fold decrease in VWF expression compared to total RNA from the same spleens. NS: not statistically significant (Kruskal-Wallis). Data combined from three independent experiments.

Supplemental Figure 2. Mild liver dysfunction in endotoxemic SOCS3^{iEKO}. A, H&E staining showing a reduction in glycogen content but no other overt histological features in endotoxemic SOCS3^{iEKO} mice. B, Plasma albumin levels remained similar among all experimental groups (two-way ANOVA). Data combined from at least three independent experiments.

Supplemental Figure 3. Gene expression changes in HUVEC treated with IL-6+R. A, IL-6+R-mediated loss of barrier function requires continuous protein synthesis. B, RT-qPCR of IL-6+R-treated HUVEC. Data was obtained as described in Figure 7. Data combined from three independent experiments. C, RT-qPCR of cells treated with or without IL-6+R after infection with lentivirus to overexpress WT SOCS3 or K6Q-SOCS3. An empty vector lentivirus was used as control. Two-way ANOVA. Combined data from three independent experiments performed in duplicate each.

Supplemental Figure 4. Multiple significant parameters correlate with the severity score and plasma IL-6 levels. A, Cross-correlation analysis for all parameters measured in LPS-treated mice (full data available as Supplemental Table 6). B, Graph showing all significantly correlated parameters to the severity score or plasma IL-6. C, Examples of the correlation of expression of specific genes (expressed as a base 2 logarithm of fold changes) with the severity score for each mouse. Data combined from three independent experiments.

Supplemental Figure 5. Type I IFN-like, adhesive and prothrombotic gene expression in endotoxemic SOCS3^{iEKO} mice. RT-qPCR analysis of whole lung (A) or liver (B) RNA levels. Two-way ANOVA and Holm-Sidak post-hoc tests comparing het and SOCS3^{iEKO} mice to control within saline or LPS-treated groups. Asterisks denote $p < 0.05$. Data combined from at least three independent experiments.

Supplemental Figure 6. Neutrophil and monocyte infiltration in endotoxemic organs. A, flow cytometry analysis of Ly6G+ cells in lungs. Asterisks denote $p < 0.05$. Data from two independent experiments (n=7 saline- and n=9 LPS-treated mice). B, Immunohistochemical staining of FFPE sections for myeloperoxidase (MPO, a neutrophil marker) and F4/80 (a monocyte marker) in lungs and kidneys. Bars, 50 µm. Images are representative from three independent experiments.

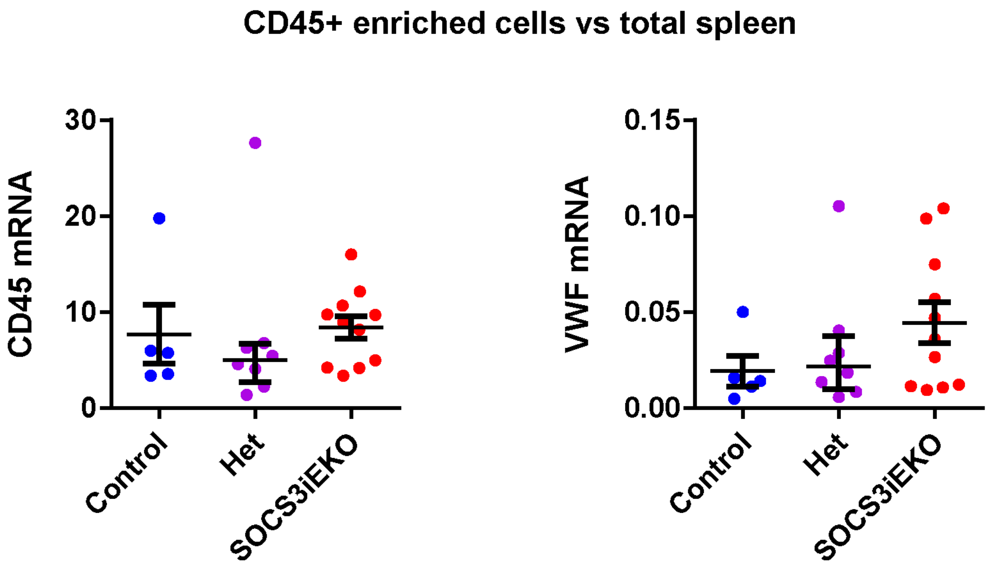

Supplemental Figure 1. Enrichment of CD45 cells from spleen. Spleens from control, heterozygous or SOCS3iEKO mice (corresponding to those shown in Figure 1C) were collected 14 days after tamoxifen treatment. CD45 cells were isolated using magnetic beads prior to RNA extraction and RT-qPCR. Enrichment was confirmed by a 5-10-fold increase in CD45 expression and >10-fold decrease in VWF expression compared to total RNA from the same spleens. NS: not statistically significant (Kruskal-Wallis). Data combined from three independent experiments.

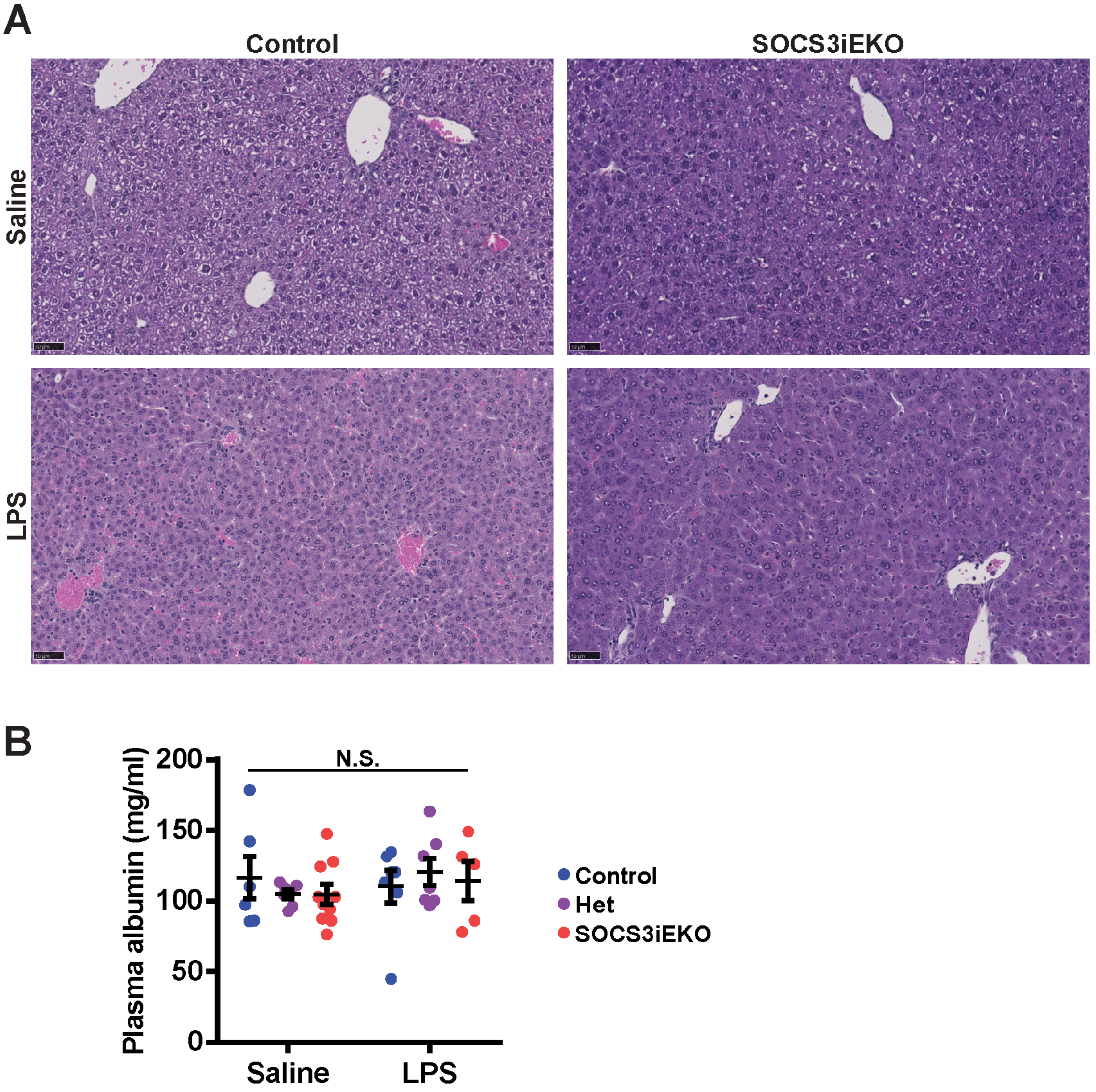

Supplemental Figure 2. Mild liver dysfunction in endotoxemic SOCS3^{iEKO}. A, H&E staining showing a reduction in glycogen content but no other overt histological features in endotoxemic SOCS3^{iEKO} mice. B, Plasma albumin levels remained similar among all experimental groups (two-way ANOVA). Data combined from at least three independent experiments.

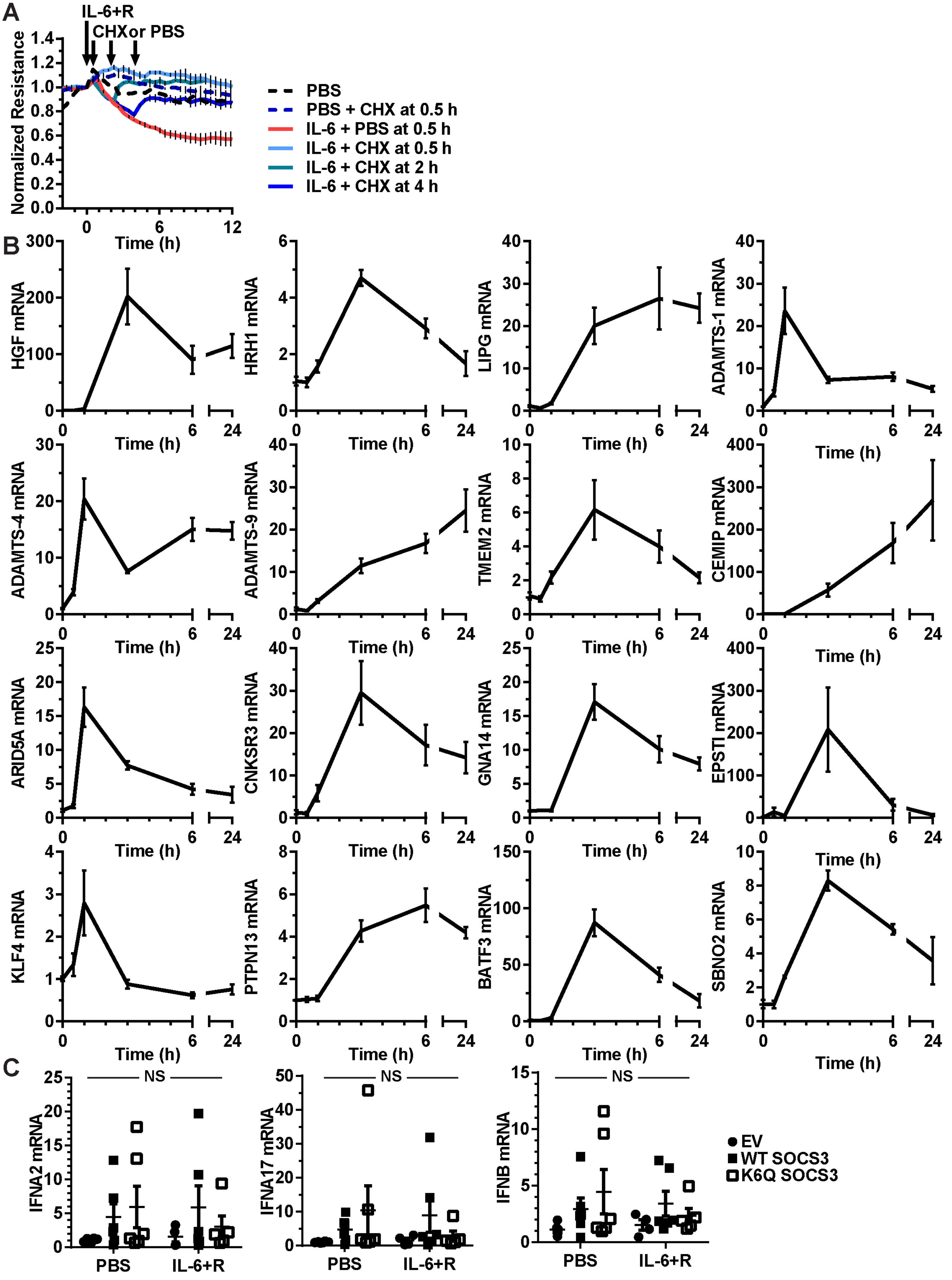

Supplemental Figure 3. Gene expression changes in HUVEC treated with IL-6+R. A, IL-6+R-mediated loss of barrier function requires continuous protein synthesis. B, RT-qPCR of IL-6+R-treated HUVEC. Data was obtained as described in Figure 7. Data combined from three independent experiments. C, RT-qPCR of cells treated with or without IL-6+R after infection with lentivirus to overexpress WT SOCS3 or K6Q-SOCS3. An empty vector lentivirus was used as control. Two-way ANOVA. Combined data from three independent experiments performed in duplicate each.

Cross-correlation analysis - only LPS mice

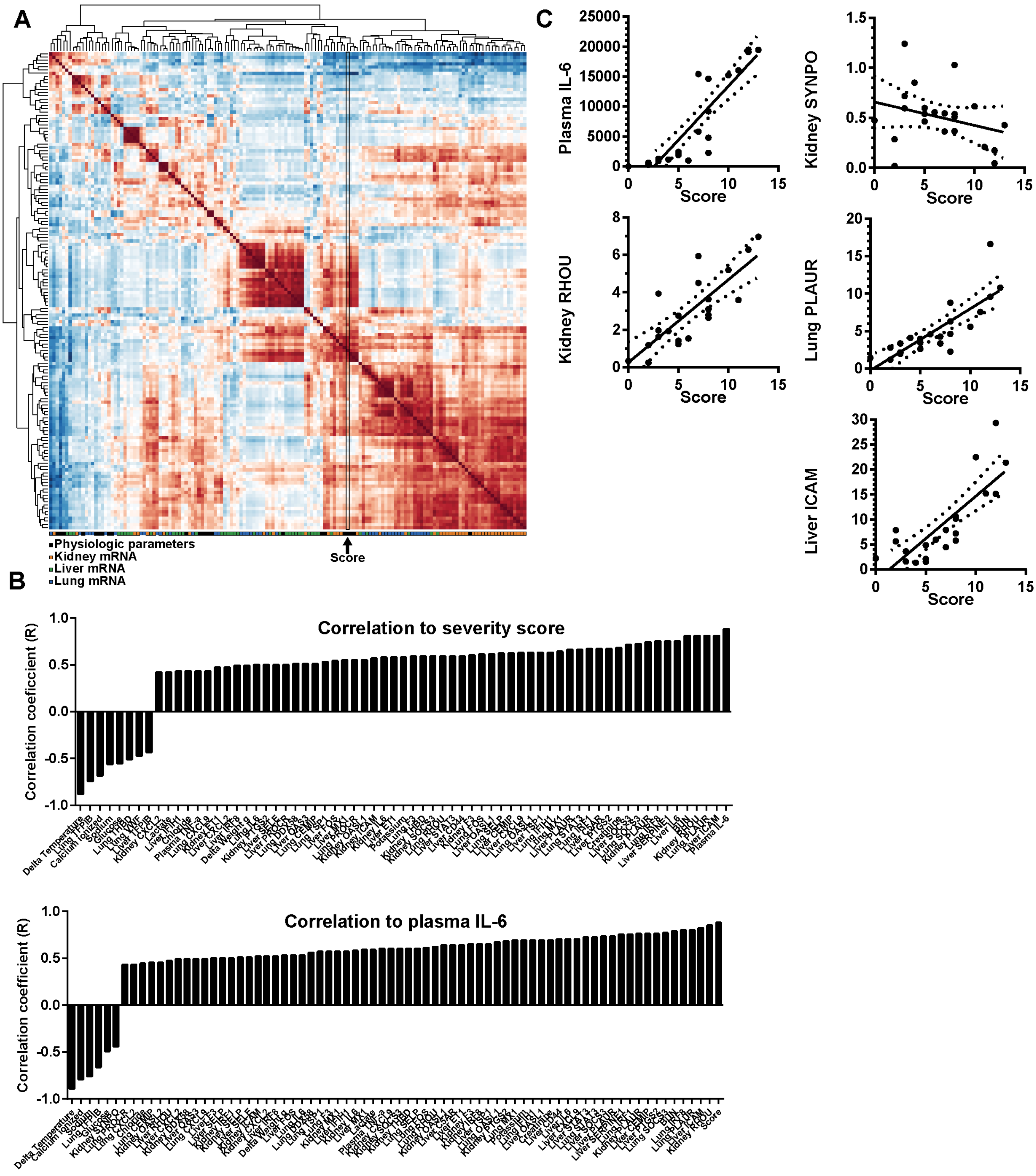

Supplemental Figure 4. Multiple significant parameters correlate with the severity score and plasma IL-6 levels. A, Cross-correlation analysis for all parameters measured in LPS-treated mice (full data available as Supplemental Table 6). B, Graph showing all significantly correlated parameters to the severity score or plasma IL-6. C, Examples of the correlation of expression of specific genes (expressed as a base 2 logarithm of fold changes) with the severity score for each mouse. Data combined from three independent experiments.

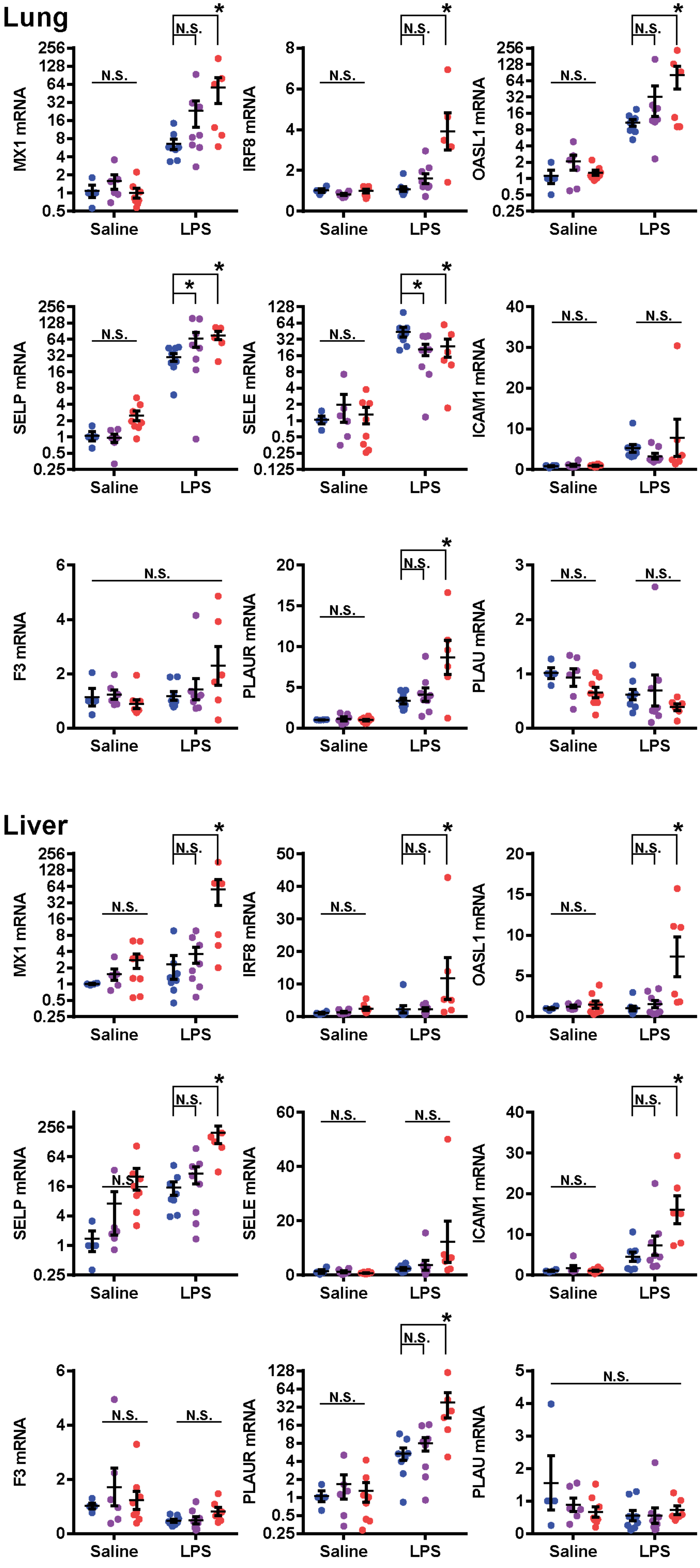

Supplemental Figure 5. Type I IFN-like, adhesive and prothrombotic gene expression in endotoxemic SOCS3^{iEKO} mice. RT-qPCR analysis of whole lung (A) or liver (B) RNA levels. Two-way ANOVA and Holm-Sidak post-hoc tests comparing het and SOCS3^{iEKO} mice to control within saline or LPS-treated groups. Asterisks denote $p < 0.05$. Data combined from at least three independent experiments.

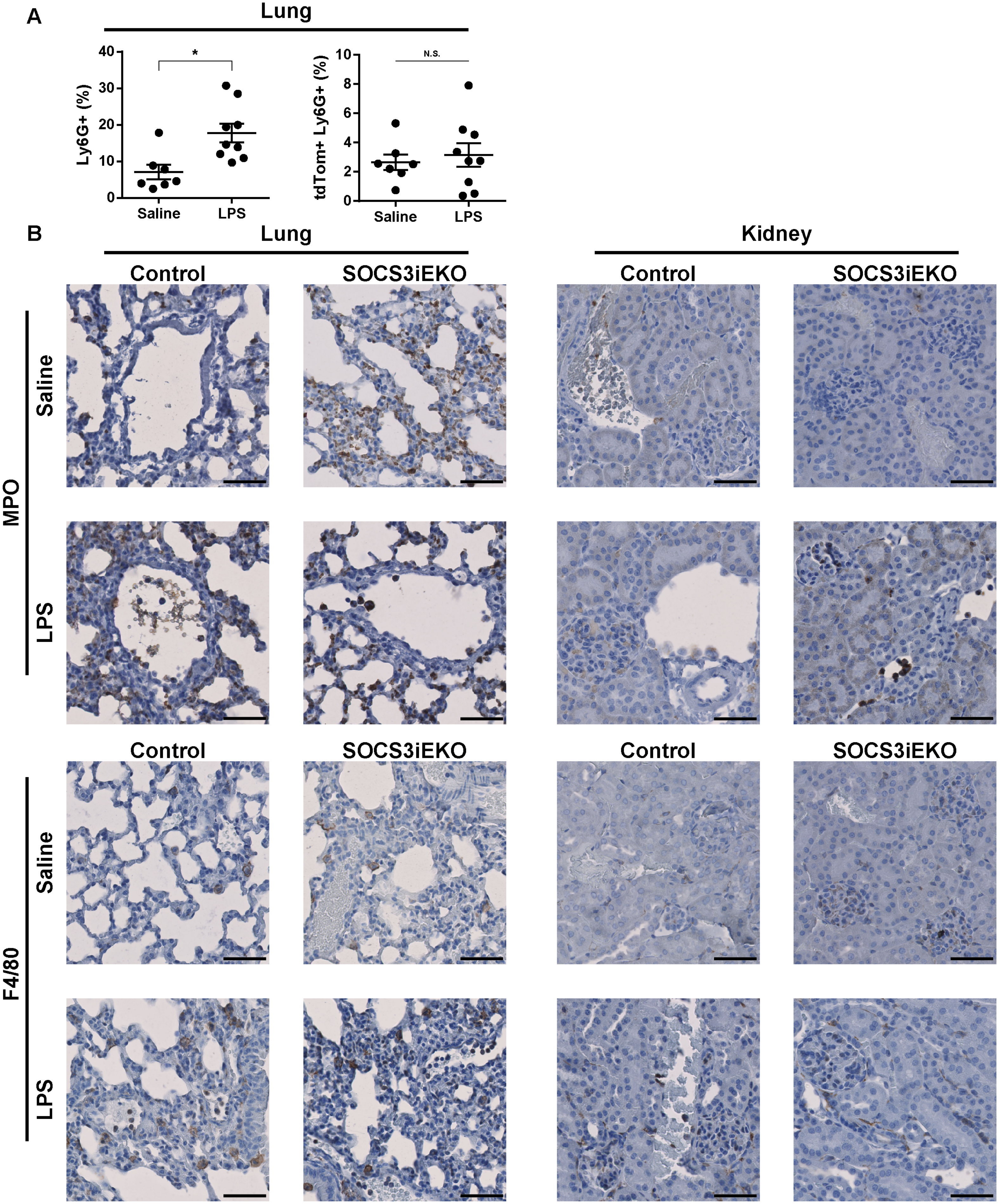

Supplemental Figure 6. Neutrophil and monocyte infiltration in endotoxemic organs. A, flow cytometry analysis of Ly6G+ cells in lungs. Asterisks denote $p < 0.05$ (Two-tailed T-test). Data from two independent experiments (n=7 saline- and n=9 LPS-treated mice). B, Immunohistochemical staining of FFPE sections for myeloperoxidase (MPO, a neutrophil marker) and F4/80 (a monocyte marker) in lungs and kidneys. Bars, 50 μm. Images are representative from three independent experiments.

Supplemental Table 1

M&M section	Reagent or disposable	Vendor	Catalog Number
**Mice**	Lipopolysaccharides from *Escherichia coli* O111:B4	Millipore Sigma	L4391
	B6.Gt(ROSA)26Sor$^{tm9(CAG-tdTomato)Hze}$	The Jackson Laboratory	07909
	B6;129S4-Socs3^{tm1Ayos}/J	The Jackson Laboratory	010944
	Goldenrod 5 mm lancet	Medipoint	5mm
	EDTA Vacutainer tube	BD	367836
	Lithium Heparin Capillary Blood Collection Tube	Sarstedt	20.1292.100
**Blood measurements**	Element POC Test Card with BUN	Heska	5424
	IL-6 ELISA	Proteintech	KE10007
	TNF-α ELISA	Proteintech	KE10002
	BCP Albumin Assay Kit	Abcam	AB272526
	Mouse serum albumin	Millipore Sigma	A3139
**Glomerular Filtration Rate**	Transdermal Mini GFR Monitor	MediBeacon	TDM-MD004
	FITC-Sinistrin	MediBeacon	FTC-FS001
**Assessment of Vascular Leak**	FITC-labeled 70 kDa dextran	Invitrogen	D1822
	Alexa Fluor 647-labeled 10 kDa dextran	Invitrogen	D22914
	sucrose	VWR	97061-426
	Polyvinyl-pyrrolidone (avg MW 40 kDa)	Millipore Sigma	PVP-40
	Ethylene Glycol	VWR	97061-964
	Tissue-Tek optimum cutting temperature	Sakura Finetek	4583
	DAPI	Invitrogen	D3571
	Fluoroshield with 1,4-Diazabicyclo [2.2.2] octane	Millipore Sigma	F937
**Histology and Immunofluorescence**	Harris Hematoxylin	Millipore Sigma	HHS16
	Eosin Y	Richard Allen	7111
	Periodic Acid-Schiff (PAS) Kit	Millipore Sigma	395B
	Gill No. 3 Hematoxylin	Millipore Sigma	GHS332
	Triton X-100	Millipore Sigma	T8787
	Fetal bovine serum	Millipore Sigma	12306C
	BOVINE SERUM ALBUMIN - Fraction V (Immunoglobulin and Protease Free)	Rockland	BSA-50
**Cell culture**	collagenase	Worthington	LS004196
	phenol red-free EBM 2 media	PromoCell	C-22211
	EGM-2 Growth Medium 2 Supplement Mix	PromoCell	C-39216
	Trypsin-EDTA Solution 1X	Millipore Sigma	54917C-100ML
	Penicillin-Streptomycin Solution, 100x	Corning	30-002-CI-PK

	Antibiotic Antimycotic Solution (100×), Stabilized	Millipore Sigma	A5955-20ML
	FITC-Ulex europaeus lectin	Millipore Sigma	L9006
	gelatin	Millipore Sigma	ES-006-B
	Ruxolitinib	Selleck Chem	S1378
	MG132	Calbiochem	474790
	Cycloheximide	Acros Organics	357420010
	DMSO	Millipore Sigma	472301
	Recombinant human IL-6	R&D Systems	206-IL-050
	Recombinant human sIL-6Rα	R&D Systems	227-SR-025/CF
**Lentiviral delivery**	SOCS3 (NM_003955) Human Tagged ORF Clone	Origene	RC209305
	pLenti-C-Myc-DDK-P2A-tGFP Lentiviral Gene Expression Vector	Origene	PS100088
	PfuUltra II Fusion High-fidelity DNA Polymerase	Agilent	600670
	HEK293FT	Invitrogen	R70007
	pCMV-dR8.2 dvpr	Addgene	8455
	pCMV-VSVG	Addgene	8454
	Vivacell 100, 30,000 MWCO PES, 10pc	Sartorius	VC1022
**Measurement of monolayer permeability**	8-well electrode array	Applied Biophysics	8W10E PET
	96-well electrode array	Applied Biophysics	96W10idf PET
**Gene knockdown**	ON-TARGETplus set of 4 SOCS3 siRNA	Dharmacon	LQ-004299-00-0005
	ON-TARGETplus non-targeting control pool	Dharmacon	D-001810-10-20
	Opti-MEM	Thermo Scientific	31985070
	lipofectamine RNAiMAX transfection reagent	Invitrogen	13778150
**Gel Electrophoresis and Immunoblotting**	complete protease inhibitor mixture	Roche Applied Science	11697498001
	PhosSTOP phosphatase inhibitor mixture	Roche Applied Science	4906845001
	Sodium fluoride	Millipore Sigma	S-1504
	phenyl arsine oxide	Millipore Sigma	P-3075
	Sodium pyrophosphate decahydrate	Millipore Sigma	221368-100G
	Sodium orthovanadate	Millipore Sigma	S6508
	Transblot Turbo RTA Mini Nitrocellulose Transfer kit	Bio-Rad	1704270
	Clarity Western ECL Substrate	Bio-Rad	1705061
	Clarity Max Western ECL Substrate	Bio-Rad	1705062
	SuperSignal West Femto Maximum Sensitivity Substrate	Pierce	34094

**RNA isolation and RT-qPCR**	1.0 mm dia zirconia/silica beads	Biospec	11079110z
	TRIzol reagent	Invitrogen	15596018
	PrimeScript RT Master Mix	Takara Bio	RR036B
	iTaq Universal SYBR Green Supermix	Bio-Rad	1725125
**Flow cytometry**	Collagenase type I	Worthington	LS004197
	Dispase II	Roche Applied Science	10888700
	DNAse I	Roche Applied Science	10104159001
	EDTA Capillary Blood Collection Tube	Sarstedt	20.1288.100
	Pacific blue Annexin V Apoptosis Detection kit with 7-AAD	Biolegend	640926
	RBC lysis buffer	Roche Applied Science	11814389001
**ELISA**	IFNα kit	Thermo Scientific	BMS216
	IFNβ kit	PBL Assay Science	41410
	IFNγ kit	Thermo Scientific	EHIFNG
**Immunohistochemistry**	VECTASTAIN Elite ABC-HRP Kit	Vector Labs	PK-6100
	ImmPACT® DAB Substrate, Peroxidase	Vector Labs	SK-4105
	VectaMount Permanent Mounting Medium	Vector Labs	H-5000
**Isolation of RNA from spleen CD45+ cells**	Dynabeads M-280 Streptavidin	Invitrogen	11205D
	RNeasy Plus Micro Kit	Qiagen	74034
	Falcon 70 µm Cell Strainer	Corning	352350

Supplemental Table 2
List of antibodies

Target	Species	Clone or isotype	Conjugate	Vendor	Catalog No	RRID
**STAT3**	rabbit	clone D1A5		Cell Signaling Technology	8768S	AB_2722529
**pY705-STAT3**	rabbit	clone D3A7		Cell Signaling Technology	9145L	AB_2491009
**SOCS3**	rabbit	polyclonal IgG		Abcam	AB16030	AB_443287
**E-selectin**	rabbit	polyclonal IgG		Abcam	AB18981	AB_470289
**P-selectin**	goat	polyclonal IgG		R&D Systems	AF737	AB_2285644
**CD45**	mouse	clone 30-F11	FITC	BioLegend	103107	AB_312972
**CD45**	mouse	clone 30-F11	Biotin	BioLegend	103104	AB_312969
**Histone H3 (citrulline R2 + R8 + R17)**	rabbit	polyclonal IgG		Abcam	AB5103	AB_304752
**DDK**	mouse	clone OTI4C5		Origene	TA50011100	AB_2622345
**Turbo GFP**	rabbit	polyclonal IgG		Fujifilm Wako Chemicals	AB513	
**β-actin**	mouse	clone AC-15		Millipore Sigma	A5441	AB_476744
**α-smooth muscle actin**	mouse	clone 1A4		Millipore Sigma	A2547	AB_476701
**VE-cadherin**	goat	polyclonal		R&D Systems	AF938	AB_355726
**Myeloperoxidase**	rabbit	clone EPR20257		Abcam	ab208670	AB_2864724
**F4/80**	rabbit	clone D2S9R		Cell Signaling	70076S	AB_2799771
**Ly6G**	rat	clone 1A8	APC/Cy7	BioLegend	127624	AB_10640819
**mouse IgG**	goat	polyclonal	HRP	Jackson Immunoresearch	115-035-062	AB_2338504
**rabbit IgG**	goat	polyclonal	HRP	Jackson Immunoresearch	111-035-003	AB_2313567
**goat IgG**	bovine	polyclonal	HRP	Jackson Immunoresearch	805-035-180	AB_2340874
**mouse IgG**	donkey	polyclonal	Alexa Fluor 647	Invitrogen	A-31571	AB_162542
**rabbit IgG**	donkey	polyclonal	Alexa Fluor 647	Invitrogen	A-31573	AB_2536183
**goat IgG**	donkey	polyclonal	Alexa Fluor 647	Invitrogen	A-21447	AB_2535864
**rabbit IgG**	goat	polyclonal	Biotin	Vector Labs	BA-1000	AB_2313606

Supplemental Table 3
Mouse RT-qPCR primers (SYBR green)

Gene symbol	Forward	Reverse
**HPRT**	TGGCCCTCTGTGTGCTCAA	TGATCATTACAGTAGCTCTTCAGTCTGA
**C5AR**	CCATTAGTGCCGACCGTTTCCT	CACGAAGGATGGAATGGTGAGG
**CD44**	CGGAACCACAGCCTCCTTTCAA	TGCCATCCGTTCTGAAACCACG
**CEMIP**	CTCTACACAGGTCAAGGTGGCA	CCATCACCACAATGTTCCGAGTC
**CLU**	GATGATCCACCAGGCTCAACAG	ACACAGTGCGGTCATCTTCACC
**CXCL2**	CATCCAGAGCTTGAGTGTGACG	GGCTTCAGGGTCAAGGCAAACT
**CXCL9**	CCTAGTGATAAGGAATGCACGATG	CTAGGCAGGTTTGATCTCCGTTC
**DDX58**	AGCCAAGGATGTCTCCGAGGAA	ACACTGAGCACGCTTTGTGGAC
**ET1**	CTACTTCTGCCACCTGGACATC	CGCACTGACATCTAACTGCCTG
**F3**	GCACCGAGCAATGGAAGAGTTTC	CTTTCTGTCCCGCTCGGTTCTT
**FOS**	GGGAATGGTGAAGACCGTGTCA	GCAGCCATCTTATTCCGTTCCC
**HAVCR1**	CTGGAATGGCACTGTGACATCC	GCAGATGCCAACATAGAAGCCC
**ICAM**	AAACCAGACCCTGGAACTGCAC	GCCTGGCATTTCAGAGTCTGCT
**HNF1**	AGAGACCTTGGTGGAGGAGTGT	GGCAAACCAGTTGTAGACACGC
**IFIH1**	TGCGGAAGTTGGAGTCAAAGCG	CACCGTCGTAGCGATAAGCAGA
**IL18**	GACAGCCTGTGTTCGAGGATATG	TGTTCTTACAGGAGAGGGTAGAC
**IL6**	TACCACTTCACAAGTCGGAGGC	CTGCAAGTGCATCATCGTTGTTC
**IRF1**	TCCAAGTCCAGCCGAGACACTA	ACTGCTGTGGTCATCAGGTAGG
**IRF7**	CCTCTGCTTTCTAGTGATGCCG	CGTAAACACGGTCTTGCTCCTG
**IRF8**	AGGTCTTCGACACCAGCCAGTT	GCACGAGAATGAGTTTGGAGCG
**KLF2**	CGCCGCCACACATACTTG	AACTTCCAGCCGCATCCTT
**LCN2**	ATGTCACCTCCATCCTGGTCAG	GCCACTTGCACATTGTAGCTCTG
**MX1**	TGGACATTGCTACCACAGAGGC	TTGCCTTCAGCACCTCTGTCCA
**OAS2**	CACCAAAGTCCTGAAGACCGTC	AGAGTCGTAACTCTCCAGCGAG
**OAS3**	TTCTCTGCCAGCTTCGGAAAGC	CTCTGAAGGCAGACTTGTGACC
**OASL1**	TGAAGAGCCTCCTTCGGTTGGT	TCCAGCCTGAAGTTGGCATCCT
**OASL2**	CCAAAACGAGGTCGTCAGGAAC	AGCCACCTGTTCCCATCCCTTT
**PLAT**	GTTACACAGCGTGGAGGACCAA	CACGTCAGCTTTCGGTCCTTCA
**PLAU**	AGAAGCGACCCTGGTGCTATGT	CCACACTGGAAGCCTTGTTGGT
**PLAUR**	AGGACTACCGTGCTTCGGGAAT	ACACGGTCTCTGTCAGGCTGAT
**PROCR**	CCTCCAAAGCAGCCAACTTCAC	GTGTAAGAGCGACCTGTTTGGC
**PTGS2**	GCGACATACTCAAGCAGGAGCA	AGTGGTAACCGCTCAGGTGTTG
**RHOU**	TGGTCAGCTACACCACTAACGG	TGCAGTGTCACAGAGCTGGAGT
**SELE**	GGACACCACAAATCCCAGTCTG	TCGCAGGAGAACTCACAACTGG
**SELP**	AAGATGCCTGGCTACTGGACAC	CAAGAGGCTGAACGCAGGTCAT
**SERPINE1**	CCTCTTCCACAAGTCTGATGGC	GCAGTTCCACAACGTCATACTCG
**SOCS3**	GGACCAAGAACCTACGCATCCA	CACCAGCTTGAGTACACAGTCG
**STAT2**	GAACCAACTCTCCATTGCCTGG	CGTAAGAGGAGAACTGCCAGCT
**STAT3**	AGGAGTCTAACAACGGCAGCCT	GTGGTACACCTCAGTCTCGAAG
**SYNPO**	TCCTTCTCCACCCGGAATGCTG	AGCCGTCCAGGCTGCTAGGAG
**TFPIA**	CTGTGAGAATCCAGTCCACTCC	GCCACGATAATCCCGACG
**TFPIB**	CTGTGAGAATCCAGTCCACTCC	TCTTCTTTTGTAACCCGACGC
**TFPIC**	CTGTGAGAATCCAGTCCACTCC	TACCAAGGCAGCCCGAC
**THBD**	GGAGAATGGTGGCTGTGAGTAC	GCACGATTGAACCACAGGTCTTG

**TSP1**	TGGCCAGCGTTGCCA	TCTGCAGCACCCCCTGAA
**VEGFA**	CTGCTGTAACGATGAAGCCCTG	GCTGTAGGAAGCTCATCTCTCC
**VWF**	AACAGACGATGGTGGACTCAGC	CGATGGACTCACAGGAGCAAGT
**PTPRC**	CTTCAGTGGTCCCATTGTGGTG	TCAGACACCTCTGTCGCCTTAG

Human RT-qPCR primers (SYBR green)

**Gene symbol**	**Forward**	**Reverse**
**GAPDH**	GTCTCCTCTGACTTCAACAGCG	ACCACCCTGTTGCTGTAGCCAA
**SOCS3**	CATCTCTGTCGGAAGACCGTCA	GCATCGTACTGGTCCAGGAACT
**IL6**	AGACAGCCACTCACCTCTTCAG	TTCTGCCAGTGCCTCTTTGCTG
**CXCL2**	GGCAGAAAGCTTGTCTCAACCC	CTCCTTCAGGAACAGCCACCAA
**RHOU**	ACTGCCTTCGACAACTTCTCCG	GAGCAGGAAGATGTCTGTGTTGG
**PTGS2**	CGGTGAAACTCTGGCTAGACAG	GCAAACCGTAGATGCTCAGGGA
**IFNA2**	TGGGCTGTGATCTGCCTCAAAC	CAGCCTTTTGGAACTGGTTGCC
**IFNA17**	GAAGACTCAAGCCATCTCTGTCC	TAGGAGGCTCTGTTCCCAAGCA
**IFNB**	CTTGGATTCCTACAAAGAAGCAGC	TCCTCCTTCTGGAACTGCTGCA
**MX1**	GGCTGTTTACCAGACTCCGACA	CACAAAGCCTGGCAGCTCTCTA
**IRF1**	GAGGAGGTGAAAGACCAGAGCA	TAGCATCTCGGCTGGACTTCGA
**IRF7**	CCACGCTATACCATCTACCTGG	GCTGCTATCCAGGGAAGACACA
**ADAMTS1**	GCGTCAATGCTTTCCAACCTGG	GGGATTCTGAGGCTTGTCCATC
**ADAMTS4**	TCACTGACTTCCTGGACAATGGC	GGTCAGCATCATAGTCCTTGCC
**ADAMTS9**	CCATTCAGAGGTGCAGTGAGTTC	ACCAGACCTGGCGGTGCTTATG
**TMEM2**	GGAATAGGACTGACCTTTGCCAG	TTCTGACCACCCTGAAAGCCGT
**CEMIP**	ACCGAGCACATTCCAACTACCG	GGCAGAGATGATTGAGAGGAACG
**ARID5A**	TGGCAAGCAGAACGGAATCCAG	CTTGTAGAGGCTGACCAGGAAG
**CNKSR3**	AAAACCTACGGTGGAAGCCACC	ATCCAGGATGGCTGACTTCTCC
**EPSTI**	ACTGAAACGGCAGCAGCAAGAG	TCCAACAGCCTCCAGATTGCTC
**GNA14**	AAGCAGCTCTGGCAAGATCCAG	GTAGGCACGAATGATGGTGTGG
**KLF4**	ACCTACACAAAGAGTTCCCATC	TGTGTTTACGGTAGTGCCTG
**PTPN13**	GGATGAAGCCACTTACTCCAGC	CTCCAGGCTTAGGAGGTGATGA
**BATF3**	ACCGAGTTGCTGCTCAGAGAAG	AGGTGCTTCAGCTCCTCTGTCA
**SBNO2**	GAGAACGATGGGCACCTCAACT	TGTCCCGCTTTCTCTTGGTGGA

Supplemental Table 4
Primers for genotyping

		**Male**	**Female**
**SRY**		**273 bp**	**-**
Fwd	TTG TCT AGA GAG CAT GGA GGG CCA TGT CAA		
Rev	CCA CTC CTC TGT GAC ACT TTA GCC CTC CGA		

		**WT**	**Flox**
**SOCS3 flox**		**52 bp**	**181 bp**
Fwd	CGG GCA GGG GAA GAG ACT GT		
Rev	TCG ACT GTC CTC GGT CAC		

		**WT**	**Tom**
**Rosa26 locus**		**297 bp**	**196 bp**
Wild type Fwd	AAG GGA GCT GCA GTG GAG TA		
Wild type Rev	CCG AAA ATC TGT GGG AAG TC		
Mutant Fwd	CTG TTC CTG TAC GGC ATG G		
Mutant Rev	GGC ATT AAA GCA GCG TAT CC		

		**Present**	**Deleted**
**SOCS3 Deletion**		**>1400 bp**	**424 bp**
Fwd	GCG GGC AGG GGA AGA GAC TGT CTG GGG TTG		
Rev	CCT TTT CTC TTC CAT CCT TCC		

		**WT**	**Cre**
**Cre**		**-**	**199 bp**
Fwd	ACC AGC CAG CTA TCA ACT CG		
Rev	TTA CAT TGG TCC AGC CAC C		

Supplemental Table 5

Score	0	1	2	3
**Appearance**	Smooth coat	Slightly roughed fur	Majority of fur on back is ruffled	Piloerection, puffy appearance
**Consciousness**	Active	Active, avoids standing upright	Active only when provoked	Non-responsive, even when provoked
**Activity**	Normal	Suppressed eating, drinking, or running	Stationary	Stationary, even when provoked
**Eyes**	Open	Not fully open, potential secretions	Half closed, potential secretions	No response to touch stimulus
**Posture**	Normal	Hunched, moving freely	Hunched, strained or stiff movement	Hunched, little or no movement

Supplemental Table 6

Mouse ID	Gender	Genotype	Tx	Group	Pre-TX Wei	Pre-TX Ten	Time post-(	Post-TX We
5129	F	fl/+	Saline	Saline_fl/+	21.2	37.5	16.5	21
5130	F	fl/fl	Saline	Saline_fl/fl	21.3	37.7	16.5	21.2
5132	M	fl/fl	Saline	Saline_fl/fl	19.2	37.4	16.5	18.1
5133	F	fl/fl	Saline	Saline_fl/fl	20.4	37.2	16.5	19.1
5135	M	fl/fl	Saline	Saline_fl/fl	21.9	37.9	16.5	21
5137	M	fl/fl	LPS	LPS_fl/fl	21.6	37.9	16.5	19.8
5139	M	+/+	Saline	Saline_+/+	23.2	37.2	16.5	22.4
5140	F	+/+	LPS	LPS_+/+	20.7	38.1	16.5	18.3
5142	F	fl/+	Saline	Saline_fl/+	17.3	38.1	16.5	16.7
5143	F	fl/+	LPS	LPS_fl/+	21.2	37.6	16.5	19.2
5144	M	fl/+	LPS	LPS_fl/+	24.9	37.9	16.5	22.9
5150	M	fl/fl	Saline	Saline_fl/fl	25.3	37.6	14.5	25.6
5151	F	fl/fl	Saline	Saline_fl/fl	19.9	37.4	14.5	19.4
5155	F	fl/fl	LPS	LPS_fl/fl	20.8	37.8	14.5	19.7
5156	F	fl/fl	LPS	LPS_fl/fl	22.4	37.8	14.5	19.9
5178	M	fl/fl	LPS	LPS_fl/fl	22.7	37.3	14.5	21.7
5179	M	fl/fl	Saline	Saline_fl/fl	24.4	37.2	14.5	24.9
5180	F	+/+	LPS	LPS_+/+	19.4	38.1	14.5	17.9
5181	M	fl/+	LPS	LPS_fl/+	22.5	37.1	14.5	21.9
5182	F	fl/fl	Saline	Saline_fl/fl	21.2	37.6	14.5	19.2
5183	F	+/+	Saline	Saline_+/+	19.8	38	14.5	21
5184	M	fl/+	Saline	Saline_fl/+	25.7	37.8	14.5	26.9
5185	M	fl/+	LPS	LPS_fl/+	26.1	37.8	14.5	23.9
5219	M	+/+	LPS	LPS_+/+	27.1	37.5	14.5	24.2
5220	M	fl/+	LPS	LPS_fl/+	28.2	37	14.5	26.5
5221	M	fl/+	saline	Saline_fl/+	25.4	37.2	14.5	25.6
5226	M	+/+	saline	Saline_+/+	25.1	37.7	14.5	25.4
5228	M	fl/+	LPS	LPS_fl/+	25.3	37.6	14.5	23
5230	F	+/+	LPS	LPS_+/+	20.2	37.5	14.5	18.5
5231	F	fl/+	LPS	LPS_fl/+	19.4	37.3	14.5	18.3
5232	F	fl/fl	LPS	LPS_fl/fl	20.7	37.9	14.5	19.6
5233	M	fl/+	LPS	LPS_fl/+	26.2	36.9	14.5	25
5234	M	+/+	LPS	LPS_+/+	28.1	36.9	14.5	26.1
5239	F	fl/+	LPS	LPS_fl/+	21.2	37.3	14.5	19.3
5240	F	fl/+	saline	Saline_fl/+	19.8	37.6	14.5	20
5241	M	fl/+	saline	Saline_fl/+	26.6	37.2	14.5	27.1
5242	M	fl/fl	LPS	LPS_fl/fl	22.9	37.6	14.5	21.3
5243	M	+/+	LPS	LPS_+/+	28.3	37.8	14.5	26.1
5244	M	+/+	LPS	LPS_+/+	28.4	37	14.5	26
5245	F	+/+	saline	Saline_+/+	20.8	37.4	14.5	21.2

Post-TX Te	Delta Weig	Delta Temp	Appearanc	Consciousn	Activity	Eyes	Posture	Score
38.2	-0.2	0.7	0	0	0	0	0	0
37.9	-0.1	0.2	0	0	0	0	0	0
38.4	-1.1	1	0	0	0	1	0	1
38.1	-1.3	0.9	0	0	0	0	0	0
37.9	-0.9	0.1	0	0	0	0	0	0
35.7	-1.8	-2.2	1	2	2	1	2	8
38.1	-0.8	0.8	0	0	0	0	0	0
36.2	-2.4	-1.9	1	1	0	2	1	5
38.2	-0.6	0.1	0	0	0	0	0	0
34.4	-2	-3.2	1	1	1	2	2	7
37.1	-2	-0.9	1	0	0	1	1	3
37.6	0.3	0	0	0	0	0	0	0
37.7	-0.5	0.3	0	0	0	0	0	0
33.9	-1.1	-3.9	2	3	3	0	3	11
37.2	-2.5	-0.6	0	0	1	1	0	2
33.9	-1	-3.4	1	3	3	2	3	12
37.7	0.5	0.4	0	0	0	0	0	0
36.3	-1.5	-1.8	1	2	2	2	1	8
37.8	-0.6	0.7	0	0	0	0	0	0
37.7	-2	0.2	0	0	0	0	0	0
37.6	1.2	-0.4	0	0	0	0	0	0
38.1	1.2	0.2	0	0	0	0	0	0
36.2	-2.2	-1.6	1	1	1	1	1	5
37.2	-2.9	-0.3	0	1	1	1	0	3
36.8	-1.7	-0.2	1	1	1	1	1	5
38.1	0.2	0.9	0	0	0	0	0	0
37.8	0.3	0.1	0	0	0	0	0	0
36.9	-2.3	-0.7	1	1	1	2	1	6
35.8	-1.7	-1.7	1	1	2	1	3	8
34.6	-1.1	-2.7	1	2	3	2	2	10
33.9	-1.1	-4	2	3	3	1	3	12
36.4	-1.2	-0.5	2	1	1	2	1	7
36.8	-2	-0.2	1	0	1	1	1	4
35.2	-1.9	-2.1	1	1	2	2	2	8
38	0.2	0.4	0	0	0	0	0	0
38	0.5	0.8	0	0	0	0	0	0
34.5	-1.6	-3.1	2	3	3	2	3	13
37.3	-2.2	-0.4	1	0	0	0	1	2
37.4	-2.4	0.4	1	0	0	1	1	3
37.9	0.4	0.5	0	0	0	0	0	0

WBC	NEU	LYM	MONO	EOS	BAS	RBC	HGB	PLT
7.53	1.08	6.33	0.04	0.05	0.03	10.22	15.7	1013
8.21	3.47	4.41	0.18	0.06	0.09	8.87	14.6	1038
3.19	1.2	1.92	0.04	0.02	0.01	9.6	15	1044
3.98	0.72	3.12	0.04	0.09	0.01	8.17	13.3	659
6.78	1.29	5.34	0.08	0.05	0.02	10.27	15.7	1197
5.17	3.8	0.76	0.34	0.2	0.07	8.76	13.5	559
5.95	1.21	4.61	0.08	0.03	0.02	10.8	16.3	957
2.92	1.77	0.8	0.19	0.12	0.04	8.33	13	576
6.07	1.24	4.61	0.12	0.06	0.04	10.08	15.7	1213
2.63	1.32	1.08	0.15	0.06	0.02	8.8	13.2	399
1.85	1.45	0.25	0.06	0.08	0.01	8.93	13.8	473
4.73	0.93	3.59	0.12	0.08	0.01	9.38	14.6	868
4.11	1.2	2.77	0.06	0.05	0.03	9.63	15.5	1146
2.35	1.43	0.61	0.22	0.07	0.02	9.03	15.1	658
1.71	1.12	0.38	0.15	0.05	0.01	8.86	14.4	374
3.62	2.2	0.7	0.49	0.19	0.04	8.2	13	377
5.34	0.82	4.41	0.07	0.03	0.01	9.46	14.7	1027
1.58	0.97	0.49	0.09	0.02	0.01	8.94	14.6	377
3.17	0.86	2.2	0.08	0.02	0.01	9.28	15.2	834
5.93	3.5	2.12	0.2	0.06	0.05	8.69	14.2	776
5.92	1.16	4.59	0.07	0.07	0.03	9.47	15.2	1353
5.1	1.22	3.63	0.13	0.11	0.01	9.24	14.5	1344
3.49	2.55	0.66	0.17	0.09	0.02	8.62	13.6	612
3.3	2.57	0.47	0.16	0.06	0.04	8.62	13.8	527
3.76	2.82	0.75	0.11	0.06	0.02	8.46	13.5	645
10.6	2.61	7.34	0.28	0.3	0.07	8.92	14.3	1453
6.64	2.22	4.02	0.23	0.12	0.05	9.12	14.4	1397
0.35	0.24	0.07	0.03	0.01	0	1.05	1.7	48
2.12	1.3	0.62	0.09	0.08	0.03	8.4	13.8	287
4.28	2.99	1.03	0.16	0.08	0.02	8.02	12.7	671
6.45	3.4	1.93	0.57	0.43	0.12	10.98	17.8	443
18.05	13.04	1.83	2.18	0.66	0.34	8.23	13.4	674
2.61	1.57	0.8	0.14	0.07	0.03	8.44	13.8	630
2.15	0.93	1	0.13	0.07	0.02	8.39	13.5	351
8.27	1.81	5.8	0.32	0.18	0.16	8.86	14.8	1111
6.19	1.4	4.37	0.2	0.18	0.04	8.77	14.7	684
4.59	3.31	0.78	0.27	0.16	0.07	9.24	15	538
2.73	1.89	0.44	0.18	0.16	0.06	8.08	13.4	436
3.21	2.43	0.45	0.16	0.13	0.04	8.16	13.7	333
12.34	1.94	9.8	0.25	0.28	0.07	8.69	14.7	1357

Plasma IL-6	Plasma TNF	pH	Sodium	Potassium	Chloride	Calcium, io	Lactate	BUN
50.28	165.09	7.266	143	5.8	123	0.78	8.84	31
0	145.56	7.121	146	4.8	114	1.2	9.97	24
19.17	177.96	7.298	143	5.7	112	0.81	8.01	30
1.53	125.09	7.155	139	5.3	114	1.01	8.57	38
21.39	188.8	7.354	140	7.3	115	0.81	3.11	49
14614.72	412.87	7.193	141	7.7	123	0.63	8.22	115
57.78	146.76	7.119	140	6.3	114	0.84	8.65	32
1915.83	462.87							
74.72	145.46	6.888	143	5.6	115	1.08	14.25	35
15394.44	1098.75							
901.11	361.57	7.367	144	6.5	123	0.7	5.26	72
67.78	176.67	7.273	147	7.6	120	0.76	8.59	29
62.22	414.75	7.448	141	5.9	117	0.54	7.97	31
16021.39	249.72	7.311	138	10.7	123	0.53	10.69	103
273.61	205.09	7.235	147	7.6	121	1.05	8.09	58
19471.39	591.76							
58.61	166.02	7.085	144	4.8	116	1.04	11.1	26
4780.56	242.22							
19.03	179.91							
177.22	103.8	7.305	150	6.3	116	0.98	8.48	59
44.17	97.59	6.969	148	4.8	117	1.11	14.39	25
26.67	162.96	7.221	147	6.2	122	0.8	9.74	31
2417.78	302.69	7.159	147	5.2	115	1.02	7.65	81
1017.5	346.2	7.3	148	5.3	115	1.03	4.43	56
2418.89	330.56	7.28	148	5	119	1	3.94	87
49.17	227.04	7.266	147	4.5	114	1	7.04	29
34.17	163.06	7.281	147	4.7	113	1.06	6.44	25
976.39	210.37	7.342	147	6.1	124	0.84	3.6	70
2265.83	319.81	7.321	149	6.3	122	0.93	4.22	89
15201.94	375.28	7.272	146	5.5	120	0.82	5.57	112
19066.67	908.33							
5830.83	296.67	7.251	150	6.1	118	1.03	5.52	120
1151.94	254.17	7.305	149	4.8	116	1.12	2.7	73
9171.39	308.52	7.35	143	6.6	124	0.59	4.23	120
212.22	207.41							
80	121.67	7.291	148	4.5	114	1.11	5.99	26
19425.56	365.93							
623.89	194.26	7.281	146	8.4	120	0.94	3.34	66
1295.83	237.69	7.309	148	6.5	128	0.81	3.81	76
383.33	245.93	7.212	148	4.3	116	1.05	8.6	23

Creatinine	Glucose	Comments	KIDNEY-ICA	Kidney_SEL	Kidney_IL6	Kidney_SO	Kidney_STA	Kidney_CEI
0.54	323		0.974237	2.852996	0.429458	0.917319	1.331688	4.059992
0.51	285		1.208472	9.704373	0.970508	1.567294	1.441893	3.798868
0.65	290		0.777296	2.334025	1.001053	1.499879	1.354241	3.372349
0.63	367		1.39187	12.26424	1.208547	2.178168	1.523736	3.906482
0.61	351		1.50124	3.955029	1.790699	2.391218	1.510737	2.534373
1.23	42		4.593022	59.14627	165.4945	41.83384	5.45406	6.947351
0.61	334		1	1	1	1	1	1
			8.689115	39.91817	45.61319	27.0153	3.591692	9.752334
0.71	367		1.022538	1.98099	1.128824	1.765676	1.554816	3.98246
			10.62946	61.36169	243.0559	76.68324	6.911494	14.55335
0.86	47		8.303351	35.71454	36.39814	19.79696	3.283171	4.846128
0.74	253		1.417461	0.890607	1.141455	1.187038	0.660242	0.267909
0.86	134		1.352659	5.277981	0.196408	1.817712	0.003825	0.018837
1.39	89		46.60823	12.60698	1264.124	61.87883	5.80375	2.150206
0.69	116	No respons	2.274288	2.505605	70.36199	1.060961	0.432337	0.167342
			13.04893	0.264578	0.045541	12.51965	4.08E-05	
0.58	314		1.179205	0.567816	0.706134	0.63405	0.455436	0.39091
			17.22841	5.541168	307.2423	46.08009	3.170833	2.70292
			1.65539	0.319231	1.398057	0.498688	0.586179	0.410729
0.87	90	Ascites and	3.129167	3.674782	40.7873	5.060939	1.78649	11.2968
0.52	336		1	1	1	1	1	1
0.79	261		0.846414	0.400316	1.535373	0.657658	0.412856	0.341662
0.92	87		12.59868	4.021119	111.9814	18.37697	2.026462	0.986205
0.88	80		3.966549	1.339715	71.51308	4.92946	3.128638	1.436778
0.93	72		7.577332	0.437029	19.55778	12.6963	1.999775	0.710962
0.54	343		0.607498	0.641538	0.251707	0.287852	1.539597	0.115715
0.46	283		1.125886	2.324485	2.259068	0.445606	0.683977	0.643742
0.91	61		5.337212	1.132363	15.35605	2.512897	9.780663	1.704734
1.24	64		8.05127	6.065337	215.4659	10.42297	3.190871	12.3662
1.17	73		12.23406	3.591231	246.6908	47.63027	8.080584	1.829973
			26.55998	1.913857	2246.9	32.4628	6.215235	1.909086
1.21	89		3.439425	11.13132	231.4011	93.47093	2.991525	2.188826
0.82	73		4.526962	0.385928	14.42871	19.13539	7.818978	0.670756
1.35	60		7.593538	3.030757	174.2235	14.37975	4.448839	7.93033
			0.535226	1.064961	0.434314	1.57377	0.985239	3.859571
0.4	297		0.365172	0.455991	0.842696	0.600299	0.658247	0.286764
			9.008218	14.41105	534.9411	160.5271	5.981587	2.318516
1.64	94		3.599245	1.436857	25.26896	2.671797	3.418219	0.480678
0.88	70		10.5264	1.34671	16.05367	19.77755	2.973867	1.812258
0.51	267		0.88819	0.430203	0.44266	2.244137	1.462037	1.553416

Kidney_F3	Kidney_RH	Kidney_TSI	Kidney_PL/	Kidney_PL/	Kidney_THI	Kidney_PL/	Kidney_SYI	Kidney_ET1
1.057323	1.258691	1.217768	2.823779	0.649582	1.28054	1.202053	0.921469	2.141539
1.139266	1.016983	0.666185	1.850634	0.927991	1.110789	1.173216	3.88909	0.00345
2.356474	1.35769	1.881335	1.604092	0.898269	1.23302	2.066932	1.088149	1.60886
1.087614	1.230023	1.428342	2.283677	1.240926	1.570563	1.923369	2.118398	1.812708
1.933402	1.876746	1.801265	1.93075	0.956443	1.37209	2.786103	1.266815	1.235826
1.616027	3.104751	0.392981	1.69354	0.347262	1.180555	14.12524	0.536997	5.097314
1	1	1	1	1	1	1	1	1
0.40286	2.763151	0.497363	2.888864	0.114009	0.656635	7.658922	0.598956	4.919259
0.637245	1.597086	1.248829	1.698231	1.083827	1.003369	1.441698	1.545709	1.283116
1.163839	5.931259	0.499789	2.82787	0.097415	1.278814	16.42584	0.363401	9.970851
0.828219	1.964488	0.465636	1.665712	0.215573	0.819921	4.156163	0.596082	2.99914
1.713227	0.440546	0.64636	0.4145	0.581444	0.625248	0.982766	0.426872	0.8357
0.077925	0.031252	0.098548	0.959995	0.565425	1.454527	2.466106	1.441182	5.481573
12.25846	3.598869	0.626026	1.513406	0.136103	0.961505	13.04509	0.209897	9.129408
1.692363	0.251015	0.15085	0.293549	0.004438	0.177242	1.15352	0.019291	2.506805
27.75485		0.501061	0.689292	0.197727	0.925946	18.00772	0.045066	6.135763
1.473692	0.35686	0.763613	0.657891	0.823481	0.939521	0.980837	0.483249	1.103314
0.742628	2.641937	0.590959	1.26816	0.209662	1.52954	12.15742	0.368184	11.79939
2.021936	0.345358	0.70248	0.698905	0.84612	1.006105	0.814066	0.47342	0.820874
1.99935	1.440256	1.728904	0.710854	0.433919	1.56411	2.583207	1.333733	2.771811
1	1	1	1	1	1	1	1	1
0.743612	0.273703	0.585212	0.405551	0.280215	0.521544	1.355343	0.79091	0.737554
0.965542	1.419117	0.368562	0.943939	0.132544	0.918089	5.13636	0.464501	5.873538
3.668976	1.62366	1.016004	0.639548	0.67268	0.606868	1.068714	0.717379	2.272039
1.373564	1.239715	0.365896	0.459823	0.071816	0.730093	1.254842	0.540529	2.817334
1.446498	1.407074	1.864656	0.283917	0.521307	1.078218	0.74881	0.783938	0.489971
1.020426	0.732109	0.74284	0.826052	1.565189	0.731088	0.820866	0.973756	1.444527
1.494786	1.539379	0.580305	1.22718	0.281334	0.577986	0.564426	0.5561	4.262947
1.333841	2.793273	0.791069	1.4948	0.216151	0.82242	4.261499	1.028907	4.324138
1.023704	5.193143	1.047845	0.644021	0.066869	2.454119	12.87426	0.616505	5.158453
18.227	6.276613	1.317088	0.903343	0.173572	1.425006	8.254071	0.174486	3.48692
2.138984	4.502073	1.309968	2.802769	0.092359	1.482929	11.85937	0.545665	2.106023
1.849663	1.935095	0.668086	1.425753	0.317717	1.252098	3.659673	0.853306	3.259641
0.630713	3.62897	0.476838	1.815918	0.096868	0.869056	16.55658	0.509779	3.960636
0.898174	0.52835	1.5214	1.722858	1.490245	1.191348	1.189516	1.625266	0.667588
0.963784	0.29077	0.62443	0.439815	0.291037	0.298501	0.322227	0.718518	0.485793
10.81052	6.968803	2.031885	1.862846	0.388899	2.19637	44.53774	0.428191	5.376158
4.026236	1.189438	1.200352	1.159686	0.126971	0.522373	0.907386	0.286156	2.167945
4.171494	3.934029	1.80848	2.303274	0.150776	1.11594	1.386724	1.240083	6.349866
0.979983	1.365916	1.346184	1.210578	0.638901	1.367824	1.218226	1.026952	0.692268

Kidney_PT(	Kidney_VW	Kidney_PR(	Kidney_CD	Kidney_TFF	Kidney_TFF	Kidney_TFF	Kidney_SEL	Kidney_C5A
1.618082	3.578411	0.914892	5.692106	10.98911	9.162648	3.879646	2.410364	4.475729
0.689771	3.031839	1.024933	2.45636	5.830498	4.678356	3.294358	5.119811	4.79008
20.15517	12.87478	18.50614	62.74389	13.68899	16.74009	7.582413	24.90384	28.8247
1.932857	3.704499	0.878263	7.747412	6.90427	9.699717	2.744013	4.90011	9.966502
3.908874	4.562978	2.171122	10.86409	2.690986	3.198444	2.922025	3.795133	29.52239
16.90554	1.525868	87.92412	1.879674	13.0141	7.31785	5.386229	86.50613	46.47293
1	1	1	1	1	1	1	1	1
1.343108	0.25401	0.999894	0.971571	0.909511	0.166786	0.260678	14.5582	3.633242
1.149133	3.202734	1.960042	3.025912	5.70843	19.04964	4.776212	1.467577	8.60211
69.65625	2.774638	49.00389	21.74304	7.498534	1.033336	9.363383	297.1112	15.49427
0.892476	1.152249	0.415304	0.766974	0.542381	0.641368	0.570602	2.291713	1.341238
2.201334	2.860043	1.256756	2.304419	1.41225	0.789347	1.011219	3.774213	1.669259
14.94644	0.549022	2.375008	1.701399	0.332788	0.069952	0.443939	209.2635	3.04939
0.305012	0.058495	0.78011	1.351231	0.405097			132.4545	2.358206
9.71698	0.17073	1.710726	0.200592	0.16825	0.01563	0.235575	203.5777	2.328539
0.645483	0.972269	0.747019	0.172887	0.532885	0.58522	0.501866	2.407997	5.005608
13.47148	0.362357	0.943442	2.402734	0.688791	0.116292	0.816895	76.65233	2.179344
0.542088	0.54277	0.652085	0.219051	0.624258	0.333437	0.437656	0.991598	1.574796
2.58413	3.48513	4.484655	4.067675	1.085865	0.567882	0.869214	62.71843	3.593193
1	1	1	1	1	1	1	1	1
0.972512	0.780784	0.693175	0.562732	0.656045	0.370692	0.522011	1.88584	4.421217
4.226379	0.31727	1.278539	3.264792	0.237774	0.120042	0.429084	49.60468	2.166732
0.159596	3.220097	0.024355	0.843389	0.225396	0.15232	0.896937	6.011864	0.974537
3.644582	1.545261	0.082605	0.178603	0.284421	0.061313	0.939236	2.537934	0.150516
0.598155	6.385696	0.113213	0.294861	0.613223	1.575364	3.291672	0.557653	0.207232
0.871969	5.262016	0.274602	4.674749	0.562231	1	0.184859	1.12626	1.532326
0.6305	1.001584	0.035107	0.282064	0.715993	0.300644	2.526779	2.475167	1.328957
5.765329	3.788974	0.192434	1.253932	1.25172	0.71412	2.424352	22.02519	2.267829
14.27075	2.530843	0.079257	0.341681	0.923592	0.056179	2.09529	3.010327	0.581103
44.98771	3.771622	0.356396	6.933409	1.367373	0.382925	1.915303	12.801	0.870125
3.846302	0.978158	0.166436	2.35881	0.795261	0.105139	0.476462	7.014522	0.607962
1.447856	2.510743	0.189983	0.770692	0.235737	0.289793	2.145779	1.816069	1.171026
0.959044	3.016033	0.138868	0.929928	0.167691	0.052145	1.030552	8.584739	0.252176
0.528047	13.74778	0.097398	0.875716	1.780753	0.507452	1.965262	0.589438	0.366859
0.55933	4.677747	0.034909	0.083931	0.947957	0.31362	1.127393	0.451839	0.76774
36.10502	2.298122	1.498555	4.601005	0.375025	0.101269	2.756	68.53741	0.451712
0.762018	4.316045	0.124136	0.138187	0.607114	0.185265	0.969611	14.72554	2.569537
2.985954	1.807629	0.225189	0.365955	0.977111	0.473702	1.216085	10.89623	0.938146
1.146829	0.190041	0.060422	0.213915	1.778628		5.409529	0.887895	0.652602

Kidney_VE	Kidney_SE	Kidney_ST	Kidney_OA	Kidney_OA	Kidney_OA	Kidney_OA	Kidney_FO	Kidney_MX
7.550772	0.785563	0.970788	1.355517	1.65738	1.129459	3.83364	1.229824	5.938431
5.737226	0.7429	1.409685	1.282556	2.457377	1.456103	2.41842	0.830025	5.132477
113.3095	11.37003	9.368323	3.290067	20.64553	10.20125	7.570303	12.45538	27.79988
7.499575	1.553318	1.672566	2.982856	4.063455	2.254855	12.34681	2.629598	8.345778
14.2351	5.75104	2.162551	3.062379	5.804372	4.384893	50.93285	1.726207	15.0424
18.74968	61.29244	22.4392	6.101658	24.7359	31.28116	65.00273	2.271371	377.3544
1	1	1	1	1	1	1	1	1
1.919823	4.030449	2.107893	2.982706	2.702402	5.758777	16.06108	0.179873	13.31603
20.42152	1.197071	3.89831	1.514393	2.393733	2.060194	1.923194	0.638893	9.813186
3.847415	72.90743	47.0799	83.49464	27.70148	20.69409	83.87313	5.005311	614.4603
1.737274	0.863579	0.014596	1.113297	0.433684	0.684212	0.299975	13.32225	1.112526
2.630672	0.793016	1.414524	1.630194	0.656333	3.500858	0.84348	18.08933	1.54928
0.655842	8.10844	4.487407	53.2961	3.094854	8.616383	4.635211	253.4276	509.9571
1.134609	0.095207	3.051156	3.358378	2.57671	3.545762	3.858326	8.401141	6.757729
0.274931	18.13624	4.938547	45.42375	0.652782	6.094467	9.99277	51.36713	57.3555
1.80827	0.767276	0.879088	3.345849	0.73639	0.472996	1.174374	19.54166	2.903521
0.579752	16.5402	9.351271	45.01132	1.390126	3.865663	4.387363	27.58515	20.70556
1.113328	0.414558	1.015427	12.42047	2.953844	1.1787	4.335767	9.36337	2.844199
1.298859	4.580058	0.838315	1.637266	0.733265	0.786157	0.809477	44.02319	0.923478
1	1	1	1	1	1	1	1	1
1.521513	1.098912	0.355783	1.348389	0.533049	0.977021	2.827769	25.66615	0.565964
0.477581	33.94944	1.898226	8.810006	4.079782	8.014408	2.625408	12.16294	21.48021
0.223136	0.445942	2.769634	5.428658	3.69269	5.39426	5.860832	4.214173	5.050679
0.154062	0.832791	2.135341	6.074734	2.178765	4.183174	3.263528	2.295268	8.018011
0.362859	0.094668	0.572418	0.676701	0.770857	0.709319	0.860643	2.69994	0.881915
0.483791	0.96708	0.828633	1.105317	0.96387	0.948466	1.034855	0.628787	0.639584
0.546899	1.036801	2.207552	6.590964	2.855589	3.252454	3.586691	0.808226	3.696715
0.784273	0.811855	1.84974	5.002817	3.389983	4.176974	4.432923	2.660776	3.924636
0.675913	2.131662	7.410609	55.54061	5.851673	13.18632	10.35894	4.641929	62.77758
0.43121	2.37201	6.889722	176.232	3.451295	3.915458	7.419172	32.70618	190.4934
0.413112	2.339845	1.973598	10.77244	3.022698		3.396686	2.587232	8.747368
0.30964	2.363511	2.379186	4.432782	2.374057	3.576378	4.321529	1.168514	3.481418
0.274532	6.582252	1.900199	7.367572	2.039263	3.675006	1.682235	3.301288	34.23755
0.637229	0.589871	1.310141	1.187248	1.468511	0.89569	0.942749	0.915309	1.893131
0.864737	0.128453	0.429966	0.400504	0.460961	0.38722	0.38133	0.425156	0.940895
0.367233	2.827165	3.849412	16.8803	4.598807	6.506636	10.53265	9.926493	43.40602
0.218315	3.446665	2.971856	8.383599	4.800325	4.216299	3.296894	2.871544	8.281086
0.820601	1.959315	2.488977	7.070918	3.804859	3.454468	5.139757	3.087716	8.136296
2.06701	1.034041	1.206807	0.904718	1.037484	1.054334	0.966319	1.590363	1.563517

Kidney_IL1	Kidney_IRF	Kidney_IRF	Kidney_IRF	Kidney_CX	Kidney_CX	Kidney_IFI	Kidney_CL	Kidney_HA
1.135122	3.602059	1.644555	2.135557	1.832207	4.503615	3.289684	3.380111	0.243592
0.436549	1.686533	1.730471	1.225431	1.262316	1.05348	1.316296	1.424945	0.309917
4.040917	9.686005	8.026846	11.28967	14.10998	5.333953	10.27407	10.4203	22.51867
0.607427	4.650833	3.93549	1.391198	9.560688	2.615224	4.426814	6.471454	0.572959
1.071471	1.450692	3.100944	1.511708	8.099558	8.223731	1.567028	1.052703	2.442122
5.520664	15.43884	129.2883	22.81724	328.0641	727.1164	14.16893	78.09421	587.405
1	1	1	1	1	1	1	1	1
0.358944	1.889755	6.248297	0.996596	12.9667	88.66794	1.335729	7.047968	15.08155
1.448632	3.411605	2.722124	2.3078	3.189412	3.923905	2.230911	7.926777	0.183987
2.81681	36.9188	68.33384	25.01193	269.2039	1230.576	22.5029	89.65955	37.84671
1.755665	1.08721	0.532624	0.465317	10.62138	2.948518	1.210731	2.093598	2.603401
2.820414	1.363016	1.394126	0.870426	2.210994	2.670536	3.457726	3.87261	0.896762
2.151224	4.285979	9.087297	5.210411	130.9162	1014.821	13.3362	5.171677	11.08423
1.568388	1.445519	16.18403	0.320952	6.697089	142.7109	6.442898	10.4112	43.98008
4.265352	6.059991	16.09158	27.63754	1456.449	258.6611	9.471777	8.111839	175.2228
1.414208	0.433079	0.487169	1.49369	1.40714	1.423081	3.534006	2.134811	4.470602
5.015081	3.880005	28.37303	1.381057	35.01613	262.7096	22.1466	8.075992	39.53079
4.935949	0.526246	24.59161	0.560037	1.295844	51.99972	4.138797	1.385961	9.77013
2.333754	1.118267	0.801261	0.821021	3.992155	3.833995	2.062194	6.032653	11.51217
1	1	1	1	1	1	1	1	1
2.51034	0.690341	1.536053	0.950353	0.535558	0.783014	5.956618	3.05474	7.658426
0.969144	2.215597	8.046736	2.53057	92.04742	53.82385	8.8686	14.78371	136.6838
0.746381	1.430896	11.32888	0.702857	3.048654	30.34377	0.743885	0.315852	40.86505
0.55541	1.785752	16.40534	1.826591	6.341074	12.54088	1.376417	1.011957	55.46184
0.730385	1.382726	0.761613	1.18462	0.136248	0.83431	0.421012	0.193561	2.959964
0.525959	1.264125	1.537592	0.619315	1.488776	1.153328	0.401946	0.231809	0.898862
0.751678	1.489504	23.87347	1.654972	1.17298	12.43774	1.210856	1.703059	37.36908
0.54122	1.644478	10.8668	0.657098	7.594115	43.02949	0.569307	2.711824	21.59044
0.748664	4.088753	10.30953	1.707854	56.25378	321.6508	2.635084	1.512995	3.593746
0.542223	10.67984	5.066211	6.902737	45.90221	167.0014	5.011439	3.308253	1.224871
0.730642	0.838608	4.505553	2.33101	11.50431	50.95279	1.49148	3.419559	81.63431
0.716748	1.267642	9.2796	1.230836	3.908571	40.34837	0.742297	2.250267	72.69756
0.74422	4.587812	13.89017	2.0204	7.657814	71.69143	1.677958	7.501712	41.54528
1.229446	3.343009	1.79173	2.280087	0.26602	0.950608	1.640406	0.657623	0.505857
1.244154	1.765281	1.041269	1.158905	0.1658	3.109936	1.054382	0.673194	6.889959
1.898824	4.937506	7.627653	6.685005	93.39046	311.1584	3.296465	7.250023	195.5063
1.040131	1.864114	14.34431	0.987879	2.345847	31.3259	1.264659	1.948561	111.8757
1.01998	2.265803	12.45596	1.554711	7.80922	55.52307	2.350681	3.243923	184.2342
1.901291	3.428522	0.650367	1.614686	0.671693	0.867056	2.487898	4.313905	1.112518

Kidney_LCI	Kidney_DD	Kidney_KLF	Lung_ICAM	Lung_SELE	Lung_IL6	Lung_SOCS	Lung_STAT	Lung_CEMI
5.200164	5.13886	1.40389	0.621084	0.351414	1.152132	0.565454	0.700751	0.486167
10.73199	2.743846	0.773832	0.651046	0.367264	1.011383	1.042525	1.722024	0.735775
28.34154	15.258	12.35783	0.594348	0.516205	0.89365	1.670416	0.885036	1.093703
5.300043	4.886144	1.99551	1.394163	0.285482	0.984176	0.704382	1.178391	0.667091
10.98367	3.334315	1.89081	0.868789	0.258318	0.392398	1.0206	1.249934	0.511702
33197.38	17.98816	2.912641	2.367168	13.11634	164.8695	10.27047	3.827644	21.51511
1	1	1	1	1	1	1	1	1
11547.12	2.664411	0.447268	11.39956	36.31614	101.9544	6.781499	1.291817	19.03781
9.348403	4.260629	1.335439	1.019757	0.508402	0.808003	0.702066	0.556043	0.771481
59505.17	24.32259	3.090663	2.716508	9.982392	326.9565	16.12985	2.446129	16.06376
			6.629145	20.52717	71.38962	6.175641	1.363394	14.11264
0.345695	0.592299	0.374333	0.958377	2.14425	0.432103	1.43424	0.571679	0.49359
0.749282	0.855562	0.857116	1.154005	1.058821	0.706892	1.397534	1.083612	0.999717
2739.989	14.44044	0.815355	7.298854	18.59433	259.349	36.65689	4.251097	15.23139
292.9192	1.925682	0.578188	30.41739	1.727627	7.055725			
1720.834	4.228446	0.820591	2.017122	39.2774	926.9563	19.80015	2.906463	15.37839
0.242277	1.255387	0.60743	0.948037	2.079197	0.942379	1.39885	1.014777	0.588622
6446.186	6.24284	1.396912	5.362327	31.50528	214.6922	12.05236	1.992884	33.26403
7.403231	2.485056	0.394313	1.865343	1.174215	1.168124	1.478285	1.765224	1.072512
118.4285	0.813299	1.060428	1.047213	3.776918	1.270384	3.267707	2.022249	2.336057
1	1	1	1	1	1	1	1	1
1.300479	0.843965	1.277393	2.353416	7.210053	1.90512	3.472316	1.101267	1.583726
2442.109	1.918481	1.162541	2.353416	7.210053	103.2129	3.472316	2.433456	14.84042
227.8639	0.853746	0.550768	4.059203	51.37915	145.9443	6.119209	2.850642	12.69425
267.6129	2.429125	0.468264	2.247911	36.91323	96.67953	9.053534	2.333404	14.61675
0.125777	1.55645	0.698151	1.169524	1.686078	2.219355	3.263237	1.243452	2.583754
0.52939	1.404186	0.703559	0.973357	1.505768	0.936536	1.409803	1.563828	0.507164
370.5148	0.665032	0.453514	5.613391	100.4459	54.23271	3.585972	2.40997	21.20079
612.128	0.427278	0.386843	3.15277	20.07462	84.74296	3.98934	1.741543	9.029514
459.9302	3.066318	1.371643	2.278363	20.13014	335.0484	11.3093	2.340049	24.15548
404.695	5.192288	1.304132	3.501664	59.46798	5087.134	32.66111	5.388775	52.90717
444.5444	0.977295	0.62469	2.461637	36.15207	83.57891	4.834408	1.173409	7.262508
665.3206	1.149242	0.258841	5.492868	51.89752	76.59388	7.276247	2.739948	9.899358
2050.22	2.166876	0.416342	5.665259	36.57416	216.3096	22.5418	4.089543	51.44162
0.767225	3.359108	1.376708	0.64533	1.231414	0.59554	1.618408	1.464158	0.779468
0.821296	0.627104	0.456728	0.837679	0.974851	0.873268	1.228944	1.264836	0.89023
1601.139	1.445369	0.249562	1.374992	10.80178	912.7319	16.63748	5.011048	31.02326
580.7737	1.782308	0.265786	3.512876	23.65379	23.71977	5.756026	2.344706	18.24352
746.8935	1.032717	0.601628	3.311173	40.91779	130.6038	6.115723	1.565559	26.28923
1.888965	1.503687	1.421345	0.438912	0.664113	1.067764	0.709319	0.639457	1.971748

Lung_F3	Lung_RHOI	Lung_TSP1	Lung_PLAT	Lung_PLAU	Lung_THBD	Lung_PLAU	Lung_PTGS	Lung_VWF
1.008212	0.429947	0.548266	0.761477	0.97766	0.691595	0.624948	1.047059	0.807341
1.00533	2.077038	0.830573	1.082035	0.942485	0.902756	0.904664	1.61296	2.048201
1.942887	0.691314	1.136824	1.362688	0.611988	0.879946	1.098489	1.827451	0.836768
0.982752	1.25269	1.011491	0.844521	0.639872	0.578091	0.563315	2.623861	2.134258
0.623162	0.87686	0.573215	1.330934	0.469761	1.468612	1.244162	1.11282	1.625369
1.959117	3.54081	0.848389	6.961253	0.129693	0.14522	6.289172	2.905875	0.147471
1	1	1	1	1	1	1	1	1
0.979284	1.3992	0.61784	6.41223	0.593206	0.161156	2.622702	8.918183	0.296829
0.877194	0.809876	0.877147	0.60083	0.344317	0.570867	0.452686	1.900672	1.249303
1.178513	3.735182	0.684173	6.566507	0.228906	0.132421	3.359537	2.746183	0.163805
1.108493	1.840734	0.643267	9.020696	0.100843	0.207836	1.978521	3.568797	0.235574
0.569387	0.602406	0.922521	0.514921	0.472801	0.729099	0.611973	1.551305	0.960865
0.634117	0.940477	1.851745	0.842927	0.812816	1.354592	1.047163	1.061734	0.837064
1.038079	6.049721	2.918542	4.769618	0.449043	0.144033	7.573677	2.762837	0.343502
0.299648	0.468684	1.535858	2.080363	0.373625	0.241998	1.221207	1.202141	0.323466
1.699282	3.490968	5.122838	3.447943	0.310968	0.177401	9.580325	1.534121	0.162718
0.690255	0.806792	1.209367	1.01179	1.020558	1.103705	0.735846	0.985028	0.842836
0.773889	1.701685	0.993323	6.436819	0.631449	0.191044	4.639785	7.229181	0.546705
0.724722	1.26436	1.130966	1.073734	2.601537	1.839934	1.426636	1.978545	1.888172
0.655082	1.712002	2.706557	1.672171	0.24149	0.733284	1.486933	1.23688	2.499035
1	1	1	1	1	1	1	1	1
0.853566	1.164457	1.693874	1.064179	1.29687	1.090616	0.937516	2.438954	1.141362
0.74122	1.860151	0.82018	8.720695	0.728309	0.238184	3.457411	3.428117	0.460046
0.858794	2.784236	0.841584	5.974723	0.863941	0.474451	2.161019	5.058818	0.342742
1.356004	3.495221	0.97124	7.417441	0.376587	0.258369	3.997975	3.607997	0.34789
1.234751	1.755106	2.505522	0.613768	1.0589	0.964774	1.850414	1.787655	1.517994
2.040473	1.820025	1.107402	0.769225	1.272602	2.088113	0.970852	2.054294	2.279332
1.879406	3.366513	1.754259	15.94382	0.577034	0.460654	4.610916	3.138655	0.487414
1.008477	2.226862	0.621196	7.231145	0.441708	0.238321	2.279593	2.586348	0.220932
1.212091	2.659614	0.919301	14.76665	0.321156	0.273647	5.582337	3.948264	0.398476
3.920089	14.45242	7.478782	12.87351	0.574642	0.367996	16.63485	9.929505	0.323448
0.969442	1.154742	0.594398	11.17564	0.305123	0.22377	4.272135	3.072529	0.176596
1.889521	1.769551	0.948453	9.920248	1.157422	0.445757	4.129828	4.008709	0.459137
4.152373	3.761207	1.337263	24.25441	0.883023	0.391402	8.809086	3.770807	0.242346
1.965699	1.618109	1.257743	4.436924	1.338017	1.997367	1.726844	0.874597	1.657853
1.460904	0.84363	0.727117	3.270207	0.581408	1.23836	1.276666	0.854441	1.531053
4.864892	3.848367	2.066156	10.66873	0.469997	0.146534	10.81498	2.871971	0.100756
1.264402	1.527847	1.754885	8.08821	0.388881	0.504043	2.811267	2.395303	0.442612
0.826384	1.558415	1.653277	7.887486	0.278642	0.399146	3.369634	1.633756	0.220406
0.490082	0.549443	0.903015	1.300009	0.785792	0.478901	1.030023	0.486785	0.438725

Lung_PROC	Lung_CD44	Lung_TFPIA	Lung_TFPIB	Lung_TFPIC	Lung_SELP	Lung_C5AR	Lung_VEGF	Lung_SERP
1.583058	1.139176	1.488493	1.403676	1.529417	0.31811	0.383304	0.20945	0.435087
1.637281	0.904038	1.717188	1.278593	1.577407	1.578212	0.482245	1.152854	0.500959
1.265927	1.919529	1.081591	1.149887	1.003072	1.4969	4.88721	0.396189	0.679428
1.361604	0.731589	1.779	1.512112	1.415588	3.958487	0.928404	2.159048	0.626927
1.02966	0.621874	1.614524	1.551883	1.038414	0.925527	0.8601	0.820833	0.908807
13.47138	1.494245	1.089698	0.284942	1.350779	98.9173	7.596611	0.46498	20.83983
1	1	1	1	1	1	1	1	1
7.495997	0.816356	0.743481	0.213531	0.77069	43.78746	7.474888	0.150072	19.71226
0.924786	1.823337	0.944689	0.864337	0.7917	0.953951	0.993344	0.315472	0.599222
5.834948	0.992532	0.76359	0.103945	0.955972	50.02585	6.772746	0.179954	6.457361
5.643699	0.892716	0.858795	0.324501	0.756201	17.51195	9.497119	0.198239	11.0948
	0.932581	1.230682	1.170234	0.992928	1.955491	1.010247	0.667893	1.027792
	1.2117	1.373404	1.158135	0.7968	1.824415	0.64335	1.031661	1.674551
	1.741777	0.578324	0.197301	0.676662	55.39461	12.49747	0.645716	11.2436
	0.641924	0.965449	0.610126	0.649485	24.70005	1.716076	0.200011	20.99388
	1.992465	0.579278	0.132602	0.549259	106.2707	8.342281	0.264151	21.19955
	0.761556	1.058128	0.973591	0.977885	2.95124	0.57285	0.740254	1.245793
	1.082374	0.646769	0.177154	0.612451	23.48229	7.221186	0.689398	23.77842
	0.621869	0.816403	0.952249	0.822702	0.913389	2.091222	1.150515	1.04153
	0.730355	1.343445	0.801787	1.147498	5.306311	3.270944	0.772779	2.235449
	1	1	1	1	1	1	1	1
	1.757065	0.780746	0.804772	0.736591	0.960045	1.194037	1.052191	1.091321
	1.543663	0.89433	0.287474	0.886158	27.47807	13.0911	0.742827	26.42723
8.483696	1.180014	1.1519	0.405851	0.95352	34.30583	1.394397	0.167299	25.73082
9.105125	2.019425	0.615625	0.324689	0.720989	43.91123	3.741908	0.106999	23.47194
2.563212	3.334646	1.304417	0.785299	1.523763	0.798449	0.147588	0.661616	2.017196
1.5947	3.334002	2.40473	1.392206	2.128683	1.603543	1.58374	1.418535	1.667049
13.41694	3.043924	0.70477	0.564896	1.052556	24.63421	10.30214	0.459601	61.01154
9.958829	1.616175	1.514261	0.450622	1.071138	45.77661	0.871604	0.337727	25.49211
36.46589	3.806922	0.869945	0.117478	0.582321	81.02728	5.459564	0.257797	76.54726
18.46905	0.38287	0.19484	0.180944	1.211379	102.9318	1.574685	0.172506	31.79112
31.32646	0.607778	1.069438	0.129185	0.166011	156.2101	6.911668	0.305012	45.75123
10.62479	2.125462	0.748717	0.430124	1.456805	44.06564	2.618978	0.495431	30.35386
28.2467	3.359946	1.188439	0.160832	0.894179	151.8738	2.67823	0.756111	23.71455
4.692415	3.319743	2.13789	1.338492	0.903074	1.336593	1.945005	1.818039	5.062488
1.556427	1.392251	1.933491	1.381818	1.076996	1.385561	1.26145	0.465198	1.116888
15.99673	2.732967	0.820032	0.233229	1.29474	68.86903	0.394697	0.383298	29.8431
5.700794	1.035634	1.565117	0.650633	0.690651	19.75798	0.971496	0.07677	23.73079
4.759914	0.704002	0.414215	0.535238	0.633364	6.011435	3.752798	0.1961	25.77914
0.627077	0.29994	0.415847	0.718284	0.469774	0.623619	0.631417	0.704952	0.599863

Lung_STAT	Lung_OASL	Lung_OAS2	Lung_OASL	Lung_OAS3	Lung_FOS	Lung_MX1	Lung_IL18	Lung_IRF1
0.813316	0.64326	0.693336	0.948527	0.424692	0.630025	1.009758	1.250724	1.065526
1.074643	1.532526	2.256412	1.713796	1.736932	0.943891	2.221763	1.246571	1.045307
0.807384	1.053378	1.414791	1.507059	1.643338	0.835492	0.664995	1.534024	0.99487
1.352835	1.017932	1.094384	1.329647	0.835512	0.960296	1.271511	1.033523	1.44707
1.161819	2.181376	2.582326	1.740152	1.589219	0.700275	0.828816	1.156067	1.213614
3.172866	9.067052	2.839938	4.210862	5.823419	1.38096	9.182159	0.639519	1.832912
1	1	1	1	1	1	1	1	1
2.544314	9.177183	3.909525	4.201304	10.85614	0.624529	5.241675	0.571189	1.782514
0.762441	0.59618	0.709209	0.795743	0.459252	0.789178	0.68815	1.13331	0.860677
4.408499	22.71788	5.681791	5.547926	8.546993	1.835735	31.35689	0.433555	3.777702
2.560779	11.07217	4.006155	5.391622	10.69505	0.329479	5.718718	0.815578	1.629474
1.041738	1.295844	1.785994	1.054683	1.356709	3.365325	0.743469	0.902196	1.064441
1.267642	1.138456	1.638912	1.390589	1.2292	2.208429	0.753604	0.712555	1.054762
5.719489	93.39447	9.094917	7.075874	15.68993	10.86526	79.95781	0.551978	6.602616
2.82326	9.088943	5.379084	5.50712	9.450951	3.809797	5.916387	0.79396	1.496876
4.333521	131.2546	7.69941	5.129906	20.25966	13.14314	64.01015	0.44895	3.540843
1.027147	1.13986	1.414853	1.261367	0.644719	2.917339	1.043987	0.950819	1.020959
3.78778	18.9789	4.156272	7.635437	16.78108	7.334597	14.49344	0.709412	2.594818
1.476049	2.306443	2.096942	1.94222	2.35402	2.267314	2.726413	1.58225	0.987344
0.926214	0.923538	1.812454	1.46787	3.095451	6.724788	0.572032	0.550571	1.07672
1	1	1	1	1	1	1	1	1
0.873955	1.522014	1.167155	1.165177	0.863157	3.479343	0.948364	1.373996	0.80459
4.481532	19.77959	7.107743	7.569893	29.7575	5.799186	8.398786	0.599967	2.081455
2.921612	12.84233	5.823241	7.909149	9.480604	6.301278	5.705438	0.990302	1.540872
3.649717	12.40583	5.315951	6.794009	5.344318	4.548935	6.28394	0.579153	1.34016
2.702638	2.198475	2.613205	2.898486	1.644326	5.46794	3.573095	1.68096	0.641092
1.772227	1.987448	1.943083	1.444453	1.596188	1.991884	1.800188	1.59336	0.848926
5.890732	14.47093	7.468299	10.83565	3.588377	3.624709	9.412426	1.072252	1.086151
3.368383	7.769434	4.56704	6.148382	3.992221	2.391783	5.548194	0.611753	1.011892
43.58324	160.3653	41.54739	52.76907	9.023493	28.1565	94.24741	1.677455	2.971029
16.60374	233.8148	17.62383	13.41633	13.26843	27.27057	172.8386	0.908543	7.830647
5.18147	11.8372	9.632193	8.945507	4.045116	7.454573	9.171198	1.071553	0.830699
4.771574	5.21778	5.056478	7.621237	4.105426	2.056078	3.32813	1.128657	1.029404
10.348	18.98841	5.998106	10.53161	6.68828	5.371114	27.06444	1.114389	2.587696
8.346507	4.803925	7.607415	6.389306	1.631023	4.751709	1.683632		1.177606
2.347981	2.627419	2.430218	2.583006	0.534787	1.183345	1.552645	1.273918	0.697299
2.768274	13.41105	3.042274	4.505044	9.655059	6.562041	12.24573	0.368553	2.77076
3.707968	10.66287	5.742356	7.340282	8.679844	1.992261	5.793887	0.903641	1.204185
2.181145	7.340388	4.62071	4.563509	4.782598	1.603879	3.468245	0.42747	1.239861
0.564262	0.503158	0.514646	0.692304	0.626493	0.502037	0.555498	0.627604	1.17796

Lung_IRF7	Lung_IRF8	Lung_CXCL	Lung_CXCL	Lung_IFIH1	Lung_DDX5	Lung_KLF2	Liver_ICAN	Liver_SELE
0.912463	0.911668	0.317822	3.505235	1.204914	0.899921	0.750718	1.097665	1.142831
1.427107	1.215659	0.65303	2.044911	1.383065	1.158654	0.611308	0.668434	0.967041
0.872648	0.621996	1.03921	1.081554	1.053674	0.984547	1.094319	1.015899	1.36882
1.252294	1.174271	0.282102	1.22795	1.434801	1.375432	0.985508	1.363407	0.439799
1.524784	0.791593	1.090819	0.906489	1.351816	1.323575	0.844685	0.852297	0.753091
12.01161	1.418695	30.1209	593.5298	2.588578	1.622529	0.760578	7.224862	1.91586
1	1	1	1	1	1	1	1	1
9.393189	0.808105	38.65234	301.0334	2.228809	1.938172	0.629909	1.547331	0.960612
0.677468	0.77829	0.380347	0.478701	0.99137	0.789661	0.627549	4.721572	0.933415
17.31768	1.939226	40.66857	1692.092	3.659878	2.326459	0.783172	7.963836	1.378339
10.75717	0.71524	25.25159	415.7267	2.522673	1.811711	0.507417	3.649977	1.335602
0.745183	1.21742	0.424843	0.661389	1.417546	1.81726	1.056556	1.354365	0.484804
0.865455	1.086826	0.812863	0.810433	1.47726	1.785408	0.735132	1.962121	0.573426
15.89637	3.477641	518.3292	1847.898	5.053892	4.314661	2.532123	15.22397	6.412264
15.65428		166.8273	156.0345	2.637564	2.487741	0.409979	7.88156	2.319015
20.99388	3.158411	2437.928	1852.861	4.257813	3.328537	1.690183	15.10438	5.131249
1.471406	1.091656	1.390174	0.596445	1.169229	1.293403	0.535123	1.013136	1.093706
16.52967	1.844535	258.2695	1688.293	3.052177	2.328364	3.826819	10.61421	1.17318
2.738016	1.165759	5.636355	1.359853	1.431005	1.217544	2.834124	2.251394	0.290246
0.87147	0.703151	10.2078	0.48206	1.140128	1.081759	1.673456	0.327189	0.627676
1	1	1	1	1	1	1	1	1
1.054567	0.877805	2.420026	0.700314	0.921972	0.888898	1.607908	1.009709	0.171895
11.93393	1.44693	144.3432	838.3742	3.811409	3.884219	4.971886	4.848416	0.981759
19.12926	1.164591	4.8367	53.17853	2.408311	2.162445	5.336936	3.757301	1.70472
15.6283	1.219753	8.425333	36.16973	1.974848	1.637168	0.683755	2.117994	3.093067
1.092875	0.663626	0.658403	1.023685	0.826588	0.738696	1.153112	0.956435	1.958879
1.067235	0.813844	1.787118	1.35138	0.75305	0.807878	0.921554	1.383957	0.327738
11.65716	0.861678	2.543722	12.64766	1.734281	1.403825	2.390058	5.975232	2.03219
11.93081	0.95041	5.577867	32.3697	1.661967	1.110118	0.761932	5.771125	4.41065
10.45852	2.954121	29.01146	89.4303	4.873122	2.938054	0.053263	22.49837	15.54818
6.939723	6.942476	77.18795	257.2242	8.213913	6.842198	7.791137	29.32253	50.06773
7.349691	1.193599	16.08654	27.97721	1.749422	1.271273	4.375321	4.469591	2.84844
12.46221	1.05063	2.342678	12.70522	1.508001	1.270323	0.631734	1.373827	2.458365
14.70265	2.125993	20.32246	94.9036	2.799996	2.455555	8.897173	10.17732	3.595288
1.59191	0.986201	0.370543	1.204003	1.181574	1.170189	6.945358	1.236005	2.50345
0.967372	0.689389	0.657346	1.370978	0.809893	0.627895	3.42635	1.236628	0.634857
13.88376	4.624707	44.12656	117.5193	2.337866	1.798032	6.138773	21.39476	7.586534
15.9829	1.049052	3.99621	14.26988	1.507413	1.632334	4.710817	5.65675	2.21195
12.12261	0.893291	3.891708	15.36225	1.633958	1.581729	2.415854	1.616502	3.466331
0.937001	1.228737	0.55956	0.739984	1.327933	1.237811	1.085123	0.722566	3.051216

Liver_IL6	Liver_SOCS	Liver_STAT	Liver_CEMI	Liver_F3	Liver_RHOI	Liver_TSP1	Liver_PLAT	Liver_PLAU
0.345844	0.973153	0.498498	0.194902	4.942041	2.704289	1.032015	1.516717	1.551397
1.025394	1.698094	1.259763	1.750906	1.693952	1.168934	2.00828	1.229924	1.517022
0.681423	1.602833	2.706507	0.231667	1.348059	3.218861	0.817259	0.345934	0.199947
1.629294	0.922549	1.174951	0.191519	3.293104	2.366473	1.580296	0.267519	0.421906
0.689398	0.29362	0.82454	1.580375	0.767496	1.97057	1.700821	0.750612	1.103284
13.68776	30.43622	2.087857	52.18084	0.418558	0.878534	1.806806	2.614432	0.524168
1	1	1	1	1	1	1	1	1
1.030334	0.963092	1.318332	1.918909	0.68318	1.47596	0.73878	1.954949	1.290005
5.214167	12.14035	1.342916	28.54234	0.376163	0.641533	0.878804	1.318653	0.741147
14.3275	28.93313	2.071137	64.56097	0.564757	0.695973	1.204706	2.331999	0.388934
2.381299	9.644336	0.735658	4.586912	0.164369	0.379888	0.356745	0.318337	0.117652
2.466546	8.825814	1.906188	2.116284	0.531025	1.470634	0.264304	2.145209	0.36268
2.817756	9.323619	2.148561	4.818077	0.690958	2.869299	1.274111	1.591008	0.820385
73.4939	86.18866	2.803788	57.76101	0.649423	0.766666	2.701127	0.709944	0.583698
5.607961	30.83598	1.682867	2.234066	0.484862	1.333947	0.781284	5.407856	1.25241
128.3318	88.44058	3.663703	68.40126	1.081533	1.324308	8.057616	1.748015	0.406642
0.912531	2.806002	1.055568	1.502207	1.135157	1.143413	0.320799	1.234958	0.478674
104.0114	62.02085	1.702184	47.26019	0.438919	1.024179	1.388473	1.177717	0.523193
1.253116	1.424169	1.136653		1.174549	1.264673	0.442923	1.107198	2.181979
1.211601	19.06834	1.199058	38.54589	0.384725	0.777456	1.024902	0.676765	0.434813
1	1	1	1	1	1	1	1	1
1.141016	3.895996	1.103864	0.895449	0.957496	1.075213	0.269872	1.433321	0.542506
17.50751	31.64454	0.848809	6.489343	0.132671	0.465951	0.366681	0.976477	0.133829
4.776257	6.90979	0.891108	0.250317	0.389862	0.453756	1.83208	1.358644	0.251944
2.619996	9.86349	1.379249	1.97013	0.227177	0.311517	2.30944	1.353017	0.619648
1.311282	1.954286	1.226506	1.841966	0.529038	1.905098	2.813623	0.155995	1.450412
0.60917	0.929806	1.148306	1	0.773073	0.799458	0.779032	0.040074	0.251319
3.088396	2.684712	0.50677	0.114207	0.331154	0.287506	0.905186	0.627949	0.172898
11.42562	9.535965	1.039304	0.664591	0.713538	0.728727	1.746353	13.00698	0.331001
29.32412	14.04696	0.82243	7.64091	0.444156	0.25983	3.163975	0.609976	0.508453
130.4162	81.70223	3.387168	31.65894	1.468842	2.953082	16.42203	9.235265	1.021094
5.138846	7.047562	0.612625	4.172845	0.398231	0.340432	2.360763	1.577563	0.202614
2.43977	4.961154	0.317325	0.338024	0.267198	0.257973	0.339792	5.582204	0.11108
14.24785	13.04434	0.567616	4.191207	0.834802	0.300024	2.612195	12.6759	0.291915
3.424878	2.346431	1.106153	1.186551	1.249781	1.177946	1.702467	12.70451	0.767181
1.665459	2.625035	0.952081	0.275595	2.236766	0.551439	0.794417	1.597244	0.287698
68.23002	52.82236	1.795477	25.23554	0.795557	2.058536	4.764712	12.19213	0.534184
7.958584	15.9924	1.199457	1.713216	0.553925	0.60654	1.927023	7.572845	1.213902
2.906263	12.18332	1.060653	0.216711	0.409842	0.580555	1.66998	9.714219	0.519601
1.641579	1.075493	0.870848		1.293539	1.250848	1.283644	24.9536	3.979008

Liver_THBD	Liver_PLAU	Liver_ET1	Liver_PTGS	Liver_VWF	Liver_PROC	Liver_CD44	Liver_TFPIA	Liver_TFPIB
0.91594	0.498558	1.077652	0.398927	2.675456	0.709068	0.134515	1.528053	1.687396
0.673938	4.197656	3.101252	2.152627	5.06389	1.279465	0.801129	2.376343	2.421789
1.637956	0.801111	2.151201	0.474178	2.096895	0.504966	0.191189	1.474949	2.069817
0.702612	0.404083	1.26357	2.875467	1.158356	3.184402	0.263164	5.369783	5.923039
2.533429	1.007535	1.818483	2.160673	3.11812	0.511627	0.222233	2.457945	1.909962
6.518742	13.38637	6.941512	11.94055	0.368442	9.034708	2.587993	0.995003	0.358223
1	1	1	1	1	1	1	1	1
0.790413	0.833485	3.057674	3.998631	2.260601	2.740757	0.864771	1.417158	2.992795
5.462676	5.056893	2.431732	8.440507	0.470084	10.81428	0.297707	1.771932	0.508305
2.341935	8.422994	5.643901	99.81758	0.639495	10.48134	1.550888	0.826679	0.119323
2.214912	2.198676	1.220913	3.21253	0.385477	14.0149	1.693028	1.896112	0.861148
0.883944	0.434953	2.441348		2.450752	0.645475		0.965439	1.161019
2.069921	1.092876	2.83087		1.586332	0.684763		1.105132	1.446664
4.71723	27.29118	9.797876		0.821745	0.949835		0.897891	0.229697
4.96451	4.703511	2.985725		0.80783	8.935224		0.981796	1.720718
3.202709	42.51704	54.50823		2.162025	18.31135		1.597521	0.365248
1.263887	0.286825	1.29472		0.633221	1.724806		0.699659	0.51536
2.348018	9.349541	3.982086		1.053009	28.26558		0.918109	0.402149
1.47363	0.905331	2.082595		2.284876	2.057175		3.260729	1.227013
2.755046	2.12943	0.841852		1.929536	3.083704		1.948871	1.549573
1	1	1		1	1		1	1
0.859045	0.332758	1.107138		1.225165	1.207906		1.03426	0.964626
3.428874	3.228117	1.349159		0.214107	4.74748		0.345583	0.116182
4.257217	1.26082	1.591372	1.414307	0.186358	10.4118	0.299273	0.943677	0.558202
2.667636	1.72247	0.697055	3.909721	0.183751	3.623909		0.671104	0.14201
1.377998	0.297956	0.508596	0.852038	1.149163	1.346823		0.473142	0.967389
1.563399	0.142205	0.813592	0.839041	0.927541	1.150729	1.068913	0.911	0.751614
1.864517	1.050101	1.716285	0.829853	0.13638	6.248615	0.094132	0.621855	0.350159
5.488353	2.665186	3.306327	4.56893	0.575964	18.47513	0.350679	1.000571	0.722872
4.680559	3.632397	4.926854	30.46722	0.306627	22.23633	0.745844	0.764431	0.119283
5.333459	28.03888	19.69874	204.0951	2.867034	13.77944	4.5699	0.914133	0.332269
1.728513	2.199472	1.42493	2.848877	0.18603	4.768814	0.347293	0.44766	0.120114
3.047318	0.581513	0.964318	0.431397	0.085803	3.740818	0.099008	0.734755	0.183977
2.517502	3.804381	3.395985	3.374686	0.721737	28.243	0.340264	0.766421	0.277824
2.740837	0.383163	0.785846	0.131636	1.341615	2.022788	0.690432	0.929963	1.465874
0.803624	0.261391	0.597014	1.89714	0.812951	0.993945		0.862864	1.623966
6.445394	4.96014	10.24857	51.78265	0.472911	21.65685	0.892924	1.274981	0.206312
2.982497	0.92216	1.775433	1.831403	0.590243	8.521184	0.057867	1.642687	0.779684
5.828317	1.24341	1.371887	1.197362	0.388576	6.696792		1.133162	0.728816
0.639632	0.3825	1.229117	1.191837	1.07812	0.869014	0.93553	1.097695	1.330471

Liver_TFPI(	Liver_SELP	Liver_C5AR	Liver_VEGF	Liver_SERP	Liver_STAT	Liver_OASL	Liver_OAS2	Liver_OASL
1.555231	1.938008	0.617183	1.649269	0.645737	5.551837	1.597215	1.200502	0.962322
1.631156	22.85341	4.501502	0.979981	1.453843	3.171436	3.876841	1.469433	2.087435
0.825859	4.704176	1.05264	1.519001	0.464396	5.866918	1.492726	1.325698	0.98182
4.248457	16.00637	0.586359	0.605423	1.209479	4.378081	0.621837	0.765335	0.542621
1.090506	11.89314	0.474452	0.966482	0.624028	2.375815	2.841962	1.377099	2.228597
1.508197	99.17513	0.881251	0.979425	377.6404	14.50943	1.822459	1.573647	1.158385
1	1	1	1	1	1	1	1	1
1.182993	3.87348	3.269021	0.562024	0.461688	1.96401	0.593275	0.468714	1.993316
1.460172	34.16972	5.05118	0.639681	34.45815	2.850029	0.984027	1.319246	0.97734
0.620811	45.56931	3.388258	1.077878	93.86038	1.464143	3.436203	0.625025	0.549396
1.792516	17.85286	1.744432	0.331883	144.1111	0.679388	0.56087	0.128636	1.022981
1.226786	10.3092	3.966114	1.803538	1.002717	1.855669	1.537423	1.304752	0.941613
1.346029	28.77923	3.534613	3.765496	1.268492	0.471211	0.54971	2.386456	1.386587
1.590291	194.5141	23.47912	3.123806	533.1372	5.51846	11.10099	6.42885	8.768302
2.620519	162.2713	17.13605	4.553354	13.1486	5.524636	2.76455	10.86455	7.901655
2.235011	559.9715	38.87409	5.364702	388.6463	0.815741	10.96954	15.05092	9.375671
0.89643	2.540469	1.513848	1.606465	0.283184	0.803781	0.710554	0.277187	0.328828
1.893149	24.37068	6.33605	7.698779	200.3893	3.793117	2.969533	3.094437	6.743315
1.874061	2.781973	2.526572	2.058432	1.313763	1.580291	2.269369	0.333619	2.712994
1.704538	105.2351	2.326801	6.973347	63.18141	2.625589	0.276736	2.262625	0.534429
1	1	1	1	1	1	1	1	1
0.787879	1.723566	2.415241	4.14207	0.751299	1.082554	1.66426	0.703597	0.775877
0.509602	93.75013	13.14171	1.682729	150.1046	1.472204	0.585358	0.459959	2.298276
1.302085	17.93907	6.689709	0.285867	36.83418	0.939387	0.711162	1.129693	2.625408
0.829696	40.40751	10.36071	0.874983	122.5121	0.740323	0.350577	0.636691	1.53814
0.819188	0.820835	1.049582	1.169284	0.917747	0.846797	1.469405	0.480727	0.85117
0.572689	0.318613	0.828918	0.649072	0.829537	0.995165	0.760937	0.912565	0.86682
0.92353	4.115759	2.673475	0.648592	81.68667	0.795716	0.564558	0.887421	1.973558
1.418748	8.727269	17.21849	0.509279	133.6908	0.83964	1.030505	1.799255	3.102224
0.889312	4.692083	34.0166	0.152629	358.9577	1.14269	3.116919	2.524733	5.419451
0.716072	129.6149	50.7674	7.824396	1196.502	0.944297	15.7397	2.78221	3.322245
0.409478	1.350203	6.47975	0.234475	101.4608	0.517784	0.384001	0.554499	1.192548
0.775825	8.377826	8.529237	0.45708	21.15947	0.693228	0.339275	0.453957	1.020698
0.942271	26.39099	29.27612	0.340622	156.9959	0.963446	1.663697	1.841661	3.668341
1.309607	1.413929	17.94353	0.479553	0.744056	1.034528	0.937547	1.735964	1.491095
1.177126	2.330566	5.33475	0.302772	0.950248	1.395033	0.888602	0.807236	1.374283
1.677435	31.32296	23.90722	2.029022	1436.009	1.072626	1.768799	2.107011	3.468586
1.340191	11.13712	3.567325	1.904544	81.58867	1.785418	1.172735	1.692384	3.404986
1.307286	42.55202	19.6298	1.213226	200.7347	1.252875	0.67247	1.08895	2.013432
1.746147	3.138601	1.206392	1.54066	1.205492	1.004858	1.314169	1.095812	1.153642

Liver_OAS3	Liver_FOS	Liver_MX1	Liver_IL18	Liver_IRF1	Liver_IRF7	Liver_IRF8	Liver_CXCL	Liver_CXCL
4.561965	0.073312	0.76362	1.535785	1.346032	1.151529	1.062377	0.388727	1.941686
4.757501	0.228238	2.941793	1.018623	1.419049	1.356938	1.577505	1.029166	2.983807
1.910174	0.155917	1.275658	1.170777	1.150111	1.033764	1.426547	0.979183	1.588011
1.800803	0.106104	0.559134	1.088711	1.178477	1.113827	1.182652	0.686334	1.551298
6.103103	0.020547	1.253739	1.684811	0.928448	1.743231	2.001772	0.726405	2.73301
2.342576	0.007328	2.003785	0.203879	0.686115	1.644635	2.040646	13.348	133.9218
1	1	1	1	1	1	1	1	1
0.786727	0.02014	0.445992	1.123152	0.977514	0.854139	0.962288	1.321189	0.829501
3.3669	0.008115	0.954712	0.176804	0.384188	0.735083	0.976374	4.05931	22.59905
3.377123	0.00582	5.236301	0.175396	0.903353	1.365727	2.625131	17.05327	97.67908
7.038637	0.0022	1.262853	0.106113	0.385789	0.669852	0.626611	3.822233	61.55588
0.276456	3.768006	6.274638	2.30268	2.708647	3.236929	2.223397		0.416272
2.428359	13.07247	6.203561	2.936617	2.345142	2.431851	3.517584	8.935118	1.023729
3.064541	7.442003	73.33579	0.558008	6.04418	3.377623	13.90917	349.48	488.7991
5.216656	7.851577	8.2423	1.04211	2.785716	9.418532	1.393462	29.07643	96.34828
11.45391	17.84809	179.4998	0.978999	2.460322	7.777265	42.76096	2253.427	501.3947
0.469933	0.621298	3.047193	0.56195	0.909439	1.430031	5.503626	5.184725	0.562461
7.207356	5.195299	9.661462	0.263211	2.109616	2.598807	9.85449	249.8566	110.2935
0.995657	1.03986	2.367046	1.384093	1.02654	3.190989	4.102488	9.492562	53.4252
0.732954	33.18378	0.585405	0.460053	0.765752	0.313057	1.863817	9.062678	1.621585
1	1	1	1	1	1	1	1	1
0.394095	1.975534	3.187177	1.128561	1.392455	2.238107	2.20376	1.241897	0.595519
2.079832	2.047443	1.76302	0.171572	1.245725	1.499901	2.262523	82.29745	53.52184
5.151999	3.12437	1.172067	0.280131	0.546567	1.556349	1.279366	10.27426	130.5625
2.419586	1.326891	0.884113	0.088989	0.426205	1.206318	1.195426	12.13478	134.8918
0.706509	3.877772	1.313637	0.64341	0.459492	1.170617	0.978329	3.504211	0.664265
0.704556	0.691911	1.039492	0.843446	0.964534	0.914982	0.602342	1.132203	0.488681
2.612944	0.863247	1.064377	0.197746	0.352773	1.192724	1.00516	8.533117	40.44771
5.766941	2.867534	1.315059	0.377234	0.622693	1.488315	1.006486	18.58369	47.44248
12.04996	2.562396	9.68322	0.195292	0.913259	1.728805	2.662228	38.22076	101.201
8.294974	7.614987	72.22842	0.281314	1.693551	1.202158	5.01351	422.9397	534.5318
1.845524	0.797466	0.575037	0.155209	0.267307	0.607496	1.068801	12.33398	59.08374
1.160642	0.609042	0.774739	0.175314	0.33253	0.94805	0.622703	3.902234	50.51199
8.830395	2.086369	7.274775	0.290187	0.877628	2.000699	3.629742	27.74491	62.52677
3.081147	2.084965	1.44488	1.583965	1.320494	1.895607	2.277818	2.335797	2.241601
0.56448	0.948179	1.521603	1.008914	1.279083	1.357602	0.815914	2.64243	1.120156
6.241457	6.622789	5.18682	0.303705	1.169275	1.743082	5.331964	128.062	151.6876
5.60154	1.53839	2.414828	0.299664	0.994354	2.33828	1.989575	19.92399	87.12362
3.704668	1.240149	1.55157	0.249145	0.654257	1.549381	1.02442	13.65099	74.43902
1.419334	1.445272	0.962008	1.185613	1.03677	1.092917	1.660185	0.883234	2.046325

Liver_IFIH1	Liver_DDX	Liver_KLF2	Liver_HNF1
1.407919	1.238724	0.909539	1.05739
1.447384	1.404987	0.880276	1.259822
1.35081	1.870749	3.427519	1.288668
1.216836	1.072773	0.737294	0.787958
1.82272	2.006452	2.176902	1.507968
0.533641	0.542672	1.831985	0.91133
1	1	1	1
0.922347	1.135824	0.971922	0.849919
0.293966	0.381305	0.748178	0.621281
0.542544	0.391232	10.23667	0.63476
0.302831	0.337728	2.977163	0.353006
0.966616	0.859037	3.273839	2.562631
0.998053	1.285024	2.880546	1.663199
1.428671	1.140455	4.050856	1.831064
0.762935	1.467435	2.39222	2.105832
1.62555	0.909296	9.502708	2.168841
0.862711	0.617206	0.321588	0.619223
0.547277	0.610517	3.512133	1.742708
1.165027	1.316728	1.363529	1.266497
0.426058	0.620286	1.577947	1.192167
1	1	1	1
1.020528	1.071316	0.994239	2.099874
0.246212	0.247538	1.048564	1.360049
0.593841	0.888622	0.997219	0.653571
0.336015	0.609406	1.026397	0.766231
0.826929	1.64158	1.175846	1.292318
0.938891	0.58242	0.439121	0.629095
0.437439	0.383464	1.62814	0.273309
0.501329	0.673539	1.734863	0.714958
0.936579	0.527358	2.301555	0.422349
1.573088	2.328082	8.036089	0.224717
0.318589	0.342923	1.18745	0.569503
0.523348	0.395953	0.791079	0.379035
0.614405	0.594651	1.74727	0.665762
1.275806	1.744142	1.149437	1.290505
1.362586	0.716858	0.341969	0.851684
0.839301	0.783682	3.463626	1.538454
0.901741	1.531206	0.978531	0.977878
0.588796	0.780075	1.045923	1.02817
1.065086	1.716974	2.277278	1.589584

Supplemental Table 7

Mouse ID	Gender	Genotype	Tx	Group	KIDNEY-ICA	Kidney_SEL	Kidney_IL6	Kidney_SO
5129	F	fl/+	Saline	Saline_fl/+	-0.03765	1.512478	-1.21941	-0.1245
5130	F	fl/fl	Saline	Saline_fl/fl	0.273184	3.278635	-0.04319	0.648275
5132	M	fl/fl	Saline	Saline_fl/fl	-0.36346	1.22282	0.001518	0.584846
5133	F	fl/fl	Saline	Saline_fl/fl	0.477024	3.616386	0.273273	1.123116
5135	M	fl/fl	Saline	Saline_fl/fl	0.586155	1.983688	0.840523	1.257746
5137	M	fl/fl	LPS	LPS_fl/fl	2.199444	5.886215	7.37064	5.386599
5139	M	+/+	Saline	Saline_+/+	0	0	0	0
5140	F	+/+	LPS	LPS_+/+	3.119209	5.318974	5.511379	4.755705
5142	F	fl/+	Saline	Saline_fl/+	0.032154	0.986221	0.17482	0.820221
5143	F	fl/+	LPS	LPS_fl/+	3.409996	5.939266	7.925144	6.260839
5144	M	fl/+	LPS	LPS_fl/+	3.053694	5.15844	5.185793	4.307207
5150	M	fl/fl	Saline	Saline_fl/fl	0.503309	-0.16714	0.190874	0.247366
5151	F	fl/fl	Saline	Saline_fl/fl	0.435799	2.399986	-2.34808	0.862123
5155	F	fl/fl	LPS	LPS_fl/fl	5.542513	3.656151	10.30392	5.951374
5156	F	fl/fl	LPS	LPS_fl/fl	1.185415	1.325159	6.136724	0.085371
5178	M	fl/fl	LPS	LPS_fl/fl	3.70586	-1.91824	-4.45667	3.646122
5179	M	fl/fl	Saline	Saline_fl/fl	0.237814	-0.8165	-0.50199	-0.65733
5180	F	+/+	LPS	LPS_+/+	4.106718	2.47019	8.263233	5.526072
5181	M	fl/+	LPS	LPS_fl/+	0.727171	-1.64733	0.483423	-1.00379
5182	F	fl/fl	Saline	Saline_fl/fl	1.645779	1.877659	5.350048	2.339405
5183	F	+/+	Saline	Saline_+/+	0	0	0	0
5184	M	fl/+	Saline	Saline_fl/+	-0.24056	-1.32079	0.618589	-0.60459
5185	M	fl/+	LPS	LPS_fl/+	3.655201	2.007597	6.807116	4.199827
5219	M	+/+	LPS	LPS_+/+	1.987885	0.421926	6.160135	2.30143
5220	M	fl/+	LPS	LPS_fl/+	2.92169	-1.1942	4.289671	3.666336
5221	M	fl/+	saline	Saline_fl/+	-0.71905	-0.64039	-1.99018	-1.7966
5226	M	+/+	saline	Saline_+/+	0.171061	1.216911	1.175728	-1.16616
5228	M	fl/+	LPS	LPS_fl/+	2.416086	0.179337	3.940735	1.329351
5230	F	+/+	LPS	LPS_+/+	3.009216	2.600588	7.751316	3.381695
5231	F	fl/+	LPS	LPS_fl/+	3.612831	1.844479	7.94656	5.573807
5232	F	fl/fl	LPS	LPS_fl/fl	4.731182	0.936483	11.13372	5.020716
5233	M	fl/+	LPS	LPS_fl/+	1.782167	3.476553	7.854252	6.546446
5234	M	+/+	LPS	LPS_+/+	2.178543	-1.3736	3.85087	4.258171
5239	F	fl/+	LPS	LPS_fl/+	2.924772	1.599678	7.444796	3.845966
5240	F	fl/+	saline	Saline_fl/+	-0.90178	0.090801	-1.20319	0.654224
5241	M	fl/+	saline	Saline_fl/+	-1.45335	-1.13292	-0.24692	-0.73625
5242	M	fl/fl	LPS	LPS_fl/fl	3.171242	3.849104	9.063236	7.326674
5243	M	+/+	LPS	LPS_+/+	1.847694	0.522917	4.659294	1.41781
5244	M	+/+	LPS	LPS_+/+	3.395941	0.42944	4.004831	4.305792
5245	F	+/+	saline	Saline_+/+	-0.17106	-1.21691	-1.17573	1.166161

Kidney_ST/	Kidney_CEI	Kidney_F3	Kidney_RH	Kidney_TSI	Kidney_PL/	Kidney_PL/	Kidney_THI	Kidney_PL/
0.413256	2.021477	0.080416	0.331924	0.284239	1.497627	-0.62242	0.356752	0.265501
0.527964	1.92557	0.188105	0.024296	-0.586	0.88802	-0.10782	0.151585	0.230469
0.437485	1.753754	1.236629	0.441154	0.911757	0.681757	-0.15478	0.302197	1.047491
0.607613	1.96587	0.121166	0.298685	0.514341	1.191359	0.311417	0.651281	0.943636
0.595253	1.341629	0.951141	0.908234	0.84901	0.949162	-0.06425	0.456375	1.478249
2.44733	2.796463	0.692451	1.634478	-1.34747	0.760042	-1.52591	0.239466	3.820204
0	0	0	0	0	0	0	0	0
1.844664	3.285748	-1.31165	1.466314	-1.00763	1.530502	-3.13278	-0.60684	2.937141
0.636744	1.99366	-0.65008	0.675442	0.320576	0.764032	0.116135	0.004852	0.527769
2.788998	3.863279	0.218891	2.568338	-1.00061	1.499716	-3.35971	0.354807	4.037895
1.71509	2.276833	-0.27192	0.974154	-1.10272	0.736139	-2.21375	-0.28644	2.055252
-0.59893	-1.90018	0.776716	-1.18264	-0.62959	-1.27056	-0.78229	-0.6775	-0.02508
-8.0304	-5.73028	-3.68177	-4.99992	-3.34303	-0.0589	-0.82259	0.54055	1.302235
2.536985	1.104475	3.615705	1.847544	-0.6757	0.597799	-2.87723	-0.05663	3.705435
-1.20977	-2.57913	0.759039	-1.99416	-2.72882	-1.76833	-7.81576	-2.49621	0.206043
-14.5811		4.794668		-0.99694	-0.53681	-2.33842	-0.111	4.170544
-1.13468	-1.35509	0.559435	-1.48657	-0.38909	-0.60408	-0.28019	-0.09	-0.02791
1.664862	1.434519	-0.42929	1.401596	-0.75887	0.342737	-2.25386	0.613098	3.603765
-0.77059	-1.28374	1.015738	-1.53384	-0.50947	-0.51683	-0.24107	0.008781	-0.29678
0.837128	3.497843	0.999531	0.526325	0.789858	-0.49237	-1.2045	0.645342	1.369164
0	0	0	0	0	0	0	0	0
-1.27629	-1.54936	-0.42738	-1.86932	-0.77297	-1.30205	-1.83539	-0.93914	0.438658
1.018963	-0.02004	-0.05059	0.504993	-1.44002	-0.08323	-2.91546	-0.12329	2.360746
1.645535	0.522837	1.875378	0.699249	0.022906	-0.64488	-0.57201	-0.72055	0.095876
0.999838	-0.49216	0.457924	0.310009	-1.4505	-1.12085	-3.79955	-0.45385	0.327506
0.622553	-3.11135	0.532564	0.492699	0.89891	-1.81646	-0.93979	0.108649	-0.41733
-0.54798	-0.63544	0.029171	-0.44987	-0.42888	-0.2757	0.646337	-0.45188	-0.28478
3.289932	0.769547	0.579939	0.622349	-0.78512	0.295347	-1.82964	-0.79089	-0.82514
1.67395	3.62833	0.415586	1.481956	-0.33812	0.579952	-2.20989	-0.28205	2.091361
3.01446	0.871822	0.033798	2.376608	0.067426	-0.63482	-3.90252	1.295205	3.686418
2.635809	0.932882	4.188005	2.649986	0.397351	-0.14665	-2.5264	0.510968	3.045106
1.580881	1.130157	1.096926	2.170589	0.389531	1.486853	-3.4366	0.56845	3.567956
2.96698	-0.57614	0.887262	0.952404	-0.58189	0.511724	-1.65419	0.324347	1.871715
2.153429	2.987381	-0.66494	1.85956	-1.06843	0.860699	-3.36784	-0.20248	4.049333
-0.02145	1.948441	-0.15493	-0.92043	0.605399	0.784803	0.575549	0.252595	0.250375
-0.6033	-1.80206	-0.05322	-1.78205	-0.67939	-1.18503	-1.78073	-1.74419	-1.63385
2.580528	1.213202	3.434364	2.800911	1.022819	0.897509	-1.36253	1.135121	5.476956
1.773245	-1.05686	2.009432	0.25028	0.263457	0.213734	-2.97743	-0.93685	-0.14021
1.57234	0.857788	2.060564	1.976007	0.854777	1.203686	-2.72952	0.158259	0.471681
0.547979	0.635445	-0.02917	0.449869	0.428876	0.275696	-0.64634	0.451882	0.284781

Kidney_SYI	Kidney_ET1	Kidney_PT(	Kidney_VW	Kidney_PR(	Kidney_CD	Kidney_TFF	Kidney_TFF	Kidney_TFF
-0.11799	1.098648	0.694284	1.839319	-0.12833	2.508963	3.458002	3.195765	1.955925
1.959433	-8.17899	-0.53581	1.600193	0.03553	1.296522	2.543619	2.226002	1.719997
0.121876	0.686039	4.333078	3.686476	4.209932	5.971403	3.774944	4.065235	2.922657
1.082973	0.858147	0.950735	1.889278	-0.18727	2.953714	2.787489	3.277943	1.456287
0.341206	0.305475	1.966753	2.189976	1.118441	3.441496	1.428135	1.67737	1.546968
-0.89701	2.349737	4.079424	0.609631	6.458187	0.910482	3.702003	2.87142	2.429276
0	0	0	0	0	0	0	0	0
-0.73948	2.298441	0.425575	-1.97705	-0.00015	-0.04161	-0.13684	-2.58393	-1.93966
0.628269	0.359652	0.200546	1.679304	0.970884	1.59737	2.513094	4.251692	2.255867
-1.46037	3.317717	6.122181	1.4723	5.614824	4.442482	2.906609	0.04731	3.22703
-0.74642	1.584549							
-1.22812	-0.25894	-0.16411	0.204453	-1.26776	-0.38275	-0.88262	-0.64078	-0.80944
0.527252	2.45459	1.138378	1.516037	0.329704	1.204403	0.497995	-0.34127	0.016096
-2.25225	3.190521	3.90173	-0.86506	1.247932	0.766722	-1.58732	-3.83748	-1.17157
-5.69592	1.32585	-1.71306	-4.09555	-0.35825	0.434275	-1.30366		
-4.4718	2.617243	3.280508	-2.55021	0.774609	-2.31766	-2.57133	-5.99958	-2.08574
-1.04916	0.141844	-0.63155	-0.04057	-0.42078	-2.5321	-0.9081	-0.77295	-0.99463
-1.4415	3.56064	3.751837	-1.46452	-0.08399	1.264677	-0.53786	-3.10418	-0.29178
-1.07881	-0.28477	-0.8834	-0.88159	-0.61687	-2.19066	-0.67979	-1.58451	-1.19213
0.41547	1.470829	1.369678	1.801212	2.164997	2.024204	0.118845	-0.81634	-0.20222
0	0	0	0	0	0	0	0	0
-0.33842	-0.43918	-0.04021	-0.357	-0.52871	-0.82948	-0.60813	-1.43171	-0.93785
-1.10625	2.55423	2.079422	-1.65622	0.354496	1.706991	-2.07234	-3.05839	-1.22067
-0.47919	1.183988	-2.6475	1.687104	-5.35966	-0.24573	-2.14947	-2.71482	-0.15692
-0.88756	1.49433	1.865753	0.627851	-3.59762	-2.48517	-1.8139	-4.02765	-0.09044
-0.35119	-1.02923	-0.74141	2.674844	-3.14289	-1.7619	-0.70552	0.655685	1.718821
-0.03837	0.530597	-0.19765	2.395616	-1.86458	2.224889	-0.83076	0	-2.4355
-0.84658	2.091851	-0.66543	0.002284	-4.8321	-1.82591	-0.48198	-1.73387	1.337299
0.041113	2.112412	2.527403	1.921807	-2.37757	0.326459	0.323912	-0.48576	1.277599
-0.69781	2.366939	3.83499	1.339618	-3.65731	-1.54928	-0.11467	-4.15382	1.06715
-2.51882	1.801953	5.491459	1.915185	-1.48845	2.793565	0.451406	-1.38487	0.937572
-0.87391	1.074521	1.943472	-0.03186	-2.58696	1.238059	-0.3305	-3.24963	-1.06957
-0.22886	1.704713	0.533918	1.328115	-2.39606	-0.37577	-2.08475	-1.78691	1.101501
-0.97206	1.985732	-0.06033	1.592652	-2.84822	-0.10481	-2.57612	-4.26134	0.043417
0.700676	-0.58297	-0.92126	3.781127	-3.35996	-0.19146	0.832487	-0.97866	0.974722
-0.4769	-1.04158	-0.83823	2.225814	-4.84028	-3.57465	-0.07711	-1.67291	0.172991
-1.22367	2.426576	5.174128	1.200456	0.583572	2.201949	-1.41494	-3.30374	1.462576
-1.80513	1.116328	-0.3921	2.10971	-3.01001	-2.8553	-0.71996	-2.43234	-0.04452
0.310436	2.666726	1.578192	0.854098	-2.15079	-1.45026	-0.03341	-1.07795	0.282244
0.038368	-0.5306	0.197651	-2.39562	-4.04878	-2.22489	0.830765		2.435503

Kidney_SEI	Kidney_C5/	Kidney_VE(	Kidney_SEF	Kidney_ST/	Kidney_OA	Kidney_OA	Kidney_OA	Kidney_OA
1.269251	2.162123	2.916624	-0.3482	-0.04277	0.438843	0.728905	0.175632	1.938715
2.356091	2.26005	2.520353	-0.42876	0.495373	0.359022	1.297119	0.542112	1.274065
4.638296	4.849234	6.824125	3.507164	3.227791	1.718117	4.367758	3.350674	2.920351
2.292814	3.317087	2.906809	0.635353	0.742064	1.576694	2.022707	1.173035	3.626066
1.92415	4.883738	3.831381	2.523823	1.112734	1.614653	2.53714	2.132542	5.670525
6.434731	5.538319	4.228794	5.937637	4.487949	2.609201	4.628534	4.967222	6.022429
0	0	0	0	0	0	0	0	0
3.86376	1.861258	0.940973	2.010941	1.075802	1.576622	1.434242	2.525763	4.005497
0.553436	3.104691	4.352018	0.259508	1.962849	0.59874	1.259262	1.04278	0.943504
8.214859	3.953663	1.94389	6.187994	5.557039	6.383612	4.791891	4.371147	6.390137
1.196426	0.423565	0.796825	-0.2116	-6.09826	0.154839	-1.20528	-0.54749	-1.73708
1.916176	0.739208	1.395432	-0.33458	0.500317	0.705044	-0.6075	1.807709	-0.24557
7.709177	1.608521	-0.60858	3.019424	2.165882	5.735958	1.629871	3.107082	2.212635
7.049353	1.23769	0.182196	-3.39279	1.609356	1.747765	1.36553	1.826096	1.947975
7.669436	1.219425	-1.86286	4.180803	2.304087	5.505375	-0.61533	2.6075	3.320885
1.267834	2.323545	0.85461	-0.38218	-0.18592	1.742373	-0.44146	-1.0801	0.231892
6.260258	1.123894	-0.78649	4.047905	3.225163	5.492216	0.475216	1.950716	2.133354
-0.01217	0.655165	0.154879	-1.27036	0.022087	3.634647	1.562593	0.237196	2.116287
5.970818	1.845266	0.377245	2.195366	-0.25444	0.711288	-0.44759	-0.34711	-0.30494
0	0	0	0	0	0	0	0	0
0.915207	2.144444	0.605507	0.136076	-1.49093	0.431236	-0.90766	-0.03354	1.499664
5.632404	1.11552	-1.06618	5.085316	0.924652	3.139143	2.028492	3.002596	1.392542
2.587812	-0.03721	-2.16401	-1.16507	1.469695	2.440596	1.884672	2.431425	2.551105
1.343655	-2.73201	-2.69841	-0.26397	1.094466	2.602821	1.12351	2.064598	1.706432
-0.84256	-2.27068	-1.46252	-3.40099	-0.80486	-0.56341	-0.37547	-0.49549	-0.21651
0.171539	0.615724	-1.04755	-0.04829	-0.2712	0.144461	-0.05309	-0.07633	0.049429
1.307526	0.410295	-0.87065	0.052138	1.142447	2.72049	1.513788	1.701529	1.842653
4.461082	1.181312	-0.35057	-0.30071	0.887322	2.322741	1.761278	2.062458	2.148258
1.58992	-0.78314	-0.56509	1.091979	2.889592	5.795471	2.548849	3.72097	3.372805
3.678185	-0.20071	-1.21354	1.24611	2.784446	7.461332	1.787138	1.969181	2.891258
2.810345	-0.71795	-1.2754	1.226413	0.980828	3.429274	1.595837		1.764128
0.860819	0.227773	-1.69134	1.240932	1.250468	2.148212	1.247355	1.838499	2.111542
3.101774	-1.9875	-1.86495	2.718581	0.92615	2.881189	1.028048	1.877747	0.75038
-0.76259	-1.4467	-0.65012	-0.76153	0.389722	0.247622	0.554354	-0.15893	-0.08505
-1.14612	-0.38131	-0.20967	-2.96068	-1.21771	-1.32011	-1.11728	-1.36878	-1.39089
6.09882	-1.14652	-1.44523	1.499356	1.944638	4.077269	2.20126	2.701912	3.396797
3.880249	1.361508	-2.19552	1.785201	1.571364	3.06757	2.263132	2.075977	1.721107
3.445757	-0.09212	-0.28525	0.970349	1.315553	2.821898	1.927843	1.788464	2.3617
-0.17154	-0.61572	1.047545	0.048293	0.271195	-0.14446	0.053089	0.076332	-0.04943

Kidney_FO	Kidney_MX	Kidney_IL1	Kidney_IRF	Kidney_IRF	Kidney_IRF	Kidney_CX	Kidney_CX	Kidney_IFIH
0.298452	2.570082	0.182848	1.848822	0.717697	1.094612	0.873583	2.171083	1.717949
-0.26877	2.359655	-1.19579	0.754061	0.791164	0.293289	0.336073	0.075163	0.396484
3.638697	4.797007	2.014683	3.275902	3.004833	3.496931	3.818644	2.415205	3.360935
1.394842	3.061047	-0.71922	2.217489	1.976543	0.476328	3.257114	1.386934	2.146269
0.787605	3.910963	0.099592	0.536741	1.632708	0.59618	3.017843	3.039793	0.648031
1.183563	8.559776	2.464842	3.948492	7.014448	4.512053	8.357834	9.506042	3.824659
0	0	0	0	0	0	0	0	0
-2.47495	3.735092	-1.47817	0.9182	2.643463	-0.00492	3.696739	6.470341	0.417627
-0.64635	3.294722	0.534691	1.770451	1.444733	1.206518	1.67329	1.97229	1.157633
2.32346	9.263176	1.494062	5.206284	6.094528	4.644545	8.072556	10.26512	4.492039
3.735765	0.153839	0.812017	0.12063	-0.90881	-1.10371	3.408899	1.55999	0.275879
4.177067	0.631598	1.495907	0.446802	0.479361	-0.20021	1.144695	1.41713	1.789824
7.98543	8.994232	1.105158	2.099625	3.183851	2.381397	7.032499	9.987009	3.737276
3.070585	2.756538	0.649282	0.531588	4.016499	-1.63957	2.743534	7.156952	2.68771
5.682774	5.84186	2.092665	2.599316	4.008234	4.788557	10.50824	8.014919	3.243635
4.288481	1.537804	0.499994	-1.2073	-1.03751	0.57888	0.492765	0.509018	1.821304
4.78582	4.371946	2.326273	1.956059	4.826448	0.465773	5.129948	8.037325	4.469013
3.227028	1.508022	2.303328	-0.92619	4.620094	-0.83641	0.373892	5.700432	2.049212
5.460192	-0.11485	1.222652	0.161264	-0.31966	-0.28451	1.997168	1.938848	1.04418
0	0	0	0	0	0	0	0	0
4.681795	-0.82122	1.327883	-0.53462	0.619228	-0.07347	-0.90089	-0.35289	2.574493
3.60442	4.424936	-0.04522	1.147696	3.008404	1.339462	6.524305	5.750174	3.148706
2.07525	2.336477	-0.42202	0.516919	3.501933	-0.5087	1.608172	4.923328	-0.42685
1.198663	3.003244	-0.84838	0.836532	4.036094	0.869154	2.664727	3.648567	0.460917
1.432927	-0.18129	-0.45327	0.467515	-0.39287	0.244425	-2.8757	-0.26134	-1.24807
-0.66936	-0.64479	-0.92698	0.33814	0.620673	-0.69125	0.574126	0.205803	-1.31493
-0.30717	1.886244	-0.41181	0.574832	4.577336	0.726807	0.230178	3.636652	0.276027
1.411847	1.972559	-0.88571	0.717629	3.441855	-0.60582	2.924882	5.427254	-0.81272
2.214725	5.972178	-0.41761	2.031661	3.365907	0.772184	5.813878	8.329351	1.397849
5.031491	7.573597	-0.88304	3.416818	2.340907	2.787169	5.520492	7.383717	2.325225
1.371409	3.128849	-0.45276	-0.25393	2.171704	1.220955	3.524102	5.671089	0.576745
0.224675	1.799675	-0.48046	0.342147	3.214063	0.299639	1.966641	5.334438	-0.42993
1.723029	5.097507	-0.4262	2.197806	3.795993	1.014641	2.936933	6.163729	0.746707
-0.12767	0.920774	0.298008	1.741147	0.841353	1.189089	-1.91039	-0.07308	0.714053
-1.23394	-0.08789	0.315165	0.819898	0.058343	0.212763	-2.59248	1.636885	0.076398
3.311284	5.439823	0.925106	2.303782	2.931239	2.740929	6.545203	8.281506	1.72092
1.521827	3.04982	0.056765	0.89849	3.842406	-0.01759	1.230109	4.969284	0.338748
1.62654	3.024372	0.028542	1.180022	3.638764	0.636646	2.965178	5.795015	1.233079
0.669356	0.644794	0.926979	1.777587	-0.62067	0.691254	-0.57413	-0.2058	1.314927

Kidney_CL	Kidney_HA	Kidney_LC	Kidney_DD	Kidney_KL	Lung_ICA	Lung_SELE	Lung_IL6	Lung_SOCS
1.757071	-2.03746	2.378557	2.361448	0.489429	-0.68714	-1.50876	0.204306	-0.82252
0.510906	-1.69005	3.423845	1.4562	-0.36991	-0.61917	-1.44511	0.016329	0.060081
3.381325	4.49305	4.824846	3.931494	3.627354	-0.75062	-0.95399	-0.16222	0.740208
2.69409	-0.8035	2.406004	2.288696	0.996758	0.479399	-1.80853	-0.02301	-0.50557
0.074099	1.288136	3.457289	1.737391	0.919004	-0.20292	-1.95278	-1.34961	0.029417
6.287144	9.198212	15.01878	4.168976	1.542328	1.243162	3.713293	7.365181	3.36043
0	0	0	0	0	0	0	0	0
2.817207	3.914713	13.49524	1.413816	-1.16079	3.510906	5.182539	6.67178	2.761604
2.986734	-2.44232	3.22472	2.091066	0.417315	0.028225	-0.97596	-0.30757	-0.51032
6.486385	5.242096	15.86073	4.604225	1.627916	1.441753	3.319386	8.352955	4.011662
					2.728823	4.359463	6.157642	2.626589
1.065985	1.380398	-1.53243	-0.7556	-1.41761	-0.06133	1.100473	-1.21055	0.520287
1.953306	-0.1572	-0.41642	-0.22506	-0.22244	0.20665	0.082458	-0.50044	0.482883
2.370632	3.470436	11.41995	3.852043	-0.2945	2.86767	4.216791	8.018751	5.196012
3.380064	5.458778	8.194359	0.94537	-0.79039	4.926825	0.788792	2.818794	
3.020029	7.453047	10.74889	2.080128	-0.28526	1.012299	5.295628	9.856358	4.30744
1.094109	2.160469	-2.04527	0.328133	-0.71921	-0.07698	1.056026	-0.08562	0.484241
3.013639	5.304905	12.65423	2.642202	0.482241	2.422859	4.977522	7.746126	3.591244
0.470886	3.288378	2.888155	1.313278	-1.34259	0.899441	0.231697	0.224194	0.563925
2.592793	3.525087	6.887873	-0.29814	0.084646	0.066555	1.91721	0.345264	1.708279
0	0	0	0	0	0	0	0	0
1.61105	2.937048	0.379044	-0.24475	0.353203	1.234756	2.85001	0.929882	1.795898
3.885937	7.094698	11.25391	0.939964	0.217281	1.234756	2.85001	6.68948	1.795898
-1.66268	5.352796	7.832028	-0.22812	-0.86048	2.021196	5.683111	7.189274	2.613345
0.017148	5.793424	8.064004	1.280437	-1.09461	1.168585	5.206066	6.595139	3.178481
-2.36914	1.565579	-2.99107	0.638259	-0.51839	0.225922	0.753672	1.150141	1.706304
-2.10899	-0.15383	-0.9176	0.489734	-0.50726	-0.03896	0.5905	-0.09459	0.495494
0.768128	5.223773	8.533387	-0.5885	-1.14078	2.488873	6.650274	5.761091	1.842364
1.439263	4.432321	9.257689	-1.22675	-1.37018	1.65662	4.327301	6.405022	1.99615
0.597407	1.845489	8.845271	1.616508	0.455905	1.187998	4.331285	8.388226	3.499438
1.726069	0.29263	8.660691	2.37637	0.38309	1.808041	5.894041	12.31264	5.029502
1.77381	6.351104	8.796184	-0.03313	-0.67879	1.299618	5.176006	6.385067	2.273339
1.170096	6.183835	9.377906	0.200683	-1.94986	2.45756	5.697594	6.259157	2.863194
2.90722	5.376613	11.00156	1.115617	-1.26416	2.502142	5.192753	7.756954	4.494531
-0.60467	-0.9832	-0.38228	1.748078	0.461223	-0.63189	0.300316	-0.74773	0.694575
-0.5709	2.784495	-0.28403	-0.67322	-1.13059	-0.25553	-0.03675	-0.1955	0.29742
2.857985	7.611072	10.64488	0.531438	-2.00253	0.459423	3.433197	9.834047	4.056365
0.962409	6.805753	9.181832	0.833747	-1.91166	1.812653	4.563999	4.568018	2.525073
1.69774	7.525397	9.544759	0.046445	-0.73306	1.727343	5.354656	7.029053	2.612523
2.108994	0.153829	0.917596	0.588504	0.507257	-1.188	-0.5905	0.094593	-0.49549

Lung_STAT	Lung_CEMI	Lung_F3	Lung_RHO(	Lung_TSP1	Lung_PLAT	Lung_PLAU	Lung_THBD	Lung_PLAU
-0.51303	-1.04048	0.011799	-1.21777	-0.86705	-0.39313	-0.03259	-0.532	-0.67819
0.784105	-0.44266	0.007669	1.054527	-0.26782	0.113747	-0.08546	-0.14759	-0.14455
-0.17619	0.129221	0.958202	-0.53259	0.185009	0.446455	-0.70843	-0.18451	0.135521
0.236818	-0.58404	-0.0251	0.325029	0.016483	-0.2438	-0.64414	-0.79063	-0.82799
0.321852	-0.96663	-0.68232	-0.18958	-0.80285	0.412439	-1.09	0.554453	0.315174
1.936457	4.427279	0.970203	1.82408	-0.2372	2.799347	-2.94683	-2.78369	2.65287
0	0	0	0	0	0	0	0	0
0.369402	4.250795	-0.0302	0.484602	-0.69469	2.680826	-0.7534	-2.63347	1.391054
-0.84673	-0.3743	-0.18903	-0.30423	-0.18911	-0.73497	-1.53819	-0.80877	-1.14342
1.290501	4.005737	0.236967	1.901178	-0.54757	2.715126	-2.12717	-2.9168	1.748262
0.447203	3.818916	0.1486	0.880281	-0.63651	3.173239	-3.30982	-2.26648	0.984423
-0.80672	-1.01861	-0.81252	-0.73119	-0.11635	-0.95758	-1.08069	-0.45581	-0.70846
0.115849	-0.00041	-0.65718	-0.08854	0.888885	-0.24652	-0.299	0.437859	0.066486
2.087835	3.928976	0.053917	2.596869	1.545248	2.253874	-1.15507	-2.79553	2.920994
		-1.73866	-1.09331	0.619045	1.056835	-1.42034	-2.04693	0.288307
1.539265	3.942833	0.764925	1.803627	2.356943	1.785736	-1.68516	-2.49492	3.260075
0.021162	-0.76459	-0.5348	-0.30973	0.274252	0.016911	0.029358	0.142355	-0.44252
0.994858	5.055891	-0.3698	0.766964	-0.00966	2.686348	-0.66326	-2.38802	2.214058
0.819851	0.100994	-0.4645	0.338408	0.177555	0.102636	1.379364	0.879654	0.512617
1.015961	1.224075	-0.61025	0.775684	1.436459	0.741722	-2.04996	-0.44756	0.57234
0	0	0	0	0	0	0	0	0
0.139164	0.663322	-0.22843	0.219658	0.760326	0.089741	0.375034	0.125143	-0.09308
1.283007	3.89146	-0.43203	0.89542	-0.28599	3.124443	-0.45738	-2.06985	1.789692
1.511287	3.666103	-0.21962	1.477282	-0.24882	2.578872	-0.211	-1.07567	1.111712
1.222436	3.869551	0.439362	1.805384	-0.0421	2.890922	-1.40895	-1.95249	1.999269
0.314351	1.369469	0.30422	0.811558	1.325111	-0.70423	0.082566	-0.05174	0.887848
0.645082	-0.97948	1.028904	0.863958	0.147179	-0.37852	0.347781	1.0622	-0.04268
1.269015	4.406046	0.910276	1.751255	0.810862	3.994925	-0.79327	-1.11824	2.205053
0.800366	3.174648	0.012178	1.155012	-0.68688	2.854224	-1.17884	-2.06902	1.188776
1.226539	4.594278	0.277498	1.411217	-0.12139	3.884271	-1.63865	-1.86961	2.480869
2.429957	5.725391	1.970886	3.853239	2.902803	3.686334	-0.79926	-1.44224	4.056137
0.230706	2.860468	-0.04477	0.207571	-0.7505	3.482285	-1.71254	-2.15991	2.094957
1.454148	3.307335	0.91802	0.823383	-0.07635	3.310376	0.210916	-1.16567	2.046082
2.03194	5.684864	2.053936	1.911196	0.419283	4.600175	-0.17948	-1.35328	3.138992
0.550071	-0.35944	0.975042	0.694309	0.330837	2.14956	0.420096	0.998099	0.788137
0.33895	-0.16775	0.546862	-0.24532	-0.45974	1.709382	-0.78238	0.308431	0.352381
2.325112	4.955278	2.282408	1.944246	1.046949	3.415317	-1.08928	-2.77069	3.434959
1.229407	4.189312	0.338455	0.6115	0.811377	3.015821	-1.3626	-0.98838	1.49122
0.646678	4.7164	-0.27512	0.640079	0.725328	2.979566	-1.84352	-1.32501	1.752592
-0.64508	0.979475	-1.0289	-0.86396	-0.14718	0.378522	-0.34778	-1.0622	0.042677

Lung_PTGS	Lung_VWF	Lung_PRO(	Lung_CD44	Lung_TFPIA	Lung_TFPIB	Lung_TFPI(	Lung_SELP	Lung_C5AR
0.066343	-0.30875	0.662714	0.18799	0.573853	0.48921	0.612982	-1.6524	-1.38344
0.689711	1.034357	0.711302	-0.14554	0.780048	0.354557	0.657555	0.658291	-1.05216
0.869833	-0.2571	0.340195	0.940752	0.113155	0.201492	0.004425	0.581978	2.289011
1.391691	1.093735	0.445307	-0.4509	0.831066	0.596565	0.501402	1.984949	-0.10718
0.154221	0.700768	0.042168	-0.6853	0.691109	0.63402	0.054382	-0.11165	-0.21742
1.538973	-2.76149	3.751825	0.579416	0.123928	-1.81126	0.433792	6.628151	2.925356
0	0	0	0	0	0	0	0	0
3.15675	-1.7523	2.90612	-0.29273	-0.42763	-2.22748	-0.37578	5.452446	2.902052
0.92651	0.321123	-0.11281	0.866581	-0.08209	-0.21033	-0.33698	-0.06801	-0.00963
1.457428	-2.60995	2.54472	-0.01081	-0.38913	-3.26611	-0.06496	5.644602	2.759741
1.835438	-2.08575	2.496641	-0.16373	-0.21961	-1.62371	-0.40316	4.130268	3.24749
0.633482	-0.05759		-0.1007	0.299458	0.226797	-0.01024	0.967531	0.014708
0.086422	-0.25659		0.277033	0.457756	0.211803	-0.32771	0.867434	-0.63632
1.46615	-1.54161		0.80056	-0.79005	-2.34153	-0.56349	5.791674	3.643564
0.265606	-1.62831		-0.63953	-0.05073	-0.71282	-0.62263	4.626442	0.779114
0.617413	-2.61956		0.994555	-0.78767	-2.91483	-0.86444	6.7316	3.060442
-0.02176	-0.24668		-0.39298	0.081514	-0.03861	-0.03226	1.561321	-0.80377
2.853832	-0.87117		0.114199	-0.62868	-2.49693	-0.70733	4.553501	2.852236
0.98444	0.91699		-0.68532	-0.29265	-0.07059	-0.28156	-0.1307	1.064346
0.306705	1.321371		-0.45333	0.425938	-0.31871	0.198492	2.407709	1.709707
0	0		0	0	0	0	0	0
1.286263	0.190756		0.813168	-0.35707	-0.31335	-0.44106	-0.05883	0.255848
1.777416	-1.12015		0.626358	-0.16112	-1.7985	-0.17436	4.780209	3.710514
2.3388	-1.54481	3.084693	0.238804	0.204016	-1.30098	-0.06867	5.100382	0.479641
1.851198	-1.5233	3.186679	1.013945	-0.69988	-1.62287	-0.47195	5.456518	1.903774
0.838068	0.602166	1.357953	1.737534	0.383406	-0.34869	0.607638	-0.32473	-2.76035
1.038643	1.188611	0.673285	1.737255	1.265875	0.477373	1.089961	0.681263	0.663336
1.650146	-1.03678	3.745984	1.605932	-0.50478	-0.82394	0.073897	4.622591	3.364872
1.370916	-2.17833	3.315976	0.692583	0.598614	-1.15001	0.099145	5.516539	-0.19826
1.981218	-1.32744	5.188476	1.928625	-0.201	-3.08954	-0.78011	6.340336	2.448786
3.311722	-1.62839	4.207038	-1.38507	-2.35964	-2.46638	0.27665	6.685545	0.655064
1.619427	-2.50147	4.96931	-0.71838	0.096853	-2.95249	-2.59065	7.287344	2.789034
2.003138	-1.123	3.409362	1.087776	-0.41751	-1.21717	0.542808	5.461582	1.389004
1.914873	-2.04486	4.82001	1.748438	0.249067	-2.63637	-0.16136	7.246729	1.42128
-0.19331	0.729316	2.23033	1.731071	1.096188	0.420609	-0.14708	0.41856	0.959774
-0.22695	0.614524	0.638238	0.477419	0.951208	0.466568	0.107013	0.47047	0.335083
1.522041	-3.31107	3.999705	1.450468	-0.28625	-2.10018	0.372663	6.105783	-1.34118
1.260208	-1.17589	2.511163	0.050514	0.646271	-0.62008	-0.53397	4.304363	-0.04172
0.708193	-2.18176	2.250936	-0.50635	-1.27155	-0.90175	-0.65889	2.587709	1.907967
-1.03864	-1.18861	-0.67329	-1.73726	-1.26587	-0.47737	-1.08996	-0.68126	-0.66334

Lung_VEGF	Lung_SERP	Lung_STAT	Lung_OASL	Lung_OAS2	Lung_OASL	Lung_OAS3	Lung_FOS	Lung_MX1
-2.25532	-1.20062	-0.29811	-0.63653	-0.52837	-0.07624	-1.23551	-0.66652	0.014009
0.20521	-0.99723	0.103857	0.615911	1.17403	0.777195	0.796541	-0.08331	1.151705
-1.33574	-0.55761	-0.30867	0.075024	0.500589	0.591736	0.716629	-0.2593	-0.58858
1.110395	-0.67363	0.435986	0.02564	0.130119	0.411043	-0.25927	-0.05845	0.346544
-0.28484	-0.13795	0.216385	1.125238	1.368671	0.799213	0.668318	-0.51401	-0.27088
-1.10476	4.381271	1.665787	3.180634	1.505859	2.074116	2.541866	0.465672	3.198833
0	0	0	0	0	0	0	0	0
-2.73628	4.301022	1.347277	3.198051	1.966993	2.070837	3.440439	-0.67916	2.390028
-1.66442	-0.73884	-0.3913	-0.74618	-0.49572	-0.32963	-1.12264	-0.34158	-0.53921
-2.4743	2.690945	2.140287	4.505756	2.506346	2.471949	3.095417	0.876358	4.970711
-2.33469	3.471811	1.356583	3.468866	2.002218	2.430719	3.418871	-1.60174	2.515692
-0.58231	0.039549	0.058992	0.373892	0.836727	0.076809	0.440111	1.750746	-0.42766
0.04497	0.743774	0.342148	0.187078	0.712738	0.475697	0.29772	1.143021	-0.40812
-0.63103	3.491032	2.515886	6.545265	3.185061	2.822908	3.971767	3.44165	6.321167
-2.32185	4.391897	1.497362	3.184113	2.427361	2.461298	3.240459	1.929714	2.564716
-1.92056	4.405962	2.11554	7.036224	2.944748	2.358932	4.340538	3.716238	6.000229
-0.43391	0.317064	0.038643	0.188856	0.500652	0.334988	-0.63326	1.544653	0.062103
-0.53659	4.571581	1.921352	4.246325	2.05529	2.932711	4.068764	2.874718	3.857328
0.20228	0.058704	0.561741	1.205669	1.068287	0.957706	1.235126	1.180984	1.447004
-0.37187	1.160564	-0.11058	-0.11476	0.857944	0.553724	1.63015	2.749489	-0.80583
0	0	0	0	0	0	0	0	0
0.073397	0.126076	-0.19437	0.605982	0.222996	0.220549	-0.21231	1.798815	-0.07649
-0.4289	4.723953	2.163992	4.305941	2.829391	2.920273	4.895182	2.535851	3.070181
-2.5795	4.685426	1.546764	3.682835	2.541822	2.983522	3.244979	2.655644	2.512338
-3.22433	4.552865	1.867785	3.632946	2.410328	2.764263	2.418006	2.185529	2.65167
-0.59593	1.012351	1.434368	1.136503	1.38582	1.535299	0.717496	2.450997	1.837174
0.504402	0.737296	0.825563	0.990917	0.958347	0.530523	0.67463	0.994134	0.848147
-1.12155	5.93101	2.558447	3.855085	2.90078	3.437714	1.843331	1.857865	3.234567
-1.56607	4.671979	1.752056	2.957809	2.191259	2.620207	1.997191	1.258086	2.472018
-1.95569	6.258279	5.445702	7.325218	5.376686	5.721621	3.173686	4.815396	6.558381
-2.53528	4.990552	4.053436	7.869223	4.139456	3.745918	3.729926	4.769273	7.433282
-1.71306	5.515738	2.373362	3.565256	3.267864	3.161163	2.016181	2.898126	3.19711
-1.01324	4.923808	2.254465	2.383436	2.338133	2.930025	2.037532	1.039895	1.734712
-0.40333	4.5677	3.371281	4.247047	2.584507	3.396654	2.741635	2.425221	4.758327
0.862383	2.339847	3.061172	2.264214	2.927406	2.675659	0.705777	2.248446	0.751576
-1.10408	0.159484	1.231421	1.393646	1.281086	1.369051	-0.90296	0.242871	0.634728
-1.38346	4.899325	1.468987	3.74535	1.60515	2.171541	3.271285	2.714145	3.614207
-3.70331	4.568688	1.890629	3.414524	2.521643	2.875835	3.117669	0.994407	2.534532
-2.35034	4.688132	1.125086	2.875856	2.208115	2.190144	2.257794	0.681565	1.794206
-0.5044	-0.7373	-0.82556	-0.99092	-0.95835	-0.53052	-0.67463	-0.99413	-0.84815

Lung_IL18	Lung_IRF1	Lung_IRF7	Lung_IRF8	Lung_CXCL	Lung_CXCL	Lung_IFIH1	Lung_DDX5	Lung_KLF2
0.322763	0.091566	-0.13216	-0.13342	-1.65371	1.809511	0.26893	-0.15213	-0.41366
0.317965	0.063927	0.513094	0.281738	-0.61478	1.032038	0.467869	0.21245	-0.71003
0.617321	-0.00742	-0.19653	-0.68502	0.055487	0.113106	0.075428	-0.02247	0.130033
0.047571	0.533134	0.324574	0.231766	-1.82571	0.296251	0.520851	0.459885	-0.02106
0.209225	0.27931	0.608604	-0.33717	0.125412	-0.14164	0.434898	0.40444	-0.24352
-0.64494	0.874138	3.586357	0.504564	4.912693	9.213177	1.37216	0.698244	-0.39483
0	0	0	0	0	0	0	0	0
-0.80796	0.833914	3.231615	-0.30738	5.272484	8.23378	1.156273	0.954697	-0.66678
0.180542	-0.21646	-0.56178	-0.36162	-1.39461	-1.0628	-0.0125	-0.34069	-0.6722
-1.20571	1.917509	4.114174	0.955481	5.345842	10.72459	1.871796	1.218136	-0.3526
-0.29411	0.704407	3.427227	-0.4835	4.658302	8.699492	1.334953	0.857353	-0.97876
-0.14849	0.090096	-0.42433	0.283827	-1.235	-0.59643	0.503395	0.861765	0.079369
-0.48893	0.076918	-0.20847	0.120121	-0.29892	-0.30324	0.562923	0.836254	-0.44392
-0.85732	2.723038	3.990625	1.798109	9.017725	10.85167	2.337395	2.109247	1.340347
-0.33286	0.581955	3.968485		7.382212	7.285721	1.399206	1.314837	-1.28638
-1.15537	1.824093	4.391897	1.659199	11.25144	10.85554	2.090113	1.734888	0.757179
-0.07276	0.029924	0.557196	0.126518	0.475266	-0.74554	0.225557	0.371172	-0.90206
-0.4953	1.375633	4.046986	0.883257	8.012733	10.72135	1.609838	1.219316	1.936146
0.661978	-0.01838	1.453131	0.22127	2.494762	0.443451	0.517029	0.283974	1.502903
-0.861	0.106644	-0.19848	-0.50809	3.351601	-1.05272	0.189196	0.113379	0.74283
0	0	0	0	0	0	0	0	0
0.458378	-0.31367	0.076651	-0.18803	1.275023	-0.51393	-0.1172	-0.16991	0.685184
-0.73705	1.057592	3.576998	0.532995	7.173359	9.711451	1.930325	1.957624	2.313793
-0.01406	0.623747	4.25771	0.219824	2.274023	5.732772	1.268022	1.112663	2.416012
-0.78798	0.422405	3.966089	0.286589	3.074734	5.176711	0.981742	0.711203	-0.54845
0.749286	-0.6414	0.128128	-0.59156	-0.60296	0.033772	-0.27476	-0.43695	0.205532
0.672072	-0.23629	0.093878	-0.29718	0.837635	0.434434	-0.40918	-0.30779	-0.11786
0.100644	0.119225	3.543144	-0.21478	1.346941	3.660798	0.794337	0.489363	1.257046
-0.70898	0.017056	3.57662	-0.07338	2.479713	5.016572	0.732892	0.150713	-0.39227
0.746274	1.570963	3.386606	1.562729	4.858551	6.482692	2.284846	1.554861	-4.23071
-0.13837	2.969131	2.794878	2.79545	6.270304	8.006883	3.03807	2.77446	2.961834
0.099704	-0.2676	2.877684	0.255318	4.007782	4.80618	0.806878	0.346273	2.129389
0.174607	0.041809	3.639488	0.071255	1.228159	3.66735	0.592637	0.345196	-0.66261
0.156253	1.371668	3.878004	1.088137	4.345003	6.568391	1.485425	1.296049	3.153347
	0.235857	0.670759	-0.02005	-1.43229	0.267838	0.24071	0.226742	2.796049
0.349273	-0.52015	-0.04786	-0.53661	-0.60528	0.455205	-0.3042	-0.67141	1.776672
-1.44006	1.470282	3.795326	2.209362	5.463575	6.876754	1.225192	0.846418	2.61795
-0.14618	0.268057	3.998457	0.069086	1.998632	3.834901	0.592074	0.706936	2.235977
-1.22611	0.310179	3.599628	-0.1628	1.960403	3.941318	0.708371	0.661503	1.272533
-0.67207	0.23629	-0.09388	0.297176	-0.83764	-0.43443	0.409183	0.307791	0.117859

Liver_ICAM	Liver_SELE	Liver_IL6	Liver_SOCS	Liver_STAT	Liver_CEM	Liver_F3	Liver_RHO	Liver_TSP1
0.134438	0.192612	-1.53181	-0.03926	-1.00434	-2.35918	2.305107	1.435249	0.045464
-0.58114	-0.04835	0.036179	0.763916	0.333153	0.808102	0.760393	0.225193	1.00596
0.022757	0.452932	-0.55338	0.680624	1.436432	-2.10987	0.430883	1.68655	-0.29113
0.447216	-1.18509	0.704247	-0.1163	0.232601	-2.38444	1.719448	1.242739	0.660194
-0.23057	-0.4091	-0.53659	-1.76798	-0.27834	0.660267	-0.38177	0.978613	0.766232
2.85297	0.937992	3.774815	4.927717	1.062023	5.705448	-1.2565	-0.18683	0.853441
0	0	0	0	0	0	0	0	0
0.629782	-0.05797	0.043112	-0.05425	0.398714	0.940287	-0.54966	0.561653	-0.43678
2.239267	-0.09941	2.382437	3.601738	0.425369	4.835032	-1.41057	-0.6404	-0.18639
2.993464	0.462931	3.840715	4.85465	1.050423	6.01259	-0.8243	-0.5229	0.268681
1.867887	0.41749	1.251749	3.269682	-0.44289	2.197523	-2.60499	-1.39635	-1.48703
0.437616	-1.04453	1.302492	3.141729	0.930691	1.081533	-0.91315	0.556438	-1.91973
0.972414	-0.80232	1.494547	3.22089	1.103371	2.268457	-0.53333	1.520699	0.349491
3.928272	2.680834	6.199553	6.429426	1.487377	5.852024	-0.62277	-0.38333	1.433561
2.978481	1.213512	2.487476	4.946543	0.750921	1.159672	-1.04435	0.415701	-0.35608
3.916895	2.35931	7.003735	6.466637	1.873302	6.095951	0.113077	0.405239	3.010353
0.018827	0.129225	-0.13206	1.488516	0.07802	0.587084	0.182892	0.193346	-1.64026
3.407925	0.230425	6.700598	5.954681	0.767387	5.562553	-1.18797	0.034468	0.473499
1.170818	-1.78465	0.32552	0.51012	0.184792		0.232107	0.338764	-1.17487
-1.6118	-0.67191	0.276915	4.253107	0.261902	5.268505	-1.3781	-0.36317	0.035486
0	0	0	0	0	0	0	0	0
0.013939	-2.5404	0.190319	1.961992	0.142563	-0.15932	-0.06266	0.104622	-1.88965
2.277514	-0.02656	4.129902	4.983885	-0.23649	2.698072	-2.91407	-1.10175	-1.4474
1.909697	0.769535	2.25588	2.788642	-0.16633	-1.99817	-1.35896	-1.14001	0.873483
1.082699	1.629038	1.389565	3.302098	0.463883	0.978291	-2.13811	-1.68262	1.207543
-0.06426	0.970028	0.390978	0.966641	0.294555	0.881247	-0.91856	0.929865	1.492429
0.4688	-1.60938	-0.71508	-0.105	0.199508	0	-0.37132	-0.32291	-0.36025
2.578995	1.023035	1.626858	1.424767	-0.9806	-3.13028	-1.59442	-1.79834	-0.14371
2.528852	2.140991	3.5142	3.253379	0.055617	-0.58946	-0.48694	-0.45655	0.804345
4.491749	3.958673	4.874016	3.812186	-0.28203	2.933744	-1.17086	-1.94436	1.661738
4.873938	5.645809	7.026979	6.352304	1.760079	4.984541	0.554679	1.562222	4.03756
2.160143	1.510172	2.361444	2.817124	-0.70692	2.061031	-1.32832	-1.55456	1.239253
0.4582	1.297699	1.286745	2.310676	-1.65597	-1.5648	-1.90402	-1.95471	-1.55727
3.347286	1.846107	3.832672	3.705352	-0.81701	2.067366	-0.26049	-1.73685	1.385262
0.305685	1.323917	1.776052	1.230468	0.145551	0.246775	0.321675	0.236273	0.767627
0.306412	-0.6555	0.73592	1.392337	-0.07084	-1.85938	1.161414	-0.85873	-0.33203
4.419186	2.923441	6.092335	5.723077	0.844367	4.657385	-0.32996	1.041618	2.252389
2.499973	1.145319	2.992512	3.999314	0.262382	0.776707	-0.85224	-0.72132	0.946374
0.692875	1.793409	1.539165	3.606835	0.084952	-2.20616	-1.28686	-0.78449	0.739831
-0.4688	1.609385	0.715084	0.104999	-0.19951		0.371324	0.322906	0.360246

Liver_PLAT	Liver_PLAU	Liver_THBD	Liver_PLAU	Liver_ET1	Liver_PTGS	Liver_VWF	Liver_PRO(	Liver_CD44
0.600952	0.633568	-0.12667	-1.00417	0.107891	-1.3258	1.419785	-0.496	-2.89417
0.298569	0.601242	-0.56931	2.069584	1.632851	1.106098	2.340246	0.355541	-0.31989
-1.53143	-2.32231	0.711897	-0.31993	1.105143	-1.0765	1.068254	-0.98574	-2.38693
-1.90229	-1.24501	-0.5092	-1.30728	0.337505	1.523796	0.212078	1.671022	-1.92597
-0.41386	0.141804	1.341091	0.01083	0.862736	1.111481	1.640676	-0.96684	-2.16986
1.386497	-0.9319	2.704594	3.742693	2.79525	3.577797	-1.44049	3.175478	1.371834
0	0	0	0	0	0	0	0	0
0.967131	0.367376	-0.33932	-0.26277	1.612434	1.999506	1.176706	1.454575	-0.20961
0.399065	-0.43217	2.449608	2.338251	1.281984	3.07733	-1.08901	3.434866	-1.74804
1.221567	-1.3624	1.227701	3.074333	2.496693	6.641222	-0.64499	3.389751	0.633095
-1.65137	-3.0874	1.147249	1.136635	0.28796	1.68371	-1.37528	3.808889	0.759605
1.101118	-1.46323	-0.17797	-1.20107	1.287678		1.293224	-0.63157	
0.669941	-0.28563	1.049576	0.12813	1.501245		0.665695	-0.54632	
-0.49422	-0.7767	2.23794	4.770363	3.292469		-0.28324	-0.07425	
2.435057	0.324707	2.311651	2.233738	1.578081		-0.30788	3.159504	
0.805717	-1.29817	1.679293	5.409969	5.768402		1.112383	4.194666	
0.304462	-1.06288	0.337868	-1.80176	0.372641		-0.65922	0.786434	
0.235992	-0.93458	1.231443	3.224895	1.993525		0.074518	4.820974	
0.146914	1.125637	0.559374	-0.14348	1.058382		1.192116	1.040665	
-0.56327	-1.20153	1.462076	1.090467	-0.24836		0.948254	1.624664	
0	0	0	0	0		0	0	
0.519361	-0.88229	-0.21919	-1.58746	0.146835		0.292976	0.272509	
-0.03434	-2.90153	1.777735	1.690693	0.43206		-2.2236	2.247162	
0.442167	-1.98882	2.089911	0.334362	0.670271	0.500095	-2.42385	3.380147	-1.74046
0.43618	-0.69048	1.415562	0.784479	-0.52066	1.967066	-2.44417	1.857547	
-2.68043	0.536463	0.462574	-1.74683	-0.97541	-0.23101	0.200583	0.429561	
-4.64118	-1.99241	0.644686	-2.81395	-0.29762	-0.25319	-0.10852	0.202548	0.096145
-0.67128	-2.53201	0.898802	0.070528	0.779289	-0.26907	-2.8743	2.643537	-3.40917
3.701214	-1.59509	2.456373	1.414236	1.725229	2.191856	-0.79595	4.207513	-1.51178
-0.71317	-0.97581	2.226681	1.860922	2.300667	4.929186	-1.70544	4.474847	-0.42305
3.207153	0.030115	2.415071	4.809357	4.300032	7.673098	1.519559	3.784446	2.192163
0.657698	-2.30319	0.789532	1.137157	0.510891	1.510393	-2.4264	2.253631	-1.52577
2.480835	-3.17033	1.60754	-0.78212	-0.05242	-1.21291	-3.54283	1.903354	-3.33631
3.664017	-1.77638	1.331993	1.927662	1.76383	1.754753	-0.47045	4.819821	-1.55527
3.667269	-0.38236	1.454617	-1.38397	-0.34768	-2.92538	0.42397	1.016345	-0.53443
0.675585	-1.79737	-0.31541	-1.93572	-0.74416	0.923826	-0.29876	-0.00876	
3.607878	-0.90459	2.688269	2.310381	3.357351	5.694397	-1.08036	4.436751	-0.16339
2.920835	0.279652	1.576521	-0.11691	0.828171	0.87295	-0.76062	3.091054	-4.11112
3.280098	-0.94452	2.543079	0.314302	0.456161	0.259859	-1.36373	2.74347	
4.641176	1.992409	-0.64469	-1.38647	0.297623	0.253187	0.108518	-0.20255	-0.09614

Liver_TFPIA	Liver_TFPIE	Liver_TFPI(	Liver_SELP	Liver_C5AR	Liver_VEGF	Liver_SERP	Liver_STAT	Liver_OASL
0.611694	0.754799	0.637129	0.954575	-0.69623	0.721827	-0.63098	2.472965	0.675558
1.248743	1.276073	0.705894	4.514338	2.170406	-0.02917	0.539871	1.665136	1.954882
0.560665	1.049503	-0.27603	2.233942	0.074013	0.603123	-1.10657	2.552603	0.57795
2.424864	2.566338	2.086939	4.000574	-0.77014	-0.72399	0.274385	2.130299	-0.68539
1.297453	0.933544	0.124998	3.572058	-1.07566	-0.04918	-0.68032	1.248423	1.506887
-0.00723	-1.48107	0.592825	6.631907	-0.18237	-0.02999	8.560869	3.858919	0.865887
0	0	0	0	0	0	0	0	0
0.503	1.581493	0.242441	1.95363	1.708858	-0.8313	-1.11501	0.973803	-0.75323
0.825323	-0.97623	0.546139	5.094646	2.33662	-0.64458	5.106773	1.510977	-0.02323
-0.2746	-3.06705	-0.68777	5.509991	1.760544	0.108194	6.552444	0.550056	1.780815
0.923044	-0.21567	0.841986	4.158083	0.802757	-1.59126	7.171038	-0.55769	-0.83426
-0.05074	0.215391	0.294884	3.36586	1.987726	0.85083	0.003914	0.891939	0.620514
0.144218	0.53273	0.428709	4.846956	1.821552	1.91284	0.343115	-1.08555	-0.86326
-0.15539	-2.1222	0.669291	7.603731	4.553307	1.643305	9.058363	2.464266	3.472616
-0.0265	0.78301	1.389853	7.342264	4.098963	2.18693	3.716837	2.465879	1.467045
0.675835	-1.45305	1.160282	9.12921	5.280737	2.423498	8.602314	-0.29382	3.455431
-0.51528	-0.95635	-0.15774	1.345095	0.598221	0.683889	-1.82019	-0.31513	-0.49298
-0.12326	-1.3142	0.920788	4.607075	2.663584	2.94463	7.646662	1.923384	1.570236
1.705194	0.295151	0.906168	1.476109	1.337181	1.041546	0.393705	0.660191	1.182291
0.962639	0.63187	0.769381	6.717472	1.218348	2.801851	5.981428	1.392641	-1.85342
0	0	0	0	0	0	0	0	0
0.048599	-0.05196	-0.34395	0.785397	1.272167	2.050352	-0.41254	0.114439	0.73488
-1.53289	-3.10555	-0.97256	6.550749	3.716082	0.750803	7.229824	0.557978	-0.77261
-0.08363	-0.84114	0.380824	4.165033	2.741943	-1.80658	5.202973	-0.09021	-0.49175
-0.57539	-2.81593	-0.26935	5.336552	3.373051	-0.19267	6.93678	-0.43377	-1.5122
-1.07965	-0.04783	-0.28773	-0.28484	0.069815	0.225626	-0.12383	-0.23991	0.555232
-0.13448	-0.41194	-0.80418	-1.65012	-0.2707	-0.62355	-0.26962	-0.00699	-0.39415
-0.68535	-1.51392	-0.11477	2.041159	1.418716	-0.62462	6.352029	-0.32967	-0.82481
0.000823	-0.46819	0.504619	3.12553	4.105886	-0.97347	7.062757	-0.25216	0.043351
-0.38754	-3.06754	-0.16924	2.230228	5.088167	-2.7119	8.48767	0.192434	1.640121
-0.12952	-1.58958	-0.48182	7.018087	5.665831	2.967979	10.22461	-0.08269	3.976336
-1.15953	-3.05753	-1.28814	0.433176	2.695938	-2.09249	6.664779	-0.94958	-1.38082
-0.44466	-2.4424	-0.3662	3.066576	3.092417	-1.12948	4.403232	-0.5286	-1.55947
-0.38379	-1.84776	-0.08579	4.721973	4.871653	-1.55375	7.294583	-0.05372	0.734393
-0.10475	0.551761	0.389134	0.49971	4.165392	-1.06024	-0.42652	0.048973	-0.09304
-0.21279	0.699521	0.235269	1.22068	2.415421	-1.7237	-0.07362	0.480299	-0.17039
0.350476	-2.2771	0.746257	4.969149	4.579374	1.020784	10.48785	0.101147	0.82277
0.716058	-0.35904	0.422439	3.477304	1.834843	0.929445	6.350297	0.836262	0.229877
0.180354	-0.45637	0.386575	5.411156	4.294973	0.278849	7.649146	0.325243	-0.57246
0.134477	0.411937	0.804175	1.650122	0.270699	0.623549	0.269622	0.006992	0.394151

Liver_OAS2	Liver_OASL	Liver_OAS3	Liver_FOS	Liver_MX1	Liver_IL18	Liver_IRF1	Liver_IRF7	Liver_IRF8
0.263638	-0.05541	2.189655	-3.76981	-0.38907	0.618977	0.428713	0.20355	0.087296
0.55526	1.061731	2.250204	-2.13139	1.556696	0.026621	0.504925	0.440355	0.657644
0.406752	-0.02647	0.933704	-2.68115	0.351242	0.227467	0.201773	0.047907	0.512527
-0.38584	-0.88198	0.84864	-3.23645	-0.83873	0.122622	0.236923	0.155525	0.242025
0.461632	1.156136	2.609543	-5.6049	0.326237	0.752586	-0.10711	0.801764	1.001278
0.654112	0.212114	1.228096	-7.09229	1.002728	-2.29422	-0.54348	0.717768	1.029026
0	0	0	0	0	0	0	0	0
-1.09322	0.995171	-0.34607	-5.63383	-1.16491	0.167553	-0.03281	-0.22746	-0.05546
0.399714	-0.03307	1.751421	-6.94519	-0.06686	-2.49978	-1.38012	-0.44402	-0.03449
-0.67801	-0.86408	1.755795	-7.42485	2.388548	-2.51131	-0.14664	0.449669	1.392389
-2.95863	0.03278	2.815296	-8.82858	0.336687	-3.23633	-1.37411	-0.57808	-0.67436
0.383776	-0.08679	-1.85488	1.913801	2.649532	1.203314	1.437572	1.694626	1.152765
1.254869	0.471539	1.279982	3.70846	2.633097	1.554155	1.229675	1.282055	1.814585
2.684561	3.132298	1.615671	2.895691	6.196445	-0.84164	2.595547	1.756008	3.797964
3.441557	2.982155	2.383125	2.972982	3.043047	0.059507	1.478048	3.235502	0.478674
3.911779	3.228922	3.517769	4.157698	7.487839	-0.03062	1.298847	2.959263	5.418222
-1.85107	-1.6046	-1.08947	-0.68664	1.607481	-0.83149	-0.13695	0.516047	2.460382
1.629677	2.753458	2.84947	2.377207	3.272242	-1.92571	1.076981	1.37785	3.300781
-1.58373	1.439886	-0.00628	0.056389	1.243088	0.468941	0.03779	1.674004	2.036499
1.177998	-0.90393	-0.44821	5.052406	-0.77249	-1.12013	-0.38505	-1.6755	0.89826
0	0	0	0	0	0	0	0	0
-0.50718	-0.3661	-1.34338	0.982243	1.672279	0.174484	0.477631	1.162279	1.139967
-1.12042	1.200552	1.056467	1.033823	0.818048	-2.54311	0.316986	0.584867	1.177933
0.175931	1.392542	2.365132	1.643565	0.229054	-1.83582	-0.87153	0.638165	0.355429
-0.65134	0.621187	1.27476	0.40805	-0.1777	-3.49022	-1.23038	0.27061	0.257524
-1.05671	-0.23248	-0.50122	1.955228	0.393567	-0.63619	-1.12189	0.227269	-0.03161
-0.132	-0.2062	-0.50521	-0.53134	0.055879	-0.24563	-0.0521	-0.12818	-0.73134
-0.17231	0.980799	1.385676	-0.21216	0.090009	-2.33828	-1.50319	0.25426	0.007425
0.8474	1.633303	2.527806	1.519811	0.395127	-1.40647	-0.68341	0.57368	0.009327
1.336131	2.438147	3.590957	1.357493	3.275487	-2.35629	-0.1309	0.789775	1.412634
1.476232	1.732159	3.052238	2.928842	6.174495	-1.82975	0.760052	0.265626	2.325821
-0.85074	0.254047	0.88403	-0.3265	-0.79827	-2.68772	-1.90343	-0.71905	0.095993
-1.13937	0.029556	0.214923	-0.71539	-0.36822	-2.51198	-1.58844	-0.07696	-0.68338
0.881007	1.875128	3.142478	1.060994	2.862903	-1.78495	-0.18832	1.000504	1.859867
0.795737	0.576372	1.623467	1.060023	0.53095	0.663541	0.401077	0.92266	1.187653
-0.30894	0.458679	-0.82501	-0.07677	0.605592	0.012803	0.35511	0.44106	-0.29351
1.075198	1.794348	2.641883	2.727439	2.37485	-1.71926	0.225615	0.801641	2.414667
0.759057	1.767649	2.485824	0.621421	1.27192	-1.73858	-0.00817	1.225448	0.99246
0.122937	1.009657	1.889344	0.310513	0.633729	-2.00494	-0.61207	0.631692	0.034807
0.132001	0.206196	0.505214	0.531341	-0.05588	0.245633	0.052095	0.128184	0.731344

Liver_CXCL	Liver_CXCL	Liver_IFIH1	Liver_DDX5	Liver_KLF2	Liver_HNF1
-1.36317	0.95731	0.493565	0.308855	-0.13679	0.080507
0.041475	1.577154	0.533447	0.490557	-0.18397	0.33322
-0.03035	0.667221	0.433825	0.903616	1.777164	0.365881
-0.54302	0.633476	0.283134	0.101345	-0.43969	-0.34381
-0.46115	1.450491	0.866093	1.004646	1.122276	0.592606
3.738552	7.065247	-0.90606	-0.88185	0.873407	-0.13396
0	0	0	0	0	0
0.401836	-0.26968	-0.11662	0.183739	-0.04109	-0.2346
2.021235	4.49819	-1.76628	-1.39098	-0.41855	-0.68668
4.091976	6.609978	-0.88219	-1.3539	3.355675	-0.65572
1.934416	5.943825	-1.72341	-1.56607	1.573938	-1.50224
	-1.2644	-0.04898	-0.21921	1.710983	1.357626
3.159487	0.033834	-0.00281	0.361795	1.526342	0.733961
8.449066	8.933098	0.514673	0.18961	2.018227	0.872683
4.861778	6.590187	-0.39037	0.553297	1.25835	1.07439
11.13791	8.969803	0.700928	-0.13718	3.248339	1.116924
2.374268	-0.83018	-0.21305	-0.69618	-1.63671	-0.69147
7.964956	6.785204	-0.86966	-0.7119	1.812347	0.801331
3.246798	5.739449	0.220364	0.396957	0.447346	0.340843
3.179937	0.697405	-1.23088	-0.689	0.658049	0.253586
0	0	0	0	0	0
0.312546	-0.74778	0.029316	0.099384	-0.00834	1.070303
6.362776	5.742056	-2.02203	-2.01428	0.068415	0.443659
3.360962	7.028597	-0.75185	-0.17036	-0.00402	-0.61358
3.601076	7.075659	-1.5734	-0.71452	0.037589	-0.38415
1.80909	-0.59017	-0.27417	0.715085	0.233699	0.369961
0.179132	-1.03304	-0.09097	-0.77987	-1.18731	-0.66865
3.093073	5.337986	-1.19285	-1.38284	0.703225	-1.8714
4.215965	5.568108	-0.99617	-0.57017	0.794822	-0.48407
5.256285	6.661079	-0.09453	-0.92314	1.202609	-1.24349
8.724308	9.062132	0.6536	1.219142	3.006494	-2.15382
3.624567	5.884689	-1.65023	-1.54404	0.247867	-0.81222
1.9643	5.658554	-0.93416	-1.3366	-0.33811	-1.3996
4.794151	5.966402	-0.70274	-0.74988	0.805102	-0.58692
1.223915	1.16453	0.351409	0.802518	0.200927	0.367935
1.401865	0.1637	0.446347	-0.48024	-1.54806	-0.23161
7.000698	7.244959	-0.25274	-0.35166	1.792283	0.621481
4.316435	6.444992	-0.14921	0.614669	-0.03131	-0.03227
3.770933	6.217987	-0.76416	-0.35832	0.064776	0.040079
-0.17913	1.033035	0.09097	0.779868	1.18731	0.66865

Uncropped Western blot images for Figure 5D

pY705-STAT3

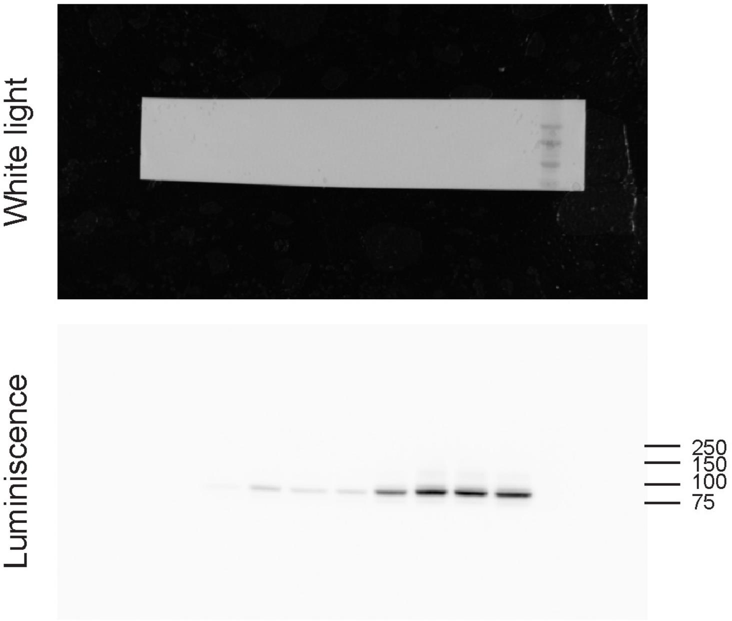

STAT3

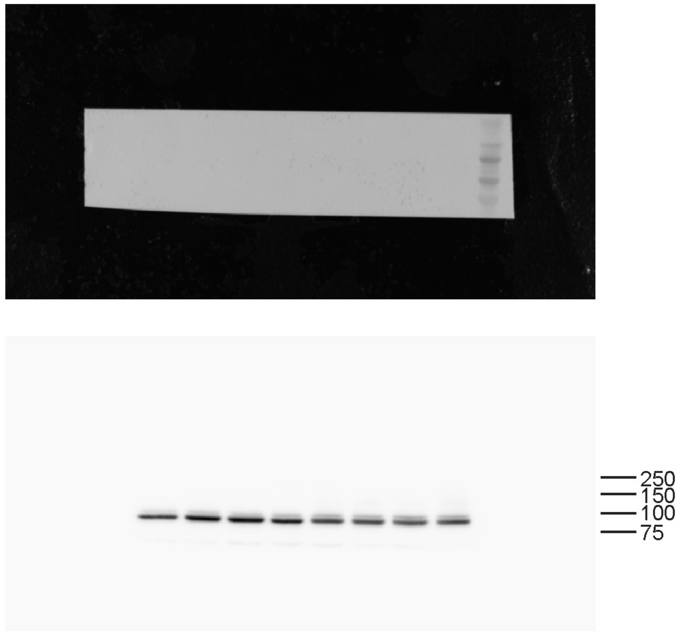

SOCS3

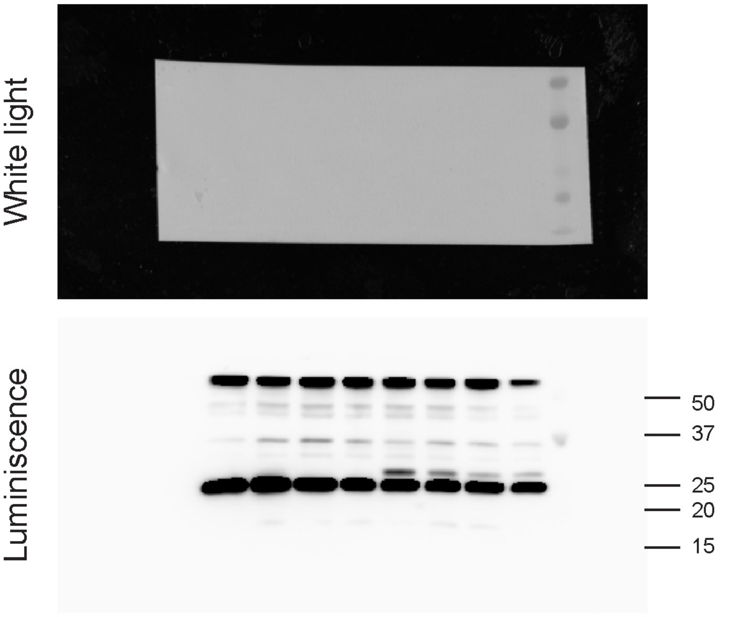

Actin

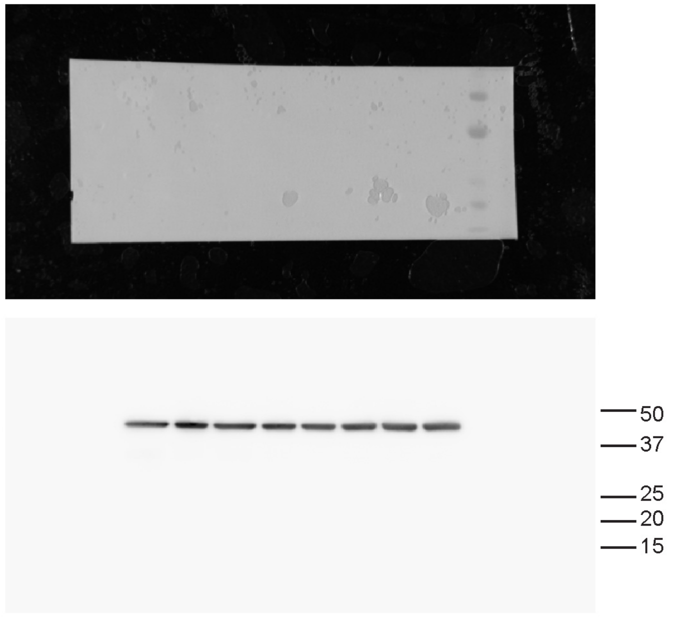

Uncropped Western blot images for Figure 6A

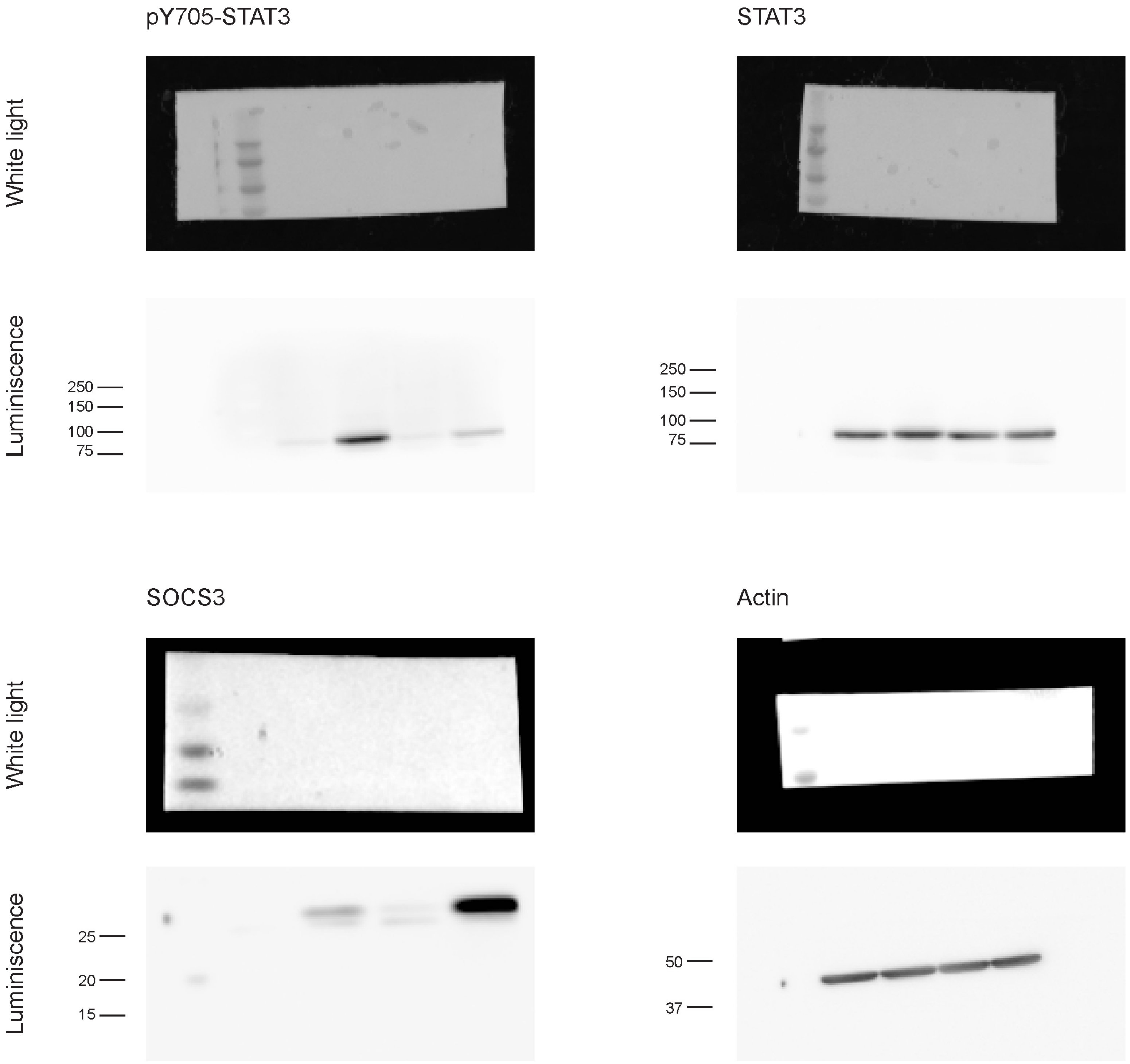

Uncropped Western blot images for Figure 6B

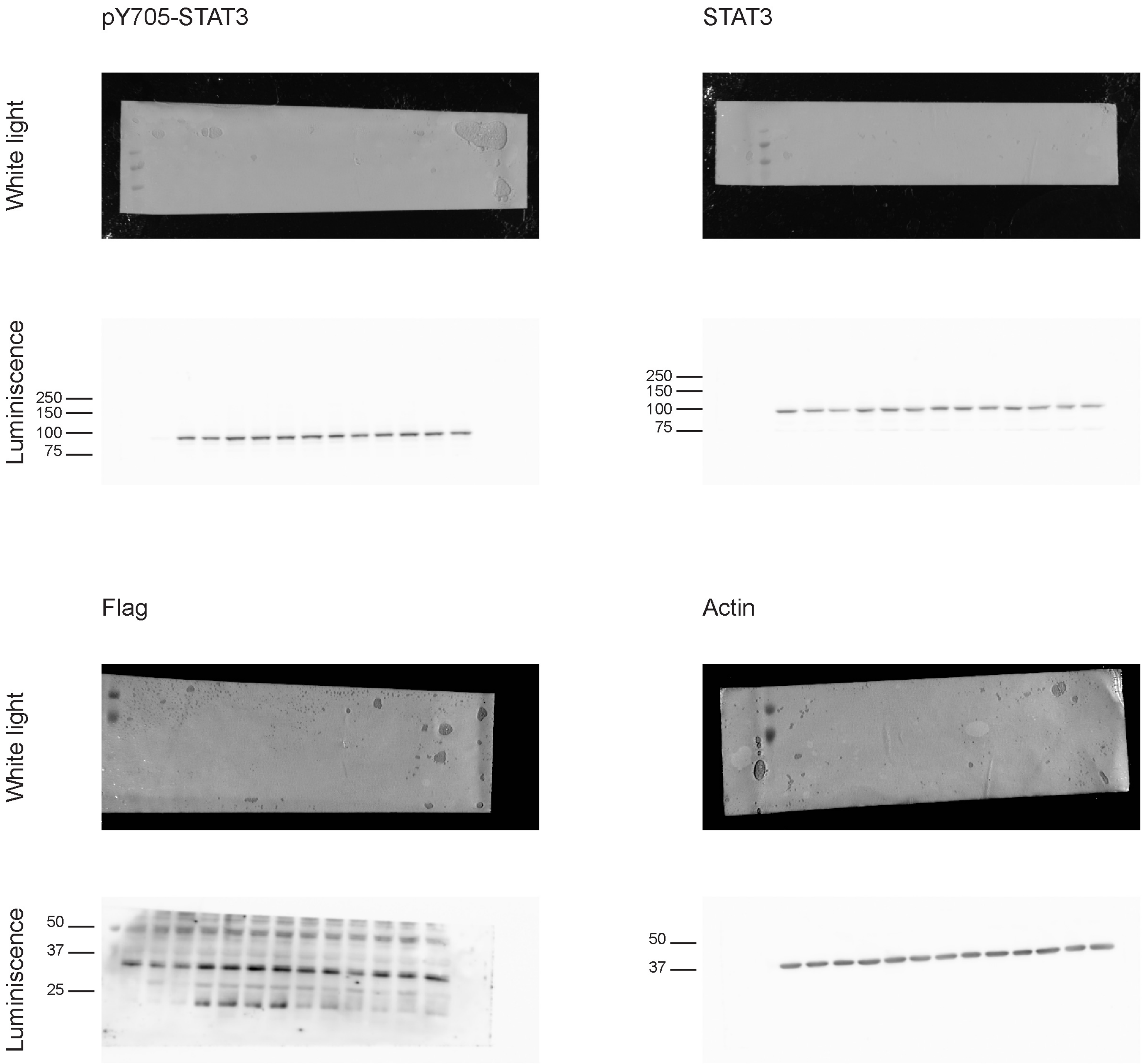

Uncropped Western blot images for Figure 6D

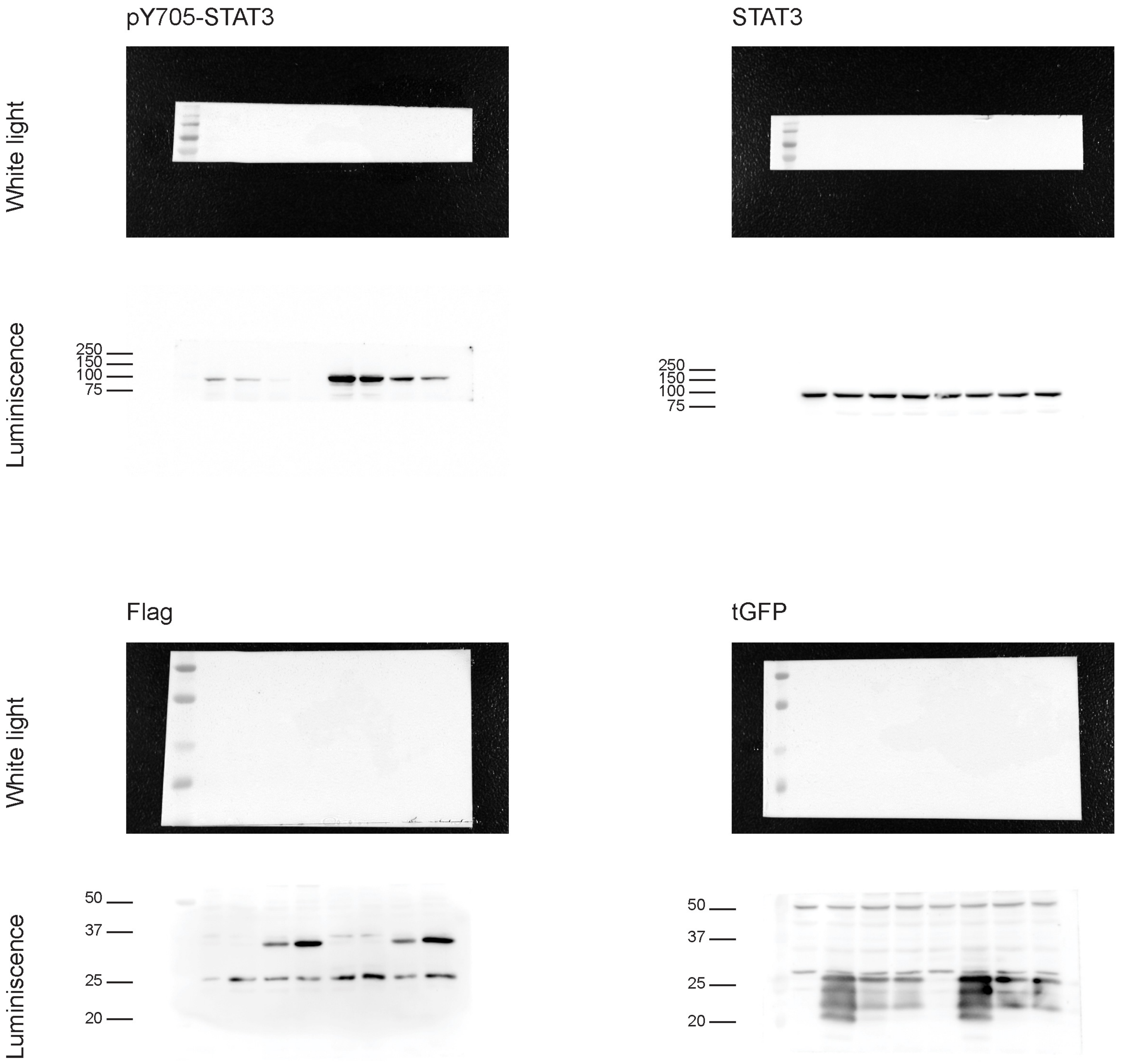

Supplement: Supplemental data [file jciinsight-6-147280-s024.pdf]
